# Supplementary material for: Strain-Specific Features of Primary Metabolome Characteristic for Extremotolerant/Extremophilic Cyanobacteria Under Long-Term Storage
Source: Int J Mol Sci. 2025 Feb 28;26(5):2201. doi: 10.3390/ijms26052201 (PMC11900582; doi:10.3390/ijms26052201)
Supplement: Supplementary file 1 [file ijms-26-02201-s001.zip › Supplementary_Information_S1 (Materials related the cyanobacteria identification).pdf]

Strain-Specific Features of Primary Metabolome Characteristic for Extremotolerant/Extremophilic Cyanobacteria Under Long-Term Storage

Tatiana Bilova[1,2*], Nikita Golushko[1,2], Nadezhda Frolova[1], Alena Soboleva[1], Svetlana Silinskaia[1], Anna Khakulova[3], Anastasia Orlova[1], Maria Sinetova[4], Dmitry Los[4] and Andrej Frolov[1*]

Supplementary Information S1

[1] Laboratory of Analytical Biochemistry and Biotechnology, K.A. Timiryazev Institute of Plant Physiology RAS, 127276 Moscow, Russia;

[2] Department of Plant Physiology and Biochemistry, St. Petersburg State University, 199034 St. Petersburg, Russia;

[3] Chemical Analysis and Materials Research Core Facility Center, Research Park, St. Petersburg State University, 199034 St. Petersburg, Russia;

[4] Laboratory of Intracellular Regulation, K.A. Timiryazev Institute of Plant Physiology RAS, 127276 Moscow, Russia;

* Correspondence:t.bilova@spbu.ru (T.B.); frolov@ifr.moscow (A.F.)

Directory

Figure S1-1. Some of extremotolerant and extremophilic cyanobacteria strains investigated in the study 3

Figure S1-2. Maximum likelihood phylogenetic trees based on partrial 16S rRNA gene sequences 4

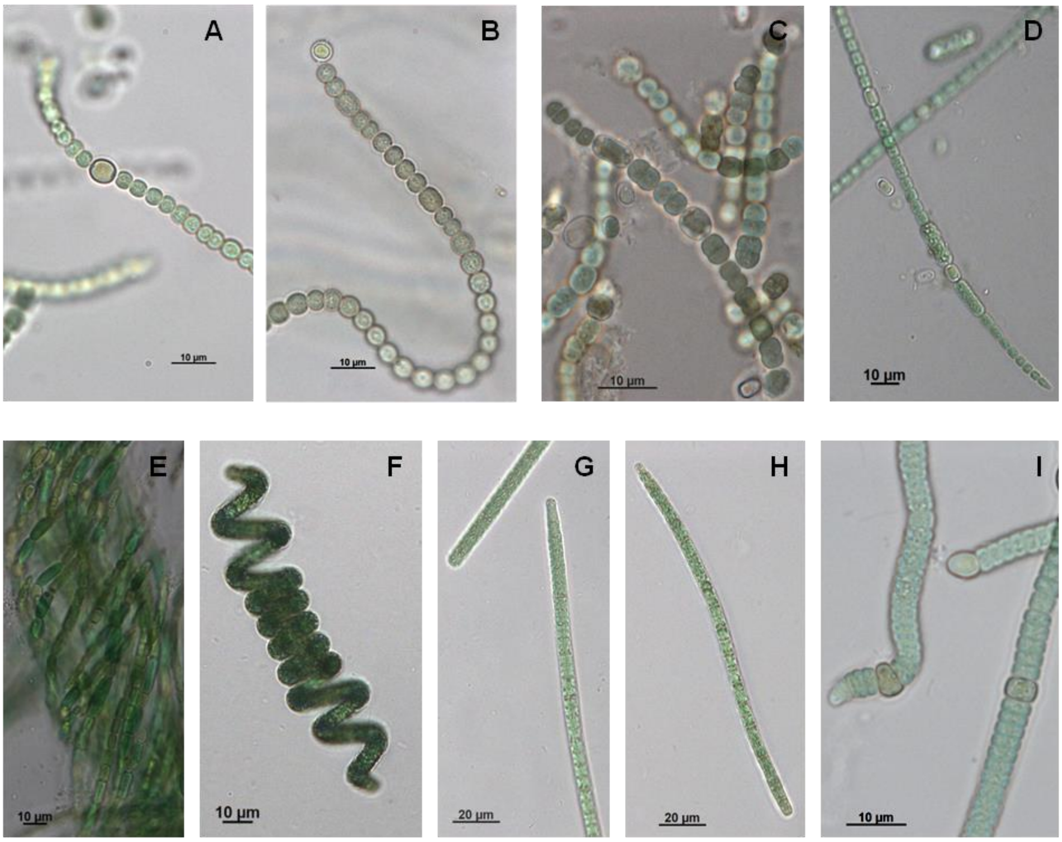

Figure S1-1. Some of extremotolerant and extremophilic cyanobacteria strains investigated in the study: A – *Nostoc commune* IPPAS B-1520; B - *Nostoc commune* IPPAS B-1519; C - *Dolichospermum* sp. IPPAS B-1213; D - *Anabaena* cf. *pirinica* IPPAS B-1533; E - *Anabaena sp.* IPPAS B-1535; F - *Limnospira* sp. IPPAS B-1526; G - *Limnospira* sp. IPPAS B-287; H - *Limnospira* sp. IPPAS B-256; I - *Nodularia sp.* IPPAS B-1529

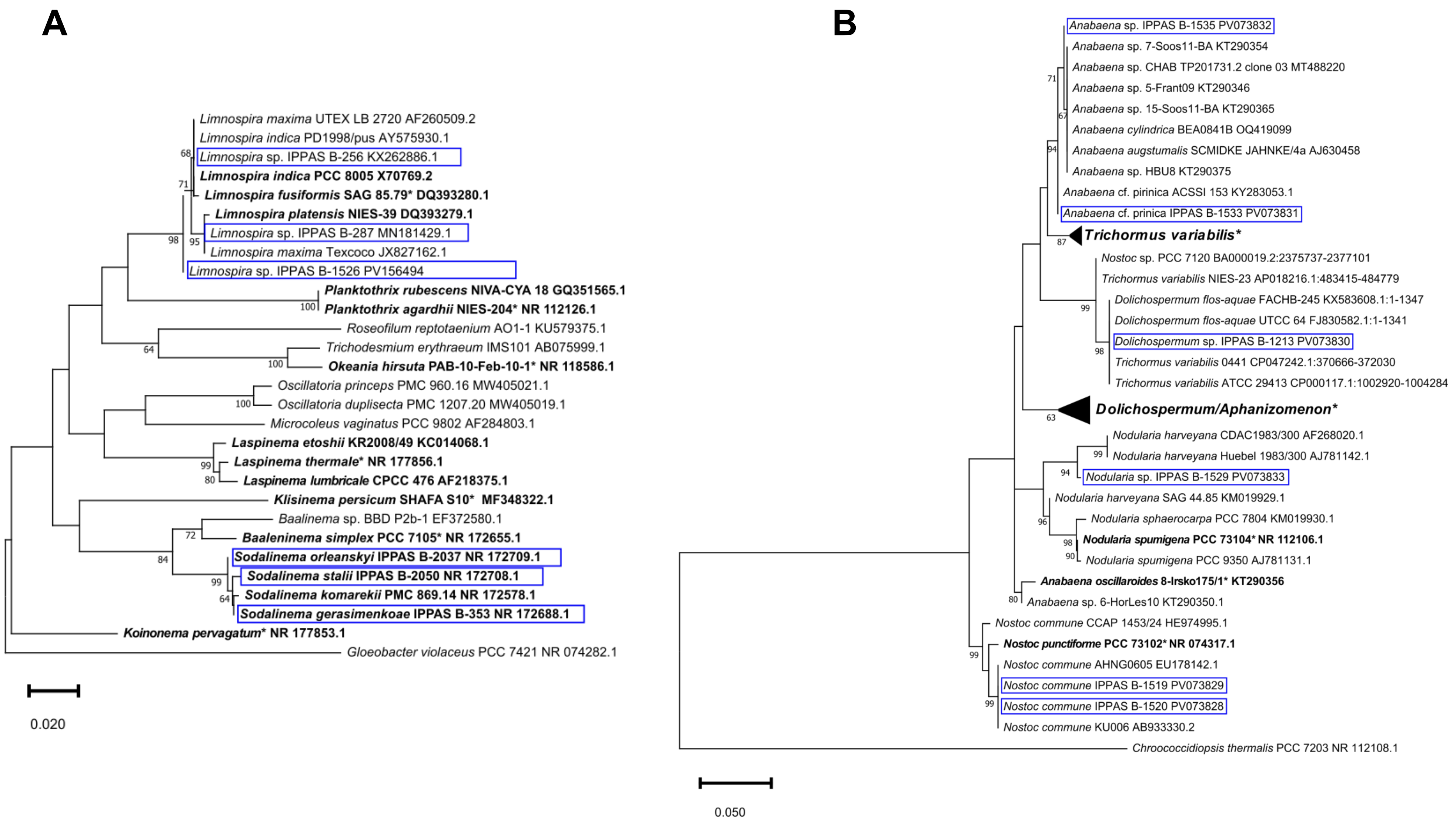

Figure S1-2. Maximum likelihood phylogenetic trees based on partrial 16S rRNA gene sequences showing the positions of the studied strains among reference strains (highlighted in bold) and type species of genera (marked with asterisk) belonging to orders *Oscillatoriales* (A) and *Nostocales* (B). Studied herein strains are marked with blue frame. The numbers above branches indicate statistical significance of the branching order as determined by bootstrap analysis of 1000 alternative trees (only values higher than 60% are shown). There were a total of 565 (A) and 521 (B) positions in the final datasets with 29 (A) and 48 (B) nucleotide sequences involved. Evolutionary analyses were conducted in MEGA X (Kumar S., Stecher G., Li M., Knyaz C., and Tamura K. (2018). MEGA X: Molecular Evolutionary Genetics Analysis across computing platforms. Molecular Biology and Evolution 35:1547-1549).
