# Supplementary material for: Strain-Specific Features of Primary Metabolome Characteristic for Extremotolerant/Extremophilic Cyanobacteria Under Long-Term Storage
Source: Int J Mol Sci. 2025 Feb 28;26(5):2201. doi: 10.3390/ijms26052201 (PMC11900582; doi:10.3390/ijms26052201)
Supplement: Supplementary file 1 [file ijms-26-02201-s001.zip › Supplementary_Information_S2 (Tables and Figures with detailed information related the manuscript).pdf]

Strain-Specific Features of Primary Metabolome Characteristic for Extremotolerant/Extremophilic Cyanobacteria Under Long-Term Storage

Tatiana Bilova[1,2*], Nikita Golushko[1,2], Nadezhda Frolova[1], Alena Soboleva[1], Svetlana Silinskaia[1], Anna Khakulova[3], Anastasia Orlova[1], Maria Sinetova[4], Dmitry Los[4] and Andrej Frolov[1*]

Supplementary Information S2

[1] Laboratory of Analytical Biochemistry and Biotechnology, K.A. Timiryazev Institute of Plant Physiology RAS, 127276 Moscow, Russia;

[2] Department of Plant Physiology and Biochemistry, St. Petersburg State University, 199034 St. Petersburg, Russia;

[3] Chemical Analysis and Materials Research Core Facility Center, Research Park, St. Petersburg State University, 199034 St. Petersburg, Russia;

[4] Laboratory of Intracellular Regulation, K.A. Timiryazev Institute of Plant Physiology RAS, 127276 Moscow, Russia;

* Correspondence: t.bilova@spbu.ru (T.B.); frolov@ifr.moscow (A.F.)

Directory

Tables…………………………………………………………………………………………4

Table S2-1 Thermostable primary metabolites detected by GC-MS analysis in the twelve strains of extremophilic cyanobacteria. 4

Table S2-2 The conditions of ion pair-reversed phase ultrahigh performance liquid chromatographic (IP-RP-UHPLC) separation and the settings for electrospray ionization-triple quadrupole-tandem mass spectrometry (ESI-QqQ-MS/MS) used for the analysis of anionic primary thermally labile metabolites from extremophilic cyanobacteria with IP-RP-UHPLC- ESI-QqQ-MS/MS..11

Table S2-3 Patterns of metabolites (identified and non-identified) detected by GC-MS and LC-MS methods in indicated desiccation-tolerant cyanobacteria strains and showing significant differences in relative content compared to the average content of these metabolites in all extremophilic strains studied. 18

Table S2–4 Patterns of identified and non-identified polar metabolites showing significant differences in relative content in extreme temperature tolerant cyanobacterial strains compared to the average content of these metabolites in all extremophilic strains studied..19

Table S2–5 Patterns of identified and non-identified polar metabolites showing significant differences in relative content in haloalkaliphilic, natronophilic cyanobacterial strains B-2037, B-353, B-2050 and B-1526 compared to the average content of these metabolites in all extremophilic strains studied. 21

Table S2-6 Patterns of identified and non-identified polar metabolites showing significant differences in relative content in haloalkaliphilic, natronophilic cyanobacterial strains B-1529, B-256 and B-287 compared to the average content of these metabolites in all extremophilic strains studied. 23

Table S2-7 Patterns of identified and non-identified polar metabolites showing significant differences in relative content in diazotrophic heterocystous compared to the non-heterocystous extremophilic cyanobacterial strains studied. 28

Table S2-8 The most important metabolic pathways for the haloalkalophilic and natronophilic cyanobacterial strains with appeared considerable intergroup variability. 30

Table S2-9 Gas chromatographic separation conditions and electron ionization-quadrupole-mass spectrometry settings for analysis of extremophilic cyanobacteria primary polar thermally stabile metabolites. 31

Table S2-10 Kovats retention indicesof linear alkanes...32

Figures……………………………………………………………………………….. 33

Figure S2-1 PCA loadings plot of the studied extremophilic cyanobacterial strains.. 33

Figure S2-2 Comprehensive statistical analysis of polar metabolite profiles of the extremophilic cyanobacteria strain *Nostoc commune* B-1520 35

Figure S2-3 Comprehensive statistical analysis of polar metabolite profiles of the extremophilic cyanobacteria strain *Nostoc commune* B-1519 37

Figure S2-4 Comprehensive statistical analysis of polar metabolite profiles of the extremophilic cyanobacteria strain *Dolichospermum* sp. B-1213 39

Figure S2-5 Comprehensive statistical analysis of polar metabolite profiles of the extremophilic cyanobacteria strain *Anabaena* cf. pirinica B-1533 42

Figure S2-6 Comprehensive statistical analysis of polar metabolite profiles of the extremophilic cyanobacteria strain *Anabaena sp.* B-1535 44

Figure S2-7 Comprehensive statistical analysis of polar metabolite profiles of the extremophilic cyanobacteria strain *Sodalinema orleanskyi* B-2037 .. ……………………………………………………46

Figure S2-8 Comprehensive statistical analysis of polar metabolite profiles of the extremophilic cyanobacteria strain *Sodalinema gerasimenkoae* B-353 48

Figure S2-9 Comprehensive statistical analysis of polar metabolite profiles of the extremophilic cyanobacteria strain *Sodalinema stalii* B-2050 50

Figure S2-10 Comprehensive statistical analysis of polar metabolite profiles of the extremophilic cyanobacteria strain *Limnospira* sp. B-1526 …………………………………………………………52

Figure S2-11 Comprehensive statistical analysis of polar metabolite profiles of the extremophilic cyanobacteria strain *Nodularia* sp. B-1529 56

Figure S2-12 Comprehensive statistical analysis of polar metabolite profiles of the extremophilic cyanobacteria strain *Limnospira* sp. B-287 …………………………………. ………………………59

Figure S2-13 Statistical analysis of polar metabolite profiles of the haloalkaliphilic and natronophilic cyanobacteria strain *Limnospira* sp. B-256. 62

Figure S2-14 Pathway analysis accomplished for haloalkali- and natronophilic cyanobacteria strains B-1529, B-287 and B-256...63

Literature...………63

Tables

Table S2-1 Thermostable primary metabolites detected by GC-MS analysis in the twelve strains of extremophilic cyanobacteria.

#	Metabolite features[a]	Derivatization group[b]	t_R exp[c]	RI[d]	*m/z* quant[e]	Annotation[f]
1	RI1016 Unknown		7.56	1016.5	156	
2	Propylene glycol	2TMS	7.63	1019.2	117	NIST 840
3	RI1022 Unknown		7.69	1021.9	184	
4	Dimethylglycine	1TMS	7.9	1030.8	58	NIST 935
5	RI1032 Unknown		7.92	1031.6	160	
6	Ethanolamine	2TMS	8	1035.2	102	NIST 777
7	2-Hydroxypyridine	1TMS	8.37	1050.6	152	GMD 910
8	RI1052 Unknown		8.41	1052.2	148	
9	*D*-Lactic acid		8.7	1064.5	191	NIST 876
10	*L*-Lactic acid	2TMS	9.11	1081.9	191	ihASL
11	RI1087 Unknown		9.22	1086.6	173	
12	Oxalic acid	2TMS	9.24	1087.5	190	NIST 709
13	Glycolic acid	2TMS	9.45	1096.2	177	ihASL
14	RI1103 Unknown		9.62	1103.5	186	
15	Alanine derivate		9.65	1104.8	116	ihASL
16	Valine	1TMS	9.71	1107.3	72	NIST 935
17	Alanine 1L		9.77	1110.2	116	ihASL
18	Hydroxylamine	3TMS	9.95	1170.6	249	NIST 869
19	Alanine 2H	2TMS	9.99	1119.2	116	ihASL
20	Glycine	2TMS	10.39	1136.2	102	ihASL
21	Oxalic acid	2TMS	10.6	1145.2	190	ihASL
22	RI1158 Unknown		10.91	1158.3	248	
23	RI1165 Unknown		11.08	1165	205	
24	Leucine	1TMS	11.11	1166.6	86	ihASL
25	3-Hydroxybutyric acid	2TMS	11.17	1169.3	191	NIST 944
26	RI1182 Unsaturated alcohol		11.47	1181.7	137	NIST 641
27	Methyl-phosphate	2TMS	11.56	1185.9	241	NIST 789
28	Isoleucine	1TMS	11.57	1186.1	86	ihASL
29	RI1188 Unknown		11.63	1188.7	241	
30	RI1214 Unknown		12.26	1214.2	178	
31	RI1217 Unknown		12.32	1216.9	230	
32	Valine	2TMS	12.36	1218.4	144	ihASL
33	RI1219 Unknown		12.38	1219.1	261	
34	2-Hydroxyhexanoic acid	2TMS	12.59	1227.8	159	NIST 783
35	Serine	2TMS	13.29	1255.7	116	ihASL
36	Ethanolamine	3TMS	13.41	1260.2	174	NIST 873
37	Phosphoric acid	3TMS	13.59	1267.7	299	NIST 943
38	Glycerol	3TMS	13.65	1270.1	205	ihASL
39	Isoleucine	2TMS	14.06	1286.4	158	ihASL
40	Threonine	2TMS	14.1	1287.8	219	ihASL
41	Glycine	3TMS	14.31	1296.7	174	ihASL
42	RI1299 Unknown		14.39	1299.4	113	
43	Succinic acid	2TMS	14.61	1308.3	247	ihASL
44	RI1320 Unknown		14.9	1320	285	
45	Glyceric acid	3TMS	14.94	1321.4	189	ihASL
46	Uracil	2TMS	15.1	1327.9	241	NIST 880
47	RI1329 Unknown		15.13	1329	180	
48	Itaconic acid	2TMS	15.24	1333.2	259	ihASL
49	Fumaric acid	2TMS	15.42	1340.7	245	
50	RI1342 Unknown		15.45	1342	292	
51	Alanine	3TMS	15.46	1342.1	188	GMD 847
52	RI1342 C4-carboxylic acid	3TMS	15.46	1342.2	292	
53	Serine	3TMS	15.53	1345.1	204	ihASL
54	RI1349 Unknown		15.62	1348.6	221	
55	RI1352 Unknown		15.69	1351.5	293	

56	Nonanoic acid	1TMS	15.7	1352	215	NIST 648
57	RI1357 Unknown		15.84	1357.5	158	
58	Threonine	3TMS	16.1	1367.8	218	ihASL
59	RI1376 Unknown		16.29	1375.6	191	
60	RI1383 Amino acid		16.49	1383.4	160	
61	Hydroquinone	2TMS	16.51	1384.5	239	NIST 742
62	2,4-Dihydroxybutanoic acid	3TMS	16.75	1393.9	219	NIST 815
63	RI1398 Unknown		16.87	1398.4	229	
64	RI1400 Unknown		16.9	1399.8	99	
65	Aspartic acid	2TMS	16.94	1401.6	160	ihASL
66	β-Alanine	3TMS	16.97	1403.1	248	ihASL
67	3-Methylglutaconic acid peak 1	2TMS	17.27	1417.7	229	NIST 668
68	3-Methylglutaconic acid peak 2	2TMS	17.36	1422.3	273	NIST 616
69	Homoserine	3TMS	17.45	1426.9	218	ihASL
70	RI1443 Unknown		17.79	1443.3	255	
71	RI1450 Unknown		17.92	1449.8	231	
72	RI1451 Unknown		17.97	1451.8	281	
73	RI1461 Unknown		18.15	1460.7	231	
74	Malate	3TMS	18.33	1468.3	233	ihASL
75	RI1477 phosphate conjugated compound		18.49	1477.3	327	
76	*meso*-Erythritol	4TMS	18.54	1479.8	217	ihASL
77	Pyroglutamic acid	1TMS	18.69	1487	84	NIST 720
78	Salicylic acid	2TMS	18.71	1487.9	267	ihASL
79	RI1489 C4-polyol		18.73	1489.1	205	
80	RI1491 Unknown		18.77	1491.2	283	
81	Aspartic acid	3TMS	18.92	1498.2	232	ihASL
82	Pyroglutamic acid	2TMS	18.93	1498.7	156	ihASL
83	RI1501 Unknown		18.97	1500.8	201	
84	γ-Aminobutyric acid	3TMS	19.07	1506.1	174	ihASL
85	Glutamic acid	2TMS	19.21	1513.4	174	GMD 832
86	RI1516 Phenolic compound		19.26	1516.5	263	
87	RI1519 Unknown		19.31	1519	202	
88	*D*-Erythronic acid	4TMS	19.38	1522.7	292	ihASL
89	1-Deoxypentitol	4TMS	19.4	1523.7	307	NIST 836
90	Phenylalanine	1TMS	19.5	1529	146	ihASL
91	RI1532 Unknown		19.56	1532	219	
92	RI1542 C4-5-sugar derived acid		19.74	1541.8	292	
93	RI1550 Unknown		19.91	1550.5	330	
94	1-Dodecanol	1TMS	19.98	1554.3	243	NIST 798
95	*alpha*-Hydroxyglutaric acid	3TMS	20.07	1559.3	247	NIST 809
96	RI1552 Monosaccharide (C5-furanose)	4TMS	20.31	1571.7	217	
97	RI1577 Unknown		20.41	1577.3	233	
98	1,2,4-Benzenetriol	3TMS	20.51	1582.6	239	NIST 817
99	RI1589 Unknown		20.64	1589.4	171	
100	2-Ketoglutaric acid	3TMS	20.69	1591.8	347	NIST 500
101	Glutamic acid	3TMS	20.89	1602.6	246	ihASL
102	Phenylalanine	2TMS	20.96	1606.7	218	ihASL
103	RI1612 Unknown		21.05	1611.6	328	
104	4-Hydroxyphenylacetic acid	2TMS	21.29	1625.1	296	NIST 829
105	Xylose 1L	1MEOX, 4TMS	21.38	1629.7	307	ihASL
106	Xylose 2H	1MEOX, 4TMS	21.56	1639.9	307	ihASL
107	Dodecanoic acid	1TMS	21.59	1641.6	257	NIST 773
108	RI1643 Unknown		21.63	1643.8	245	
109	Ribulose&Xylulose	1MEOX, 4TMS	21.94	1661.1	205	ihASL
110	Ribose	1MEOX, 4TMS	21.97	1662.6	307	ihASL
111	RI1680 C5-polyol		22.28	1680.4	307	
112	RI1687 Unknown		22.4	1686.9	345	
113	Arabitol	5TMS	22.72	1704.8	307	ihASL
114	Ribitol	5TMS	22.81	1709.9	319	ihASL
115	Ornithine	3TMS	23.28	1737.2	174	ihASL
116	Glycerol 3-phosphate	4TMS	23.54	1751.8	357	ihASL
117	RI1756 C5-sugar		23.6	1755.6	217	
118	RI1762 Unknown		23.72	1762.4	215	
119	RI1768 Phenolic compound		23.82	1768.2	299	

120	RI1775 Unknown		23.94	1775	273	
121	RI1755 C5-sugar derivate		24	1755.1	253	
122	Terephthalic acid	2TMS	24.04	1780.9	295	NIST 862
123	Propanoic acid	4TMS	24.32	1796.9	357	NIST 837
124	3-Phosphoglyceric acid	3TMS	24.34	1798.2	299	ihASL
125	RI1803 Unknown		24.42	1803.1	142	
126	Citric acid	4TMS	24.51	1808.1	273	ihASL
127	Isocitric acid	4TMS	24.56	1811.1	245	NIST 853
128	RI1819 Sugar		24.69	1819	217	
129	RI1827 Unknown		24.83	1827.3	230	
130	RI1832 Unknown		25.01	1838.2	260	
131	Lysine	3TMS	25.04	1840.2	174	ihASL
132	Tetradecanoic acid	1TMS	25.07	1842	285	NIST 843
133	RI1845 C5-C6-monosaccharide		25.12	1845	319	
134	Adenine	2TMS	25.37	1859.9	264	ihASL
135	Fructose 1H	1MEOX, 5TMS	25.39	1861.3	307	ihASL
136	Fructose 2L	1MEOX, 5TMS	25.57	1871.8	307	ihASL
137	Mannose 1H	1MEOX, 5TMS	25.62	1874.9	319	ihASL
138	Tyrosine	2TMS	25.66	1877.4	179	NIST 894
139	Galactose 1H	1MEOX, 5TMS	25.69	1879.5	319	ihASL
140	Glucopyranose	5TMS	25.73	1881.8	204	GMD 772
141	Glucose 1H	1MEOX, 5TMS	25.82	1887	319	ihASL
142	RI1893 Unknown		25.91	1892.6	116	
143	RI1896 Sugar derived acid		25.97	1895.9	217	
144	RI1897 Unknown		25.98	1896.5	320	
145	RI1902 Sugar derived acid		26.07	1902.2	333	
146	RI1903 Fatty acid	1TMS	26.07	1902.1	299	NIST 902
147	RI1904 Unknown		26.1	1903.9	217	
148	Glucose 2L	1MEOX, 5TMS	26.11	1904.7	319	ihASL
149	RI1904 Sugar derivative		26.11	1904.4	161	
150	Lysine	4TMS	26.21	1910.6	174	ihASL
151	RI1911 Unknown		26.21	1911	217	
152	RI1915 Saccharide		26.29	1915.8	361	
153	Methylcitric acid	4TMS	26.31	1917.2	287	NIST 663
154	Mannitol	6TMS	26.34	1919.3	319	ihASL
155	Methyl palmitate		26.38	1921.9	143	NIST 939
156	RI1924 Sugar derived acid		26.41	1923.6	333	
157	Sorbitol	6TMS	26.47	1927	319	ihASL
158	Tyrosine	3TMS	26.5	1929.4	218	NIST 885
159	RI1941 Fatty acid		26.68	1940.9	299	
160	*n*-Pentadecanoic acid		26.71	1942.6	243	
161	RI1948 Unknown		26.81	1948.3	57	
162	RI1956 Unknown		26.92	1955.6	299	
163	RI1960 C6-sugar		26.99	1959.7	217	
164	RI1966 Unknown		27.08	1965.7	327	
165	RI1970 Saccharide (pyranose)		27.24	1975.5	204	
166	RI1976 Sugar phosphate		27.25	1975.9	299	
167	RI1977 Unknown		27.26	1976.8	226	
168	RI1979 Unknown		27.31	1979.5	375	
169	Gluconic acid	6TMS	27.49	1991	333	ihASL
170	Pterin	2TMS	27.52	1993.2	307	NIST 566
171	RI1996 C6-sugar		27.56	1995.6	204	
172	Glucaric acid	6TMS	27.62	1999.3	333	ihASL
173	RI2006 Fatty acid		27.72	2005.6	309	
174	RI2009 Unknown		27.77	2008.8	155	
175	RI2014 phosphate-conjugated compound		27.85	2014.2	243	
176	*trans*-9-Hexadecenoic acid	1TMS	27.86	2014.9	311	NIST 849
177	RI2019 Inositol isomer		27.92	2018.7	318	
178	RI2019 Sugar derived acid		27.92	2018.7	333	
179	*cis*-9-Hexadecenoic acid	1TMS	27.94	2018.9	311	NIST 952
180	Mucic acid	6TMS	28.15	2033.6	333	ihASL
181	RI2035 Unknown		28.17	2034.9	417	
182	Palmitic acid	1TMS	28.27	2041.4	313	ihASL

183	RI2050 Unknown		28.41	2050.2	282	
184	RI2056 Unknown		28.48	2056	278	
185	RI2060 C6-pyranose		28.56	2060.5	204	
186	RI2064 C6-pyranose		28.61	2063.7	204	
187	RI2065 Unknown		28.63	2064.9	285	
188	RI2067 C6-pyranose		28.66	2067.5	204	
189	*N*-acetyl-glucosamine 2H	1MEOX, 4TMS	28.73	2071.7	333	ihASL
190	*γ*-Linolenic acid, methyl ester		28.79	2075.6	194	NIST 883
191	RI2080 Unknown		28.85	2079.7	322	
192	*myo*-Inositol	6TMS	28.89	2082	305	ihASL
193	RI2086 Unknown		28.95	2086	116	
194	RI2092 C6-pyranose		29.04	2092.3	204	
195	Linoleic acid, methyl ester		29.05	2092.4	294	NIST 923
196	RI2093 Unknown		29.06	2093.3	298	
197	RI2102 C5-C6-Sugar phosphate		29.18	2102	315	
198	Similar to Heptadecanoic acid	1TMS	29.21	2103.4	327	NIST 854
199	RI2105 C5-C6-Sugar phosphate		29.24	2105.4	315	
200	*cis*-10-Heptadecenoic acid	1TMS	29.4	2116	325	NIST 679
201	RI2124 Sugar		29.51	2123.6	319	
202	Stearic acid methyl ester		29.57	2127.5	298	NIST 768
203	RI2129 Unknown		29.6	2129.3	156	
204	RI2134 Unknown		29.67	2134.1	256	
205	RI2136 C5-6-sugar phosphate		29.7	2136	299	
206	RI2140 C6-C7-Sugar		29.75	2139.6	204	
207	Heptadecanoic acid	1TMS	29.79	2142.1	327	NIST 615
208	RI2145 C6-C7-Sugar		29.84	2145.2	217	
209	RI2150 Phosphoric compound		29.92	2150.9	299	
210	RI2160 Amine or amino acid		30.06	2160.5	174	
211	RI2162 Fatty acid		30.08	2161.6	325	
212	RI2163 Sugar derivate		30.1	2163.3	217	
213	Phytol	1TMS	30.2	2169.9	143	NIST 831
214	Isoxanthopterin	3TMS	30.35	2179.6	380	NIST 833
215	RI2183 Unknown		30.4	2182.7	167	
216	*γ*-linolenic acid ((*6Z,9Z,12Z*)-Octadecatrienoic acid)	1TMS	30.45	2186.4	145	
217	Glycerophosphoglycerol	5TMS	30.52	2191	357	
218	RI2199 C6-C7-Sugar		30.64	2199.2	361	
219	RI2205 C6-C7-Sugar		30.72	2204.8	217	
220	Linoleic acid	1TMS	30.75	2206.7	337	NIST 934
221	Oleic acid	1TMS	30.83	2213	339	NIST 840
222	*trans*-9-Octadecenoic acid	1TMS	30.94	2220.4	339	NIST 861
223	Stearic acid	1TMS	31.23	2241.3	314	ihASL
224	RI2247 C5-C6-Sugar phosphate		31.32	2247.3	315	
225	Glucosylglycerol peak 1	6TMS	31.43	2255.5	204	NIST 882
226	RI2274 Unknown		31.69	2274	233	
227	RI2280 Fatty acid		31.78	2280.4	367	
228	RI2281 Fatty acid		31.8	2281.4	353	
229	RI2282 Unknown		31.81	2282.2	225	
230	RI2287 C5-C6-Sugar phosphate		31.88	2287.3	299	
231	Fructose 6-phosphate	1MEOX, 6TMS	32.1	2303	315	ihASL
232	RI2304 Sugar		32.11	2303.7	204	
233	Glucosylglycerol peak 2	6TMS	32.22	2309.9	204	NIST 865
234	Glucose-6-phosphate 1H	1MEOX, 6TMS	32.26	2314.7	387	ihASL
235	*cis*-Nonadecenoic acid	1TMS	32.44	2326	353	NIST 784
236	Glucose-6-phosphate 2L	1MEOX, 6TMS	32.51	2332	387	ihASL
237	RI2339 Unknown		32.62	2338.7	339	
238	RI2346 C6-Sugar phosphate		32.7	2346.1	387	
239	Glucosylglycerol peak 3	6TMS	32.89	2359.9	204	NIST 865
240	RI2359 Unknown		32.9	2359.2	247	
241	RI2366 Unknown		32.98	2366.2	241	
242	RI2372 Sugar derived acid		33.07	2371.5	292	
243	RI2374 C6-Sugar phosphate		33.09	2373.9	387	
244	RI2378 Unknown		33.16	2378.7	329	
245	RI2380 Unknown		33.18	2380	303	

246	RI2384 Unknown		33.24	2384	131	
247	RI2386 C6-Sugar phosphate		33.26	2385.5	387	
248	RI2388 Unknown		33.3	2388.2	329	
250	*myo*-Inositol phosphate	7TMS	33.51	2403.4	318	NIST 901
251	RI2406 Unknown		33.54	2405.9	125	
252	RI2408 Unknown		33.57	2408.3	225	
253	RI2146 Sugar phosphate		33.67	2416.1	299	
254	Nonadecan-1-ol	1TMS	33.97	2430.9	341	NIST 726
255	RI2441 Unknown		34	2440.8	131	
256	RI2447 Disaccharide		34.09	2447	217	
257	RI2448 C6-7 Sugar phosphate		34.1	2448	315	
258	RI2449 Unknown		34.12	2449.8	271	
259	RI2455 Sugar (pyranose)-derivate		34.19	2455	361	
260	Inositol phosphate peak 2		34.25	2459.2	318	GMD 772
261	RI2480 Sugar derivate		34.53	2480.5	173	
262	RI2484 Sugar-derivate		34.57	2483.7	204	
263	RI2498 Sugar (pyranose)-derivate		34.77	2498.4	361	
264	RI2517 Unknown		35.01	2517.3	204	
265	RI2524 Unknown peak 1		35.1	2524.2	375	
266	RI2531 Unknown peak 2		35.18	2531.2	375	
267	Mono(2-ethylhexyl) phthalate		35.23	2535.2	149	NIST 911
268	RI2536 Saccharide		35.25	2536.2	204	
269	RI2542 C6-7 Sugar phosphate		35.32	2542.1	387	
270	RI2549 Unknown		35.41	2549	219	
271	RI2550 Sugar-derivate		35.43	2550.5	204	
272	RI2554 C6-7 Sugar phosphate		35.48	2554.2	387	
273	1-Monopalmitoylglycerol	2TMS	35.86	2584	371	NIST 735
274	RI2590 Unknown		35.94	2590.3	283	
275	RI2600 Disaccharide		36.07	2600.2	361	
276	RI2617 Disaccharide (with pyranose)-methyl derivate		36.3	2619.6	303	
277	RI2619 Disaccharide (with pyranose)-derivate		36.3	2619	259	
278	Sucrose	8TMS	36.42	2629.2	361	ihASL
279	RI2637 Unknown		36.52	2637	259	
280	RI2657 Unknown		36.77	2657.3	303	
281	RI2663 Sugar-derivate		36.84	2662.9	319	
282	RI2670 Unknown		36.92	2670	361	
283	RI2692 Unknown		37.19	2691.8	174	
284	RI2694 Disaccharide (with pyranose)-derivate		37.22	2694.5	303	
285	RI2700 Disaccharide (with pyranose)-derivate		37.3	2700.2	303	
286	Maltose 1H	1MEOX, 8TMS	37.64	2728.5	361	ihASL
287	α,α-Trehalose	8TMS	37.7	2734.5	361	ihASL
288	RI2739 Sugar-derivate		37.75	2738.6	204	
289	Maltose 2L	1MEOX, 8TMS	37.96	2755.9	361	ihASL
290	RI2767 Disaccharide		38.09	2767.1	361	
291	Guanosine	5TMS	38.11	2768.4	324	NIST 596
292	RI2772 Disaccharide		38.14	2771.7	204	
293	RI2783 Disaccharide		38.28	2783.1	361	
294	5'-Methylthioadenosine	3TMS	38.31	2784.9	236	NIST 650
295	RI2788 Unknown		38.34	2788.3	311	
296	RI2799 Unknown		38.47	2798.8	217	
297	RI2820 Disaccharide		38.71	2819.7	361	
298	Maltitol	9TMS	38.83	2830.9	361	ihASL
299	RI2845 Disaccharide		39	2845.4	361	
300	RI2860 Unknown		39.17	2859.9	351	
301	Isomaltose	1MEOX, 8TMS	39.23	2865.6	361	ihASL
302	RI2871 Unknown		39.29	2870.6	204	
303	RI2882 Unknown		39.42	2882.1	160	
304	RI2926 Unknown		39.92	2926.1	204	
305	RI2936 Unknown		40.03	2935.6	87	
306	RI2953 Unknown		40.22	2953.4	395	

307	Galactinol	9TMS	40.48	2977.1	204	ihASL
308	RI2992 Unknown (phenyl group)		40.66	2992.3	224	
309	RI3124 Disaccharide-derivate		40.75	3001	361	
310	RI3027 Unknown		41.04	3027.3	204	
311	RI304 Disaccharide derived alcohol		41.21	3043.6	204	
312	RI3047 Disaccharide-derivate		41.25	3047	204	
313	RI3056 Disaccharide-derivate		41.35	3056.5	361	
314	RI3061 Disaccharide-derivate		41.4	3061.5	204	
315	Adenosine	5TMS	41.4	3060.7	315	NIST 682
316	RI3067 Unknown		41.46	3067.1	261	
317	RI3081 Unknown		41.62	3081.4	173	
318	RI3124 Disaccharide-derivate		42.07	3124.5	204	
319	RI3133 Disaccharide-derivate		42.16	3133.4	204	
320	RI3154 Disaccharide-derivate		42.38	3154.4	361	
321	RI3172 Disaccharide-derivate		42.57	3172.3	361	
322	RI3190 Disaccharide-derivate		42.75	3190	204	
323	RI3197 Disaccharide-derivate		42.83	3197.3	204	
324	RI3201 Unknown		42.88	3201.7	291	
325	RI3216 Unknown		43.02	3215.9	409	
326	RI3240 Unknown		43.27	3240.4	295	
327	RI3268 Di- or trisaccharide-derivate		43.55	3268.4	361	
328	RI3315 Di- or trisaccharide-derivate		44.03	3315.7	204	
329	RI3331 Di- or trisaccharide-derivate		44.17	3331	204	
330	RI3349 Di- or trisaccharide-derivate		44.36	3349.4	361	
331	RI3351 Unknown		44.37	3351	219	
332	RI3384 Trisaccharide		44.7	3384.5	361	
333	RI3408 Trisaccharide		44.93	3407.6	204	
334	RI3418 Trisaccharide		45.02	3417.8	361	
335	RI3462 Trisaccharide (Erlose-like)	11TMS	45.45	3462.2	361	
336	RI3464 Glucuronide-conjugated		45.47	3464.5	204	
337	RI3502 Trisaccharide		45.83	3502.3	361	
338	RI3516 Trisaccharide		45.97	3515.6	204	
339	RI3528 Unknown		46.1	3528.4	361	
340	RI3535 Trisaccharide		46.17	3534.9	204	
341	RI3555 Trisaccharide		46.37	3555	204	
342	RI3567 Unknown		46.49	3566.5	385	
343	RI3574 Unknown		46.57	3573.9	647	
344	Dilauryl 3,3'-thiodipropionate		47.04	3618.1	329	NIST 779
345	RI3622 Unknown		47.09	3622	143	
346	RI3649 Trisaccharide derivative		47.4	3649.3	361	
347	RI3714 Unknown		48.17	3713.6	204	
348	RI3736 Trisaccharide derivative		48.46	3736.1	204	
349	RI3748 Unknown		48.62	3747.8	295	
350	RI3766 Unknown		48.86	3766.1	313	
351	RI3783 Trisaccharide derivative		49.09	3783.2	485	
352	RI3805 Oligosaccharide		49.37	3805.5	204	
353	RI3816 Oligosaccharide		49.56	3816.1	204	
354	RI3836 Unknown		49.87	3836	313	
355	RI3839 Unknown peak 1		49.93	3839.5	485	
356	RI3851 Unknown peak 2		50.11	3851.2	485	
357	RI3880 Oligosaccharide		50.56	3880	361	
358	RI3999 Oligosaccharide		52.71	3999.2	361	

[a]Metabolite features are arranged in order of increasing retention times (t_R).

Unidentified metabolites are labeled with the word *Unknown*, their annotation contains the retention index (RI) and relies on t_R and *m/z*. The name of metabolites annotated to a certain chemical class (without exact annotated structure) also begins with RI, followed by the name of the corresponding chemical class. Annotation to specific chemical classes was confirmed by the presence of characteristic signals (*m/z* values) specific for the corresponding chemical class (for example, sugar phosphates – *m/z* 387, 315 and 299, disaccharides – *m/z* 361, 303, 204) [1]. [b]The numbers and types of derivatization groups attached to the identified metabolites: TMS – trimethylsilyl group, MEOX – methyloxime group; [c]retention time of the

metabolite; [d]retention index of the metabolite; [e]the *m/z* value of the most characteristic ion in the electron ionization mass-spectrum (quantifier), from which the extracted ion chromatogram was reconstructed, and the peak area integration was accomplished at the given t_R ; [f]annotation of total ion current (TIC) peaks to specific analytes (also called as features) relied on search obtained RIs and electron ionization mass-spectrum against established reference mass-spectral libraries. The following libraries were used for the metabolite structural annotation – National Institute of Standards and Technology (NIST, using match factor), Golm Metabolome Database (GMD) and in-house Authentic Standard Library (IhASL).

Table S2-2 The conditions of ion pair-reversed phase ultrahigh performance liquid chromatographic (IP-RP-UHPLC) separation and the settings for electrospray ionization-triple quadrupole-tandem mass spectrometry (ESI-QqQ-MS/MS) used for the analysis of anionic primary thermally labile metabolites from extremophilic cyanobacteria with IP-RP-UHPLC- ESI-QqQ-MS/MS.

Chromatography

**ACQUITY Sample Manager (SM)**	
Injection mode	PartialLoop
Injection volume	5 µL
Weak wash solvent	0.3 mmol/L aq. ammonium formate
Weak wash volume	800 µL
Strong wash solvent	Acetonitrile
Strong wash volume	400 µL
Target sample temperature	4.0 C
Needle overfill flush	Automatic
**Column conditions**	
Separation column	EC 150/2 NUCLEOSHELL RP 18<br>(150 x 2 mm, particle size 2.7 µm)
Target column temperature	40.0 C
**ACQUITY Binary Solvent Manager (BSM)**	
Eluent A	10 mmol/L tributylamine (TBA) in water, pH 6.2 (adjusted by acetic acid)
Eluent B	Acetonitrile
Seal wash duration	5 min
Flow rate	0.4 mL/min
Elution program	2% eluent B isocratic - 2 min<br>gradient to 36% eluent B – 16 min

	gradient to 95% eluent B – 3 min
	95% eluent B isocratic – 1.5 min
	gradient to 2% eluent B – 0.1 min
	2% eluent B isocratic – 2.4 min (re-equilibration)

Mass spectrometry

**General**	
Mass analyzer type	triple quadrupole-linear ion trap (QqLIT, QTRAP, operated in QqQ mode)
Ion source	TurboIonSpray®
Experiment type	multiple reaction monitoring (MRM)
Operatinon mode	negative
Cycle time (s)	1.2
Pause between ranges (ms)	5.007
Settling time (s)	0
Duration	24 min
**Ion source settings**	
Nebulizer gas (psig)	60
Drying gas (psig)	70
Curtain gas (psig)	40
Ion spray voltage (kV)	-4.5
Ion source temperature (°C)	450
**MS/MS settings**	
Fragmentation mode	CAD
MS/MS experiment type	MRM

Collision gas	nitrogen
Collision gas pressure	3 psig (medium)
Entrance potential (V)	-10.0
Scheduled MRM	enabled
Scheduled MRM type	basic
MRM detection window (s)	500
Target scan time (s)	1
Dwell time	adjusted by scheduled MRM algorithm
Q1 resolution	unit
Q3 resolution	unit
Declustering potential (DP, V)	compound-specific (listed below)
Collision potential (CE, V)	compound-specific (listed below)
Exit potential (CXP, V)	compound-specific (listed below)

Analyte-specific settings

Analyte-specific combinations of Q1 and Q3 *m/z* ranges (transitions)							
**#**	**Analyte**	**t_R (min)**	**Q1[a] (*m/z*)**	**Q3[b] (*m/z*)**	**DP (V)**	**CE (V)**	**CXP (V)**
1	2-deoxy-*D*-ribose 5-phosphate	N/F	212.9	97.1	-40	-20	-19
2	3-[(carboxylatovinyl)oxy]benzoate	N/F	207.1	179.0	-240	-38	-13
3	5-amino-4-imidazolecarboxamide ribotide	N/F	337.1	78.9	-85	-50	-5
4	5-formamido-1-(5-phospho-D-ribosyl)imidazole-4-carboxamide	N/F	365.0	78.9	-40	-35	-10
5	5-formyl-tetrahydrofolate	N/F	472.2	315.1	-40	-35	-10
6	5-methyl-tetrahydrofolate	N/F	458.2	329.1	-40	-35	-10
7	5′-phosphoribosyl-*N*-formylglycinamide	N/F	313.0	78.9	-40	-35	-10
8	5'-phosphoribosyl-5-aminoimidazole	N/F	294.0	78.9	-40	-43	-10
9	allantoic acid	N/F	175.0	132.0	-35	-32	-12
10	beta-nicotinamide mononucleotide	N/F	334.0	78.9	-25	-16	-13
11	carboxyaminoimidazole ribotide	N/F	338.0	78.9	-40	-35	-10
12	chorismate	N/F	225.0	179.0	-35	-25	-10
13	cytidine-5'-diphosphate choline	N/F	487.0	428.0	-10	-20	-23
14	glycineamide ribonucleotide	N/F	285.0	78.9	-40	-35	-10
15	nicotinamide	N/F	121.0	76.9	-40	-16	-9
16	nicotinamide mononucleotide	N/F	333.0	78.9	-50	-30	-13
17	nicotinamide riboside	N/F	253.1	121.0	-40	-35	-10
18	phosphoribosylamine	N/F	227.0	78.9	-40	-35	-10
19	riboflavin-5′-phosphate	N/F	455.1	97.0	-35	-25	-10

20	succinylaminoimidazole-carboxamide ribotide	N/F	453.1	78.9	-40	-35	-10
21	tetrahydrofolate	N/F	444.2	176.1	-40	-35	-10
22	histidine	N/F	154.1	93.0	-40	-24	-3
23	**arginine**	0.6	173.1	131.0	-50	-18	-7
24	**glutamine**	0.6	145.1	108.9	-30	-18	-5
25	ornithine	N/F	131.1	82.9	-60	-20	-5
26	proline	N/F	114.1	86.0	-55	-18	-3
27	4-aminobutanoic acid	N/F	102.1	84.0	-35	-14	-7
28	alanine	N/F	88.0	41.9	-20	-20	-13
29	**allantoin**	0.7	157.0	97.2	-60	-16	-1
30	asparagine	N/F	131.1	87.1	-75	-16	-11
31	**citrulline**	0.7	174.1	131.0	-35	-18	-7
32	creatine	N/F	130.1	88.1	-25	-14	-5
33	cysteine	N/F	120.0	79.8	-25	-32	-2
34	lysine	N/F	145.1	99.0	-65	-14	-5
35	cystine	N/F	239.3	120.0	-40	-32	-1
36	**dehydroascorbic acid**	0.8	173.0	127.0	-15	-18	-17
37	**glycine**	0.8	74.0	74.0	-36	-13	-3
38	**methionine**	0.8	148.0	47.0	-45	-24	-5
39	hexoses	N/F	179.1	89.0	-50	-12	-13
40	**S-adenosyl-*L*-homocysteine**	0.8	383.1	133.9	-80	-36	-7
41	**serine**	0.8	104.0	74.0	-20	-16	-3
42	**sucrose**	0.8	341.1	89.0	-240	-38	-13
43	**threonine**	0.8	118.1	73.9	-25	-18	-3
44	**uridine**	0.8	243.1	109.9	-65	-22	-5
45	valine	N/F	233.3	116.0	-25	-10	-5
46	valine	N/F	116.1	7.0	-25	-20	-5
47	**cytidine**	1.0	242.1	108.9	-70	-18	-5
48	leucine + isoleucine	N/F	261.3	130.2	-30	-10	-1
49	leucine + isoleucine	N/F	130.1	87.1	-25	-25	-10
50	**tyrosine**	1.0	180.1	118.9	-60	-24	-5
51	**guanosine**	1.4	282.1	149.9	-80	-26	-7
52	**2'-deoxyguanosine**	1.5	266.1	150.0	-115	-24	-3
53	**adenosine**	1.5	266.1	133.9	-70	-12	-1
54	**phenylalanine**	1.5	164.1	103.0	-55	-24	-5
55	**aspartic acid**	1.9	132.0	88.0	-40	-18	-13
56	**glucopyranonic acid**	1.9	193.0	113.0	-20	-16	-5
57	**galactopyranuronic acid**	2.0	193.0	113.0	-20	-16	-5
58	glyoxilic acid	N/F	73.0	45.0	-25	-25	-10
59	***D*-galactonic acid/ *D*-gluconic acid**	2.1	195.1	129.0	-50	-18	-9
60	**glucosamine 6-phosphate**	2.1	258.0	97.0	-45	-24	-5
61	**glutamic acid**	2.1	146.0	102.0	-80	-18	-9
62	**ribonic acid**	2.3	165.0	75.0	-45	-20	-35
63	glucosamine 1-phosphate	N/F	258.0	78.9	-55	-42	-1
64	2’-deoxyadenosine	N/F	250.1	134.0	-115	-26	-9
65	**glucolate**	2.5	75.0	47.0	-30	-14	-13
66	**shikimic acid**	2.5	173.0	92.9	-15	-20	-5
67	**3-dehydroxyshikimic acid**	2.7	171.0	127.0	-25	-16	-15
68	**quinic acid**	2.7	191.1	85.0	-50	-28	-13
69	**uric acid**	2.7	167.0	124.0	-45	-20	-7
70	ascorbic acid	N/F	175.0	115.0	-25	-25	-5
71	carbamoyl-alanine	N/F	131.0	87.9	-10	-14	-13
72	**chloride**	2.8	35.0	35.0	-50	-10	-10

73	**dihydroorotic acid**	3.3	157.0	112.7	-40	-10	-5
74	**tryptophan**	3.4	203.1	116.2	-50	-22	-7
75	**lactic acid**	3.8	89.0	42.9	-15	-12	-5
76	glutathione	N/F	306.1	143.0	-5	-26	-7
77	**phosphate**	4.0	96.9	78.9	-40	-18	-15
78	**cyclic guanosine monophosphate**	4.5	344.0	150.0	-70	-34	-11
79	**orotic acid**	4.8	155.0	110.7	-25	-12	-5
80	**pyruvic acid**	4.9	87.0	43.0	-30	-12	-1
81	nicotinamide adenine dinucleotide	N/F	662.1	540.1	-45	-22	-15
82	**glucose 6-phosphate**	5.4	259.1	97.0	-65	-18	-13
83	**glyceraldehyde 3-phosphate**	5.4	169.0	97.0	-30	-12	-5
84	**fructose 6-phosphate**	5.5	259.0	96.9	-30	-20	-11
85	**2-keto-3-deoxy-6-phosphogluconate**	5.8	257.0	97.0	-30	-20	-9
86	**erythrose 4-phosphate**	5.9	199.0	96.8	-40	-12	-5
87	**adenosine 2',3'-cyclic mono-phosphate**	6.2	328.0	134.0	-125	-36	-5
88	**ribulose-5-phosphate**	6.3	229.0	96.8	-35	-20	-5
89	**glucose-1-phosphate**	6.4	259.0	240.8	-30	-16	-15
90	**ribulose-5-phosphate/xylulose-5-phosphate**	6.5	229.0	96.8	-45	-18	-15
91	**mevalonic acid lactone**	6.6	147.1	59.1	-45	-20	-7
92	**sedoheptulose 7-phosphate**	6.6	289.0	97.0	-50	-22	-5
93	**2-C-methylerythritol 4-phosphate**	6.8	215.0	78.9	-40	-56	-9
94	**glycerophosphoric acid**	6.8	171.0	78.8	-45	-24	-1
95	**cytidine monophosphate**	7.0	322.2	79.0	-65	-68	-5
96	**nicotinic acid**	7.0	122.0	77.9	-55	-16	-13
97	**pantothenic acid**	7.0	218.1	88.1	-55	-18	-5
98	adenosine 3',5'-cyclic mono-phosphate	N/F	328.0	134.0	-125	-36	-5
99	**ribose-1-phosphate**	7.7	229.0	211.0	-50	-14	-3
100	**uridine monophosphate**	7.8	323.0	79.0	-65	-68	-5
101	**guanosine 5'-monophosphate**	8.1	362.1	78.9	-65	-66	-5
102	**inosinic acid**	8.3	347.0	134.8	-70	-38	-7
103	**2'-deoxyguanosine 5'-monophosphate**	8.4	346.1	78.8	-80	-42	-3
104	dihydroxyacetone phosphate	N/F	169.1	97.0	-35	-14	-11
105	thymidine-5'-phosphate	N/F	321.0	78.8	-65	-58	-3
106	**1-deoxy-D-xylulose 5-phosphate**	8.8	213.0	97.0	-50	-18	-1
107	**adenosine monophosphate**	8.8	346.1	78.8	-70	-52	-3
108	**glutathione disulfide**	8.9	611.1	306.1	-35	-34	-7
109	**2'-deoxyadenosine 5'-monophosphate**	9.1	330.1	195.0	-85	-22	-17
110	**digalacturonic acid**	9.5	369.1	175.0	-75	-18	-17
111	phosphocreatine	N/F	210.0	78.9	-35	-22	-1
112	**malate**	9.8	133.0	115.0	-20	-16	-5
113	**succinic acid**	9.9	117.0	73.0	-25	-16	-7
114	**3-hydroxypyruvate**	10.0	103.0	59.0	-30	-22	-7
115	**4-diphosphocytidyl-2-C-methyl-*D*-erythritol**	10.0	520.1	78.9	-120	-108	-9
116	**ureidosuccinic acid**	10.1	175.1	131.8	-25	-16	-7
117	sulfate	N/F	97.0	97.0	-40	-18	-15
118	**uridine-5'-diphosphate-glucose**	10.2	565.0	323.0	-125	-36	-11
119	**uridine-diphosphate-*N*-acetylglucosamine**	10.2	606.1	384.8	-175	-36	-25
120	**fumaric acid**	10.4	115.0	71.0	-5	-12	-13
121	**adenosine diphosphoribose**	10.5	558.1	346.0	-170	-34	-19
122	**oxaloacetic acid-1**	10.5	131.0	87.0	-35	-10	-17
123	oxaloacetic acid-2	N/F	131.0	43.0	-35	-18	-11
124	**adenosine diphosphate glucose**	10.6	588.1	345.9	-140	-32	-19
125	**α-ketoglutaric acid**	10.7	145.0	101	-10	-12	-13

126	pentanoates	N/F	101.1	101.1	-50	-10	-5
127	**2C-methyl-*D*-erythritol 2,4-cyclodiphosphate**	10.9	277.0	79.0	-45	-64	-37
128	(R)-5-phosphomevalonic acid	N/F	227.0	97.0	-30	-35	-10
129	**1,4-dihydronicotinamide adenine dinucleotide**	11.4	664.1	78.9	-100	-124	-1
130	folate	N/F	440.1	311.1	-40	-35	-10
131	**2-phosphoglyceric acid**	12.7	185.0	79.0	-25	-20	-35
132	**isopentenyl pyrophosphate**	12.8	245.0	78.9	-15	-44	-37
133	**2*P*-glycolate**	12.9	155.0	79.0	-15	-36	-35
134	**xanthosine-5'-phosphate**	12.9	363.0	151.1	-60	-36	-5
135	**guanosine-5'-diphosphate**	13.0	442.0	78.9	-85	-70	-3
136	6-phosphogluconic acid	N/F	275.0	79.0	-60	-66	-5
137	**flavin adenine dinucleotide**	13.1	784.1	79.0	-60	-130	-1
138	**uridine-5'-diphosphate**	13.1	403.0	78.8	-75	-68	-3
139	**cytidine-5'-diphosphate**	13.1	402.0	78.9	-65	-70	-5
140	**3-phosphoglyceric acid**	13.2	185.0	96.7	-30	-22	-7
141	**(2E)-4-hydroxy-3-methylbut-2-en-1-yl diphosphate**	13.3	261.0	79.0	-40	-52	-9
142	**aconitic acid**	13.3	173.0	128.7	-25	-10	-55
143	**adenosine-5'-diphosphate**	13.3	426.2	78.9	-75	-66	-3
144	**nicotinamide adenine dinucleotide phosphate**	13.4	743.1	620.0	-55	-22	-17
145	**thymidine-5'-diphosphate**	13.4	401.0	78.8	-70	-68	-3
146	**2'-deoxyadenosine-5'-diphosphate**	13.5	410.0	78.9	-60	-76	-3
147	trigalacturonic acid	N/F	545.1	369.0	-105	-24	-25
148	**phosphoenolpyruvic acid**	13.9	167.0	78.8	-20	-16	-9
149	**isocitric acid**	14.1	191.1	73.0	-45	-28	-31
150	**citric acid**	14.2	191.0	87.0	-35	-22	-15
151	**dimethylallylpyrophosphat**	14.2	245.0	78.9	-15	-44	-37
152	**cytidine 5'-triphosphate**	15.4	482.2	158.8	-85	-36	-9
153	**4-diphosphocytidyl-2-C-methyl-*D*-erythritol 2-phosphate**	15.5	600.0	78.9	-115	-126	-19
154	**desoxyadenosintriphosphat**	15.5	490.0	391.9	-90	-34	-25
155	**sedoheptulose 1,7-bisphosphate**	15.5	369.0	97.0	-35	-20	-27
156	**adenosine triphosphate**	15.6	506.2	158.8	-80	-38	-9
157	**fructose-1,6-diphosphate**	15.7	339.0	96.9	-35	-22	-11
158	ribulose-1,5-bisphosphate	N/F	309.0	97.0	-35	-20	-27
159	**adenylosuccinic acid**	15.8	462.3	133.9	-85	-62	-7
160	orotidine 5'-monophosphate	N/F	367.0	78.9	-50	-78	-1
161	mevalonate-5-diphosphate	N/F	307.0	78.9	-25	-35	-13
162	**guanosine-5'-triphosphate**	16.0	522.0	158.8	-90	-48	-9
163	**deoxythymidine 5'-triphosphate**	16.0	481.0	158.7	-80	-38	-9
164	**dihydronicotinamide adenine dinucleotide phosphate**	16.3	744.1	79.0	-40	-16	-9
165	**ADP-ribose-2`-phosphate**	16.5	638.0	426.0	-170	-34	-19
166	coenzyme A	N/F	766.1	407.9	-245	-50	-19
167	inositol triphosphate	N/F	419.0	320.8	-25	-28	-21
168	5-phosphoribosyl diphosphate	N/F	388.9	176.8	-55	-28	-9
169	**S-acetyl coenzyme A**	17.6	808.1	407.9	-220	-52	-27
170	**methylmalonyl coenzyme A**	17.8	866.1	408.0	-185	-58	-21
171	geranyl diphosphate	N/F	313.1	78.9	-65	-46	-1
172	β-hydroxy β-methylglutaryl-CoA	N/F	910.1	407.9	-220	-52	-27

173	**malonyl coenzyme A**	18.6	852.1	408.0	-185	-58	-21
174	ent-copal-8-ol diphosphate	N/F	467.2	78.8	-220	-52	-27
175	succinyl coenzyme A	N/F	866.1	407.6	-260	-56	-25
176	inositol-1,3,4,5-tetraphosphate	N/F	498.3	400.7	-100	-30	-27
177	**β-methylcrotonyl coenzyme A**	19.1	848.1	407.8	-185	-58	-21
178	1-diphosinositol pentakisphosphate	N/F	578.9	480.6	-25	-32	-31
179	**geranylgeranyl pyrophosphate**	19.2	449.2	78.8	-65	-68	-35
180	**isovaleryl coenzyme A**	19.2	850.2	407.9	-240	-58	-19
181	**acetoacetyl coenzyme A**	19.5	580.1	408.0	-220	-52	-27
182	phytic acid	N/F	658.9	560.7	-145	-38	-31
183	farnesyl diphosphate	N/F	381.1	78.9	-50	-50	-5

The analysis relied Waters ACQUITY UPLC H-Class UPLC System (Waters GmbH, Eschborn, Germany) coupled online to a hybrid triple quadrupole-linear ion trap mass spectrometer (QqLIT) AB Sciex QTRAP 6500 (AB Sciex, Darmstadt, Germany). Analytes detected in cyanobacterial extracts analysis are highlighted in bold.

[a]Molecular ion *m/z*

[b]Fragment ion *m/z*

Table S2-3 Patterns of metabolites (identified and non-identified) detected by GC-MS and LC-MS methods in indicated desiccation-tolerant cyanobacteria strains and showing significant (t-test, $p≤0.05$, U-test, $p≤0.01$) differences in relative content compared to the average content of these metabolites in all extremophilic strains studied.

Metabolite [a]	Chem. class[b]	TMS[c]	Strains tolerant to desiccation:								Method
			Nostocales, B-1520				*Nostocales*, B-1519				
			FC[d]	Student's t-test p^e	Welch's t-test p^f	Mann-Whitney U-test p^g	FC	Student's t-test *p*	Welch's t-test *p*	Mann-Whitney U-test *p*	
Salicylic acid	Ph	2TMS	14	<0.001	0.005	0.001					GC-MS
Erythritol	P	4TMS	4.4	0.02	0.003	0.008	6.5	<0.001	<0.001	0.001	GC-MS
Ethanolamine	A	3TMS	4.2	<0.001	0.04	0.004					GC-MS
RI1501 Unknown			3.8	0.009	0.024	0.006	4.5	<0.001	<0.001	0.003	GC-MS
RI1687 Unknown							2.2	<0.001	0.03	0.004	GC-MS
RI1299 Unknown							2.6	0.005	0.003	0.002	GC-MS

The metabolites were detected by GC-MS and LC-MS in polar extracts of cyanobacteria cultivated under storage conditions and showed significant differences (FC≥2, $p≤0.05$) in relative content in specified cyanobacterial strains compared to the average content of these metabolites in all extremophilic strains studied. [a] The metabolites are arranged according to their chemical structure: [b] phenolic acid and other phenolic compounds (Ph), polyol (P), amines (A), nonannotated compounds (unknowns). The latter compounds are specified with characteristic retention index, RI. [c] TMS – trimethylsilyl group. The features are specified with characteristic type and number of the TMS derivatization groups. [d] Fold difference (FC) in the mean abundances of individual metabolites. [e] The Student's *p*-value was calculated with the false discovery rate (FDR) obtained according to Benjamini-Hochberg approach and did not exceed the *p*-value threshold of 0.05 after applying the FDR correction [2]. [f], [g] The Welch's t-test *p*-value and Mann-Whitney U-test *p*-value do not exceed the *p*-value thresholds of 0.05 and 0.01, respectively.

Table S2–4 Patterns of identified and non-identified polar metabolites showing significant (t-test, $p \leq 0.05$, U-test, $p \leq 0.01$) differences in relative content in extreme temperature tolerant cyanobacterial strains compared to the average content of these metabolites in all extremophilic strains studied.

Metabolite [a]	Chem. class[b]	Feature[c]	Strains tolerant to extreme high or low temperatures: *Dolichospermum* sp., B-1213 (thermotolerant)				*Anabaena cf. pirinica*, B-1533 (cold-tolerant)				*Anabaena* sp., B-1535 (cold-tolerant)				Method
			FC[d]	t-test *p*[e]	Welch's t-test *p*[f]	Mann-Whitney U-test *p*[g]	FC	t-test *p*	Welch's t-test *p*	Mann-Whitney U-test *p*	FC	t-test *p*	Welch's t-test *p*	Mann-Whitney U-test *p*	
Glycolic acid	CA										2.4	<0.001	<0.001	0.003	LC-MS
Aconitic acid	CA						9	<0.001	0.005	0.001					LC-MS
3-Dehydroshikimic acid	CA						2.3	0.001	0.002	0.01	2.3	0.03	<0.001	0.009	LC-MS
RI3462 Trisaccharide	S		25	<0.001	0.002	0.001									GC-MS
Glucose	S	1MEOX, 5TMS (1)					12	<0.001							GC-MS
		1MEOX, 5TMS (2)					8.4	<0.001							GC-MS
RI2124 Sugar	S						2.3	0.001	0.002	0.006					GC-MS
Mannitol	P	6TMS									10	<0.001	0.002	0.002	GC-MS
2C-methyl-*D*-erythritol	P						17	<0.001	0.003	0.001					LC-MS
Digalacturonic acid	SA						5.1	<0.001	0.002	0.001	3.8	0.002	<0.001	0.007	LC-MS
Glucose 1-phosphate	SP		4	<0.001	0.011	0.001									LC-MS
Glucose 6-phosphate	SP						5.9	<0.001	<0.001	0.003					LC-MS
		1MEOX, 6TMS (1)					5.9	<0.001	<0.001	0.002					GC-MS
		1MEOX, 6TMS (2)					6.1	<0.001	<0.001	0.002					GC-MS
RI2247 C5-C6-Sugar-phosphate	SP						3.5	0.001	<0.001	0.004					GC-MS
Fructose 6-phosphate	SP						6.1	<0.001	<0.001	0.003					LC-MS
		1MEOX, 6TMS					5.1	<0.001	<0.001	0.002					GC-MS
2-Keto-3-deoxy-6-phosphogluconate	SP						6	<0.001	<0.001	0.004					LC-MS
2-C-methyl-*D*-erythritol-4-phosphate (MEP)	SP						4.2	0.03	0.002	0.014	5.4	<0.001	<0.001	0.004	LC-MS
Ribulose-5-phosphate/xylulose-5-phosphate	SP						3.6	<0.001	0.008	0.004					LC-MS
Phosphate	P_n		4	<0.001	0.002	0.001									LC-MS

Glutamic acid	AA		5.3	<0.001	0.011	0.001									LC-MS
Ureidosuccinic acid	AAd						9.3	<0.001	<0.001	0.005	9.7	<0.001	<0.001	0.002	LC-MS
NADH	Nuc		4.2	0.008	<0.001	0.008									LC-MS
cGMP	Nuc										5.8	<0.001	<0.001	0.001	LC-MS
ADP-ribose	NucS		27	<0.001	0.016	0.001									LC-MS
2-Hydroxypyridine	ON	1TMS	5.6	<0.001	<0.001	0.001									GC-MS
Dihydroorotic acid	ON										4.4	<0.001	0.001	0.001	LC-MS
Nonadecan-1-ol	FAl	1TMS					9.4	<0.001	<0.001	0.001					GC-MS
RI1016 Unknown							3.1	0.002	<0.001	0.002					GC-MS
RI1022 Unknown			56	< 0.001	0.011	0.001									GC-MS
RI1357 Unknown			6.2	< 0.001	0.030	0.001									GC-MS

The metabolites were detected by GC-MS and LC-MS in polar extracts of cyanobacteria cultivated under storage conditions and showed significant differences ($FC \geq 2$, $p \leq 0.05$) in relative content in specified cyanobacterial strains compared to the average content of these metabolites in all extremophilic strains studied. [a] Metabolites are presented and arranged according to their chemical structure: [b] carboxylic acids (CA), sugars (S), polyols (P), sugar derived acids (SA), sugar phosphates and derivatives (SP), phosphate (P_n), amino acids (AA) and derivatives (AAd), nucleotides and derivatives (Nuc), sugar nucleotides (NucS), other N-containing compounds (ON), fatty alcohol (FAl) and nonannotated compounds (unknowns). The latter compounds and compound annotated to a chemical class are specified with characteristic retention index, RI. [c] Specific metabolite features representing each metabolite. The features are specified with characteristic type and number of derivatization groups: TMS – trimethylsilyl group, MEOX – methyloxime group. [d] Fold difference (FC) in the mean abundances of individual metabolites. [e] The Student's t-test *p*-value was calculated with the FDR obtained according to Benjamini-Hochberg approach and did not exceed the *p*-value threshold of 0.05 after applying the FDR correction [2]. [f], [g] The Welch's t-test *p*-value and Mann-Whitney U-test *p*-value do not exceed the *p*-value thresholds of 0.05 and 0.01, respectively.

Table S2–5 Patterns* of identified and non-identified polar metabolites showing significant (t-test, $p \leq 0.05$, U-test, $p \leq 0.01$) differences in relative content in haloalkaliphilic, natronophilic cyanobacterial strains B-2037, B-353, B-2050 and B-1526 compared to the average content of these metabolites in all extremophilic strains studied.

			Sodalinema orleanskyi, B-2037				*Sodalinema gerasimenkoae, B-353*				*Sodalinema stali, B-2050*				*Limnospira* sp., *B-1526*				
Metabolite[a]	Chem. class[b]	Feature[c]	FC[d]	t-test *p*[e]	Welch's t-test *p*[f]	U-test *p*[g]	FC	t-test *p*	Welch's t-test *p*	U-test *p*	FC	t-test *p*	Welch's t-test *p*	U-test *p*	FC	t-test *p*	Welch's t-test *p*	U-test *p*	Method
3-Hydroxypyruvate	CA		0.34	0.04	<0.001	0.004													LC-MS
Lactic acid	CA	2TMS													3	0.01	0.002	0.006	GC-MS
3-Hydroxybutyric acid	CA	2TMS													20	<0.001	0.003	0.001	GC-MS
Isocitric acid	CA		4.9	<0.001	0.007	0.004					4.9	<0.001	0.006	0.004					LC-MS
Shikimic acid	CA						4.4	<0.001	<0.001	0.001									LC-MS
2-Phosphoglycolate	CAP		6.1	<0.001	0.006	0.002													LC-MS
Ribose	S	1MEOX, 4TMS									4.1	<0.001	<0.001	0.001					GC-MS
RI1756 C5-sugar	S						3.4	<0.001	0.011	0.002									GC-MS
RI3124 Disaccharide-derivate	S														4.9	<0.001	<0.001	0.001	GC-MS
RI2255 Glyceryl-glycoside 1	S	6TMS	10	<0.001	0.004	0.001													GC-MS
RI2310 Glyceryl-glycoside 2	S	6TMS													8.5	<0.001	0.002	0.001	GC-MS
Ribonic acid	SA										6.5	<0.001	0.002	0.001					LC-MS
D-Erythronic acid	SA	4TMS									2.8	0.001	<0.001	0.004					GC-MS
Galacturonic acid	SA										13	<0.001	0.01	0.001					LC-MS
Glycerol	P	3TMS									3.5	<0.001	<0.001	0.001					GC-MS
Sedoheptulose-1,7-biphosphate	SP		5.6	<0.001	0.048	0.004													LC-MS
Pyroglutamic acid	AA	2TMS													2.7	0.01	0.015	0.007	GC-MS
NADPH	Nuc										8.6	<0.001	0.012	0.001					LC-MS
ADP	Nuc		5.7	<0.001	<0.001	0.002													LC-MS
CDP	Nuc		5.8	<0.001	0.002	0.001													LC-MS
GDP	Nuc		4.9	<0.001	0.005	0.002													LC-MS
Adenosine	Nuc						41	<0.001	<0.001	0.001									LC-MS
Orotic acid	ON										5.0	<0.001	0.001	0.001					LC-MS
trans-9-Hexadecenoic acid	FA	1TMS	9.1	<0.001	0.006	0.001													GC-MS
Phosphoric acid	IO	3TMS	2.2	0.03	0.008	0.005													GC-MS

Chloride	IO	2.6	<0.001	0.009	0.003	2.5	<0.001	<0.001	0.004									LC-MS
RI1219 Unknown						9.7	<0.001	0.004	0.001									GC-MS
RI1320 Unknown						2.7	0.005	0.015	0.004									GC-MS
RI1349 Unknown						6.7	<0.001	0.004	0.001									GC-MS
RI1352 Unknown						7.8	<0.001	0.007	0.001									GC-MS
RI2359 Unknown														5.0	<0.001	0.004	0.002	GC-MS
RI3201 Unknown										0.33	0.007	0.011	0.003					GC-MS

The metabolites were detected by GC-MS and LC-MS in polar extracts of cyanobacteria cultivated under storage conditions and showed significant differences (FC≥2, t-test *p*≤0.05, U-test *p*≤0.01) in relative content in specified cyanobacterial strains compared to the average content of these metabolites in all extremophilic strains studied. [a] Metabolites presented and arranged according to their chemical structure: [b]carboxylic acids (CA) and their phosphate derivatives (CAP), sugars (S), sugar derived acids (SA), polyols (P), sugar phosphates (SP), amino acids (AA), nucleotides and nucleosides (Nuc), other N-containing compounds (ON), fatty acids (FA), inorganic ions (IO) and nonannotated compounds (unknowns). The latter compounds and compound annotated to a chemical class are specified with characteristic retention index, RI. [c] Metabolite features representing each metabolite are specified with characteristic type and number of derivatization groups: TMS – trimethylsilyl group, MEOX – methyloxime group. [d] Fold difference (FC) in the mean abundances of individual metabolites. [e] The Student's t-test *p*-value was calculated with the FDR obtained according to Benjamini-Hochberg approach and did not exceed the *p*-value threshold of 0.05 after applying the FDR correction [2]. [f], [g] The Welch's t-test *p*-value and Mann-Whitney U-test *p*-value do not exceed the *p*-value thresholds of 0.05 and 0.01, respectively.

Table S2-6 Patterns* of identified and non-identified polar metabolites showing significant (t-test, $p \leq 0.05$, U-test, $p \leq 0.01$) differences in relative content in haloalkaliphilic, natronophilic cyanobacterial strains B-1529, B-256 and B-287 compared to the average content of these metabolites in all extremophilic strains studied.

			Nodularia spumigena, B-1529				*Limnospira* sp., *B-287*				*Limnospira* sp., *B-256*				
Metabolite[a]	Chem. class[b]	Feature[c]	FC[d]	t-test *p*[e]	Welch's t-test *p*[f]	Mann-Whitney U-test *p*[g]	FC	t-test *p*	Welch's t-test *p*	Mann-Whitney U-test *p*	FC	t-test *p*	Welch's t-test *p*	Mann-Whitney U-test *p*	Method
Pyruvic acid	CA		4.1	<0.001	0.002	0.001									LC-MS
Glyceric acid	CA	3TMS					3.8	<0.001	0.004	0.001					GC-MS
Lactic acid	CA						2.4	0.04	<0.001	0.008	3.4	<0.001	0.007	0.001	LC-MS
		2TMS									8.8	<0.001	<0.001	0.002	GC-MS
alpha-Hydroxyglutaric acid	CA	3TMS					2.3	0.004	0.032	0.006					GC-MS
Citric acid	CA						6.3	<0.001	0.001	0.001					LC-MS
		4TMS					5.9	<0.001	0.011	0.002	3.7	0.02	<0.001	0.009	GC-MS
Succinic acid	CA						4.9	<0.001	0.001	0.001					LC-MS
		2TMS					4.3	<0.001	<0.001	0.001					GC-MS
Fumaric acid	CA						2.7	0.01	0.007	0.004					LC-MS
		2TMS	2.7	<0.001	<0.001	0.001									GC-MS
Malate	CA						3.3	0.007	0.004	0.004					LC-MS
		3TMS					3.8	<0.001	0.003	0.001					GC-MS
3-Dehydroshikimic acid	CA		2.3	0.003	0.031	0.007									LC-MS
3-Phosphoglyceric acid	CAP						14	<0.001	<0.001	0.001					LC-MS
2-Phosphoglyceric acid	CAP						18	<0.001	0.003	0.001					LC-MS
2-Phosphoglycolate	CAP						4.4	0.002	<0.001	0.008					LC-MS
Phosphoenolpyruvic acid	CAP						27	<0.001	<0.001	0.001					LC-MS
Fructose	S	1MEOX, 5TMS (1)	32	<0.001	0.001	0.001									GC-MS
		1MEOX, 5TMS (2)	35	<0.001	0.002	0.001									GC-MS
Mannose	S	1MEOX, 5TMS	17	<0.001	0.001	0.001									GC-MS
Galactose	S	1MEOX, 5TMS	17	<0.001	0.001	0.001									GC-MS
Glucose	S	1MEOX, 5TMS (1)	14	<0.001	0.008	0.01									GC-MS
		1MEOX, 5TMS (2)	4.0	0.01	0.002	0.009									GC-MS
Sucrose	S		4.6	<0.001	0.001	0.008									LC-MS
		8TMS	3.4	0.04	<0.001	0.001	8.5	<0.001	<0.001	0.009					GC-MS
α,α- Trehalose	S	8TMS					26	<0.001	<0.001	0.001					GC-MS

myo-Inositol	S	6TMS									8.3	<0.001	0.001	0.001	GC-MS
Glycerol	S	3TMS									2.8	0.005	<0.001	0.007	GC-MS
RI1756 C5-sugar	S		2.4	0.008	0.006	0.006									GC-MS
RI2124 Sugar	S		2.4	<0.001	0.006	0.003									GC-MS
RI2304 Sugar	S						12	<0.001	<0.001	0.001					GC-MS
Glucosyl glyceride peak 2	S	6TMS	4.0	0.005	<0.001	0.007									GC-MS
Gluconic acid	SA	6TMS	7.7	<0.001	<0.001	0.001									GC-MS
Galactonic acid	SA		13	<0.001	0.014	0.001									LC-MS
D-Erythronic acid	SA	4TMS					2.7	<0.001	0.016	0.005					GC-MS
Ribose-1-phosphate	SP		4.5	0.002	0.001	0.001									LC-MS
MEP	SP		4.5	0.002	0.029	0.009									LC-MS
2-Keto-3-deoxy-6-phosphogluconate	SP		6.7	<0.001	0.005	0.003									LC-MS
1-Deoxyxylulose 5-phosphate	SP										13	<0.001	0.006	0.001	LC-MS
2-Deoxyribose 5-phosphate	SP										12	<0.001	0.007	0.001	LC-MS
Ribose 5-phosphate	SP		4.5	<0.001	0.007	0.002									LC-MS
Fructose 6-phosphate	SP		6.1	<0.001	0.009	0.003									LC-MS
		1MEOX, 6TMS	3.4	0.002	0.021	0.006									GC-MS
Fructose-1,6-diphosphate	SP										6.6	<0.001	<0.001	0.003	LC-MS
Glucose 1-phosphate	SP						2.7	0.01	<0.001	0.008					LC-MS
Glucose 6-phosphate	SP		6	<0.001	0.008	0.003									LC-MS
		1MEOX, 6TMS (1)	4.5	<0.001	0.018	0.004									GC-MS
		1MEOX, 6TMS (2)	4.4	<0.001	0.016	0.006									GC-MS
Sedoheptulose 7-phosphate	SP		11	<0.001	0.007	0.001									LC-MS
Sedoheptulose-1,7-biphosphate	SP										4.8	<0.001	0.001	0.004	LC-MS
Glucosamine 6-phosphate	SP										14	<0.001	0.003	0.001	LC-MS
RI2102 C5-C6-Sugar-phosphate	SP						3.0	<0.001	0.001	0.004					GC-MS
RI2247 C5-C6-Sugar-phosphate	SP		3.5	<0.001	0.022	0.01									GC-MS
RI2014 Phosphate-conjugated compound	SP		3.3	<0.001	<0.001	0.004	3.0	0.001	0.018	0.007					GC-MS
Glycerol 3-phosphate	SP	4TMS					3.1	<0.001	<0.001	0.002					GC-MS
							3.4	0.003	<0.001	0.002	3.3	0.009	0.004	0.007	LC-MS

Alanine	AA	2TMS	10	<0.001	<0.001	0.001									GC-MS
Arginine	AA		17	<0.001	<0.001	0.001									LC-MS
Arginosuccinate	AA		11	<0.001	<0.001	0.001									LC-MS
Aspartic acid	AA		11	<0.001	<0.001	0.001									LC-MS
		2TMS	7.2	<0.001	<0.001	0.001									GC-MS
		3TMS	25	<0.001	0.006	0.001									GC-MS
Serine	AA		25	<0.001	0.002	0.001									LC-MS
Threonine	AA		46	<0.001	0.001	0.001									LC-MS
Valine	AA	2TMS	16	<0.001	<0.001	0.001									GC-MS
Isoleucine	AA	2TMS	25	<0.001	<0.001	0.001									GC-MS
Glycine	AA		15	<0.001	0.001	0.001									LC-MS
		2TMS	24	<0.001	0.004	0.001									GC-MS
Phenylalanine	AA		43	<0.001	<0.001	0.001									LC-MS
Tryptophan	AA		33	<0.001	0.004	0.001									LC-MS
Tyrosine	AA	2TMS	30	<0.001	0.003	0.001									GC-MS
		3TMS	12	<0.001	0.003	0.001									GC-MS
Methionine	AA		80	<0.001	0.002	0.001									LC-MS
Glutamine	AA		34	<0.001	0.002	0.001									LC-MS
Citrulline	AA		7.7	<0.001	0.004	0.001									LC-MS
Glutamic acid	AA	2TMS					6.0	<0.001	0.001	0.001					GC-MS
Pyroglutamic acid	AA	2TMS					2.6	0.004	0.007	0.002					GC-MS
S-adenosyl-*L*-homocysteine	AA										6.2	<0.001	0.002	0.001	LC-MS
Cytidine	Nuc		14	<0.001	<0.001	0.001									LC-MS
Uridine	Nuc		21	<0.001	<0.001	0.001									LC-MS
Guanosine	Nuc		22	<0.001	<0.001	0.001									LC-MS
2'-Deoxyguanosine	Nuc		19	<0.001	<0.001	0.001									LC-MS
Adenylosuccinic acid	Nuc						4.0	0.008	0.017	0.007	7.6	<0.001	0.001	0.001	LC-MS
Xanthosine-5'-phosphate	Nuc						4.1	<0.001	0.005	0.004	4.6	<0.001	<0.001	0.002	LC-MS
Inosinic acid	Nuc						3.8	0.001	<0.001	0.008	5.7	<0.001	0.002	0.001	LC-MS
cAMP	Nuc						4.3	<0.001	<0.001	0.001					LC-MS
AMP	Nuc						3.9	<0.001	<0.001	0.006	5.5	<0.001	0.002	0.002	LC-MS
CMP	Nuc		6.8	<0.001	0.010	0.001									LC-MS
UMP	Nuc						2.7	<0.001	<0.001	0.003					LC-MS

UDP	Nuc						3.1	<0.001	<0.001	0.001					LC-MS
GDP	Nuc						4.3	<0.001	<0.001	0.004					LC-MS
ADP	Nuc						5.3	<0.001	<0.001	0.004					LC-MS
CTP	Nuc						4.1	<0.001	0.008	0.003					LC-MS
ATP	Nuc						10	<0.001	0.001	0.001					LC-MS
GTP	Nuc						10	<0.001	0.007	0.001					LC-MS
UTP	Nuc						8.2	<0.001	0.006	0.001					LC-MS
dAMP	Nuc		9.4	<0.001	0.012	0.001									LC-MS
dATP	Nuc						3.8	<0.001	0.018	0.004					LC-MS
dGMP	Nuc						3.8	<0.001	<0.001	0.006	4.3	<0.001	<0.001	0.002	LC-MS
dTMP	Nuc		3.0	<0.001	0.026	0.007					2.6	0.02	<0.001	0.009	LC-MS
TDP	Nuc										3.0	<0.001	<0.001	0.002	LC-MS
dTTP	Nuc						7.5	<0.001	0.004	0.002	5.6	<0.001	0.006	0.004	LC-MS
FAD	Nuc		3.8	<0.001	0.014	0.006	4.4	<0.001	0.010	0.003					LC-MS
NAD^+	Nuc						4.8	<0.001	0.001	0.005	5.6	<0.001	<0.001	0.002	LC-MS
NADH	Nuc						6.9	<0.001	0.001	0.001					LC-MS
UDP-glucose	NucS						3.1	0.009	<0.001	0.008	4.8	<0.001	<0.001	0.001	LC-MS
ADP-glucose	NucS						7.4	<0.001	<0.001	0.004	8.9	<0.001	0.003	0.002	LC-MS
UDP-*N*-acetylglucosamine	NucS										3.8	<0.001	<0.001	0.001	LC-MS
Glyceraldehyde 3-phosphate	SP						3.3	0.002	0.002	0.003					LC-MS
3-Ureidopropionic acid	ON		74	<0.001	<0.001	0.001									LC-MS
Pantothenic acid	ON		7.4	<0.001	0.001	0.001									LC-MS
Nicotinic acid	ON		5.7	<0.001	0.007	0.001									LC-MS
Uric acid	ON		42	<0.001	0.002	0.001									LC-MS
Malonyl-CoA	CoAt						8.3	<0.001	0.005	0.001					LC-MS
beta-Methylcrotonyl-CoA	CoAt		9.4	<0.001	0.002	0.001	4.1	0.02	0.047	0.009					LC-MS
Methylmalonyl-CoA	CoAt		7.8	<0.001	0.002	0.001									LC-MS
Isovaleryl-CoA	CoAt		13	<0.001	0.006	0.001									LC-MS
Acetoacetyl-CoA	CoAt		13	<0.001	0.006	0.001									LC-MS
S-acetyl-CoA	CoAt						11	<0.001	0.001	0.001	7.0	<0.001	<0.001	0.007	LC-MS
Palmitic acid	FA	1TMS	9.7	<0.001	<0.001	0.001									GC-MS
trans-9-Octadecenoic acid	FA	1TMS	18	<0.001	<0.001	0.001									GC-MS
Dodecanoic acid	FA	1TMS	2.3	<0.001	0.003	0.002									GC-MS

Linoleic acid	FA	1TMS	18	<0.001	<0.001	0.001									GC-MS
Tetradecanoic acid	FA	1TMS	3.0	<0.001	0.001	0.001									GC-MS
Oleic acid	FA	1TMS	30	<0.001											GC-MS
cis-9-Hexadecenoic acid	FA	1TMS	14	<0.001	<0.001	0.001									GC-MS
1-Monopalmitoylglycerol	FA	2TMS					4.2	<0.001	0.008	0.002					GC-MS
Phytol	PP	1TMS	3.3	0.002	0.001	0.004									GC-MS
(2E)-4-hydroxy-3-methylbut-2-en-1-yl diphosphate	PP						8.2	<0.001	<0.001	0.002					LC-MS
delta3-isopentenyl pyrophosphate	PP						10	<0.001	<0.001	0.001					LC-MS
Dimethylallylpyrophosphate	PP						10	<0.001	0.006	0.001					LC-MS
Geranylgeranyl pyrophosphate	PP						5.5	<0.001	0.023	0.001					LC-MS
RI1087 Unknown			2.6	0.003	<0.001	0.01	3.9	<0.001	<0.001	0.001					GC-MS
RI1188 Unknown											7.0	<0.001	<0.001	0.002	GC-MS
RI1320 Unknown			2.7	<0.001	<0.001	0.006									GC-MS
RI1329 Unknown			2.3	0.004	0.002	0.005									GC-MS
RI1612 Unknown			18	<0.001	0.004	0.001									GC-MS
RI1956 Unknown			2.4	<0.001	<0.001	0.001									GC-MS
RI2408 Unknown			38	<0.001	<0.001	0.001									GC-MS

* Metabolites were detected by GC-MS and LC-MS in polar extracts of cyanobacteria cultivated under storage conditions and showed significant differences (FC≥2, $p \leq 0.05$, U-test $p \leq 0.01$) in relative content in specified cyanobacterial strains compared to the average content of these metabolites in all extremophilic strains studied. [a] Metabolites arranged according to their chemical structure: [b]carboxylic acids (CA) and their phosphate derivatives (CAP), sugars (S), sugar derived acids (SA), sugar phosphates (SP), amino acids (AA), nucleotides and nucleosides (Nuc), other N-containing compounds (ON), CoA thioesters (CoAt), fatty acids (FA), prenyl phosphates (PP) and nonannotated compounds (unknowns). The latter compounds and compound annotated to a chemical class are specified with characteristic retention index, RI. [c] Specific metabolite features representing each metabolite. The features are specified with characteristic type and number of derivatization groups: TMS – trimethylsilyl group, MEOX – methyloxime group. [d] Fold difference (FC) in the mean abundances of individual metabolites. [e] The Student's t-test *p*-value was calculated with the FDR obtained according to Benjamini-Hochberg approach and did not exceed the *p*-value threshold of 0.05 after applying the FDR correction [2]. [f] , [g] The Welch's t-test *p*-value and Mann-Whitney U-test *p*-value do not exceed the *p*-value thresholds of 0.05 and 0.01, respectively.

Table S2-7 Patterns* of identified and non-identified polar metabolites showing significant (t-test, $p \leq 0.05$) differences in relative content in diazotrophic heterocystous (B-1520, B-1519, B-1213, B-1533, B-1535, B-1529) compared to the non-heterocystous extremophilic cyanobacterial strains studied (B-2037, B-2050, B-353, B1526, B-256, B-287).

Metabolite [a]	Chem. class[b]	Feature[c]	FC[d]	T-test *p*[e]	Method
Metabolites with higher abundances in heterocyst-forming compared to non-heterocystous strains					
Isocitric acid	CA	4TMS	4.8	0.013	GC-MS
Salicylic acid	CA	2TMS	17.8	0.009	GC-MS
Glucose	S		5.5	0.030	LC-MS
Mannose	S	1MEOX, 5TMS	6.8	0.031	GC-MS
RI3462 Trisaccharide	S		18.5	0.029	GC-MS
Mannitol	P	6TMS	5.0	0.031	GC-MS
2C-methyl-*D*-erythritol	P		43	0.015	LC-MS
Digalacturonic acid	SA		3.3	0.005	LC-MS
Fructose 6-phosphate	SP		5.3	0.010	LC-MS
Glucose 6-phosphate	SP		5.0	0.011	LC-MS
Sedoheptulose 7-phosphate	SP		28	0.002	LC-MS
RI2542 C7-6 Sugar-phosphate	SP		7.5	0.027	GC-MS
2C-methylerythritol 4-phosphate	SP		31	0.0003	LC-MS
2-keto-3-deoxy-6-phosphogluconate	SAP		5.1	0.015	LC-MS
Ethanolamine	A	3TMS	4.2	0.001	GC-MS
Tyrosine	AA	2TMS	7.2	0.027	GC-MS
Argininosuccinate	AA		28	0.002	LC-MS
Ureidosuccinic acid	AA		37	0.008	LC-MS
Nonadecan-1-ol	Fal	1TMS	5.7	0.007	GC-MS
RI1516 Phenolic compound	Ph		4.1	0.039	GC-MS
Phosphate	IO		2.5	0.005	LC-MS
RI1501 Unknown			3.5	0.005	GC-MS
RI3622 Unknown			2.2	0.016	GC-MS
Metabolites with lower abundances in heterocyst-forming compared to non-heterocystous strains					
Lactic acid		2TMS	4.6	0.00001	GC-MS
	CA		3.7	0.043	LC-MS
Malate		3TMS	2.1	0.040	GC-MS
	CA		2.7	0.027	LC-MS
Fumaric acid	CA		2.3	0.022	LC-MS
Succinic acid	CA	2TMS	2.9	0.005	GC-MS
Citric acid	CA	4TMS	4.8	0.008	GC-MS
Glyceric acid	CA	3TMS	2.6	0.001	GC-MS
shikimic acid	CA		3.8	0.0003	LC-MS
2-phosphoglycolic acid	CAP		14	0.000	LC-MS
2-phosphoglyceric acid	CAP		39	0.009	LC-MS
3-phosphoglyceric acid	CAP		24	0.005	LC-MS
Glycerophosphoric acid	CAP		3.8	0.003	LC-MS
Phosphoenolpyruvic acid	CAP		25	0.024	LC-MS
Ribose	S	1MEOX, 4TMS	2.2	0.013	GC-MS
RI2304 Carbohydrate	S		422	0.001	GC-MS
RI2255 Glucosylglycerol 1	S		182	0.0005	GC-MS

RI2310 Glucosylglycerol 2	S		3.9	0.020	GC-MS
α, α-Trehalose	S	8TMS	65	0.017	GC-MS
Glycerol	P	3TMS	4.6	0.000	GC-MS
D-Erythronic acid	SA	4TMS	2.5	0.001	GC-MS
Fructose-1,6-diphosphate	SP		27	0.001	LC-MS
Sedoheptulose-1,7-biphosphate	SP		9.4	0.001	LC-MS
Glycerol 3-phosphate	SP	4TMS	2.9	0.001	GC-MS
Glyceraldehyde 3-phosphate	SP		2.5	0.023	LC-MS
Pyroglutamic acid	AA	2TMS	2.3	0.029	GC-MS
Adenylosuccinic acid	Nuc		6.5	0.004	LC-MS
GTP	Nuc		17	0.002	LC-MS
ATP	Nuc		22	0.001	LC-MS
CTP	Nuc		5.6	0.000	LC-MS
UTP	Nuc		7.2	0.004	LC-MS
CDP	Nuc		3.7	0.007	LC-MS
GDP	Nuc		5.0	0.001	LC-MS
ADP	Nuc		6.6	0.002	LC-MS
dTTP	Nuc		5.5	0.013	LC-MS
NADPH	Nuc		3.5	0.031	LC-MS
ADP-glucose	NucS		7.4	0.019	LC-MS
S-acetyl CoA	CoAt		10	0.016	LC-MS
trans-9-Hexadecenoic acid	FA	1TMS	5.7	0.008	GC-MS
(2E)-4-hydroxy-3-methylbut-2-en-1-yl diphosphate	PP		8.9	0.003	LC-MS
*delta*3-isopentenyl pyrophosphate	PP		10.4	0.001	LC-MS
Dimethylallylpyrophosphat	PP		4.2	0.028	LC-MS
Chloride	IO		2.2	0.001	LC-MS
RI1219 Unknown			3.7	0.030	GC-MS

* Metabolites were detected by GC-MS and LC-MS in polar extracts of cyanobacteria cultivated under storage conditions and showed significant differences (FC≥2, $p \leq 0.05$) in relative content in diazotrophic heterocystous (B-1520, B-1519, B-1213, B-1533, B-1535, B-1529; n=24) compared to non-heterocystous (B-353, B-2050, B-2037, B-1526, B-287, B-256; n=24) extremophilic strains studied. [a] Metabolites are arranged according to their chemical structure: [b]carboxylic acids (CA) and their phosphate derivatives (CAP), sugars (S), sugar derived acids (SA), sugar phosphates (SP), amino acids (AA), nucleotides and nucleosides (Nuc), sugar nucleotides (NucS), CoA thioesters (CoAt), fatty acids (FA) and their alcohols (FAl), phenolic compounds (Ph), prenyl phosphates (PP), inorganic iones (IO) and nonannotated compounds (unknowns). The latter compounds and compound annotated to a chemical class are specified with characteristic retention index, RI. [c] Specific metabolite features representing each metabolite and are specified with characteristic type and number of derivatization groups: TMS – trimethylsilyl group, MEOX – methyloxime group. [d] Fold difference (FC) in the mean abundances of individual metabolites. [e] The Student's t-test *p*-value was calculated with the FDR obtained according to Benjamini-Hochberg approach and did not exceed the *p*-value threshold of 0.05 after applying the FDR correction [2].

Table S2-8 The most important metabolic pathways* for the haloalkalophilic and natronophilic cyanobacterial strains with appeared considerable intergroup variability revealed by the Metaboanalyst pathway analysis.

#	Metabolic pathways[a]	Key metabolites[b]	Metabolic pathways selected by PI value =1[c]		
			**B-1529**	**B-287**	**B-256**
**1**	Butanoate metabolism	Succinate; Acetyl-CoA; Fumarate; Malate; Glutamate		+	
**2**	Inositol phosphate metabolism	*myo*-Inositol; Glyceraldehyde 3-phosphate; Acetyl-CoA		+	+
**3**	Pentose and glucuronate interconversions	UDP-glucose; Glycerol			+
**4**	Galactose metabolism	Glucose; Galactose; Fructose; UDP-glucose; Glucose 6-phosphate; Glyceraldehyde 3-phosphate; Fructose 6-phosphate; Sucrose	+		+
**5**	Alanine, aspartate and glutamate metabolism	Aspartate; *N*-(Arginino)succinate; Alanine; Pyruvate; Glutamine; Fumarate; *N*-Carbamoylaspartate	+		

*The impact of all matched paths is shown according to the *p* values ($p \leq 0.05$, FDR correction) obtained from the pathway enrichment analysis and the pathway impact values obtained from the path topology analysis.

[a] The most significant metabolic pathways identified with established threshold values: $p \leq 0.05$ corresponding to -log10(p) ≥1.3.

[b] Metabolites affiliated with specific metabolic pathway showing statistically significant differences in their relative content between the compared groups.

[c] The highest impact values (IP =1) obtained from the pathway topology analysis for pathways selected by pathway enrichment analysis with *p* (FDR correction) ≤0.05.

For the corresponding spotted pathway diagrams, see Figure S2-14.

Table S2-9 Gas chromatographic (GC) separation conditions and electron ionization-quadrupole-mass spectrometry (EI-Q-MS) settings for analysis of extremophilic cyanobacteria primary polar thermally stabile metabolites.

**Parameters**	**Setting**
	**GC settings**
Separation column	Phenomenex ZB-5MS (30 m × 0.25 mm ID, 0.25 µm film thickness)
Carrier gas /<br>carrier gas flow rate	Helium / 1 mL/min
Injector operation mode	Splitless mode<br>(90 s splitless time)
Injector temperature	250°C
Temperature program	1 min at 40°C<br>ramp 15°C/min to 70°C<br>1 min at 70°C<br>ramp 6°C/min to 320°C<br>12 min at 320°C
**Parameters**	**MS settings**
Ionization mode	Electron ionization (EI)
Electron energy	70 eV
Operation mode	scanning at 0.34 sec $scan^{-1}$
m/z range	50 - 700

The analysis was accomplished with Shimadzu GC2010 gas chromatograph coupled online to a quadrupole mass selective detector Shimadzu GCMS QP2010 with CTC GC PAL Liquid Injector (Shimadzu Scientific Instruments, Australia).

Table S2-10 Kovats retention indices (RI) of linear alkanes (C10–C40).

RI	t_R[a] (min)	S/N[b]	Alkane	*m/z*[c]
1100	9.536	301	$C_{11}H_{24}$	156
1200	11.898	214	$C_{12}H_{26}$	170
1300	14.403	62	$C_{13}H_{28}$	184
1400	16.906	54	$C_{14}H_{30}$	198
1500	18.953	59	$C_{15}H_{32}$	212
1600	20.842	71	$C_{16}H_{34}$	226
1700	22.641	88	$C_{17}H_{36}$	240
1800	24.373	103	$C_{18}H_{38}$	254
1900	26.035	133	$C_{19}H_{40}$	268
2000	27.633	120	$C_{20}H_{42}$	282
2100	29.163	566	$C_{21}H_{44}$	296
2200	30.654	624	$C_{22}H_{46}$	310
2300	32.086	659	$C_{23}H_{48}$	324
2400	33.462	708	$C_{24}H_{50}$	338
2500	34.787	766	$C_{25}H_{52}$	352
2600	36.063	762	$C_{26}H_{54}$	366
2700	37.295	818	$C_{27}H_{56}$	380
2800	38.482	868	$C_{28}H_{58}$	394
2900	39.629	832	$C_{29}H_{60}$	408
3000	40.742	804	$C_{30}H_{62}$	422
3100	41.82	756	$C_{31}H_{64}$	436
3200	42.859	791	$C_{32}H_{66}$	450
3300	43.873	750	$C_{33}H_{68}$	464
3400	44.854	693	$C_{34}H_{70}$	478
3500	45.812	685	$C_{35}H_{72}$	492
3600	46.832	519	$C_{36}H_{74}$	506
3700	47.986	501	$C_{37}H_{76}$	520
3800	49.31	499	$C_{38}H_{78}$	534
3900	50.877	487	$C_{39}H_{80}$	548
4000	52.723	480	$C_{40}H_{82}$	562

[a]Retention time of linear alkanes analytes (C10–C40).
[b]Signal to noise ratio.
[c]*m/z* value of molecular ion of linear alkane.

Figures

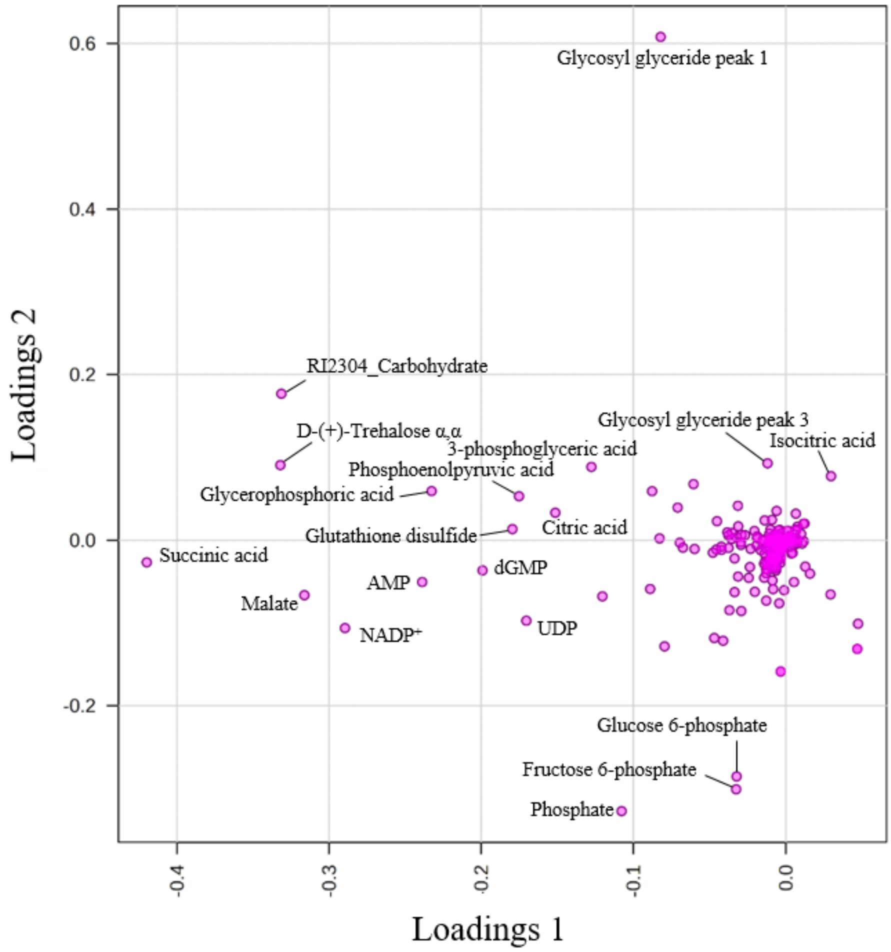

Figure S2-1 Principal component analysis (PCA) presenting loadings plot of the studied extremophilic cyanobacterial strains. The corresponding score plot is showed in Figure 1.

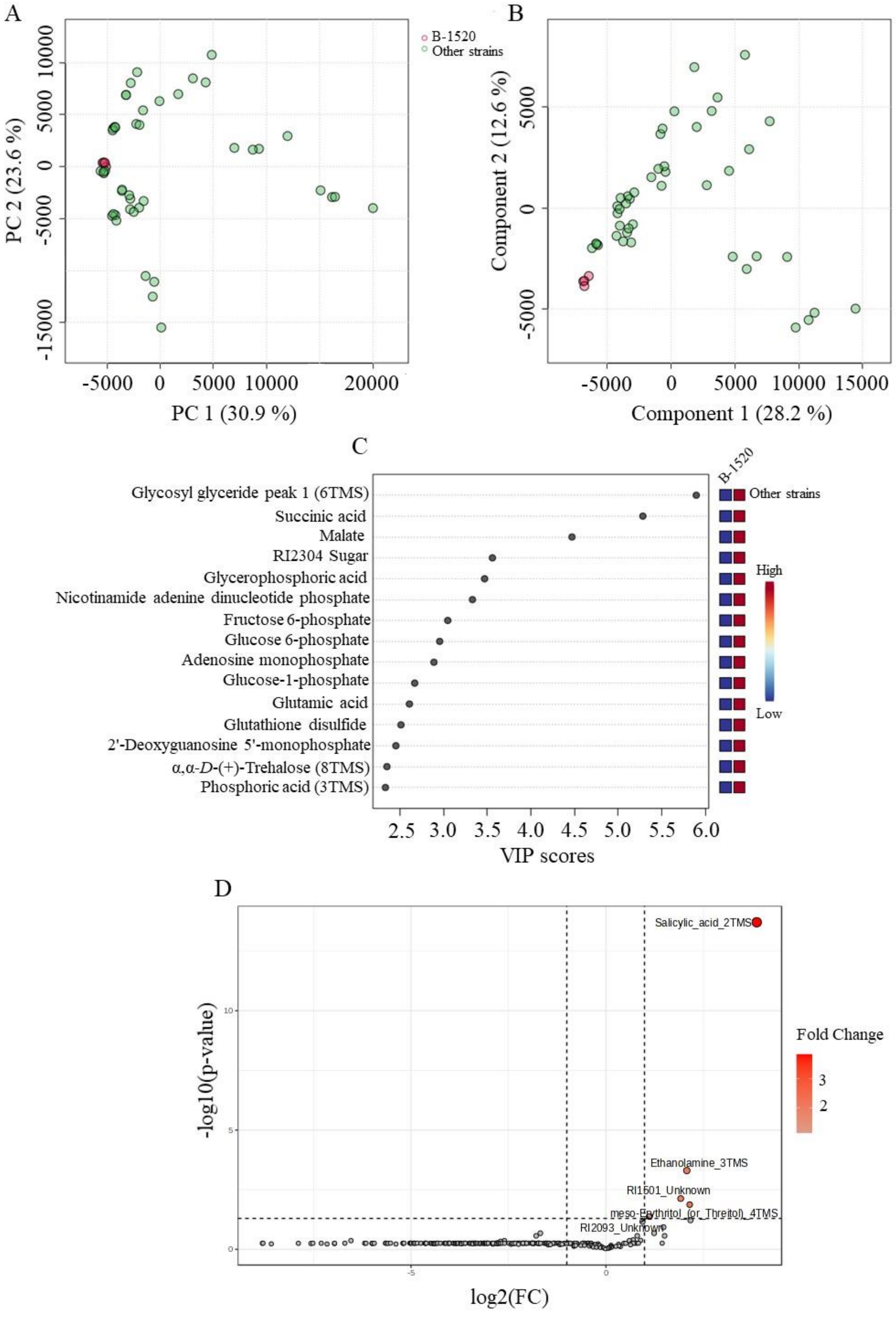
A
B-1520
Other strains
PC 2 (23.6 %)
PC 1 (30.9 %)
10000
5000
0
-5000
-15000
-5000
0
5000
10000
20000
B
Component 2 (12.6 %)
Component 1 (28.2 %)
5000
0
-5000
-5000
0
5000
10000
15000
C
B-1520
Other strains
Glycosyl glyceride peak 1 (6TMS)
Succinic acid
Malate
RI2304 Sugar
Glycerophosphoric acid
Nicotinamide adenine dinucleotide phosphate
Fructose 6-phosphate
Glucose 6-phosphate
Adenosine monophosphate
Glucose-1-phosphate
Glutamic acid
Glutathione disulfide
2'-Deoxyguanosine 5'-monophosphate
α,α-D-(+)-Trehalose (8TMS)
Phosphoric acid (3TMS)
High
Low
2.5
3.0
3.5
4.0
4.5
5.0
5.5
6.0
VIP scores
D
Salicylic_acid_2TMS
Ethanolamine_3TMS
RI1601_Unknown
meso-Erythritol_(or_Threitol)_4TMS
RI2093_Unknown
Fold Change
3
2
-log10(p-value)
log2(FC)

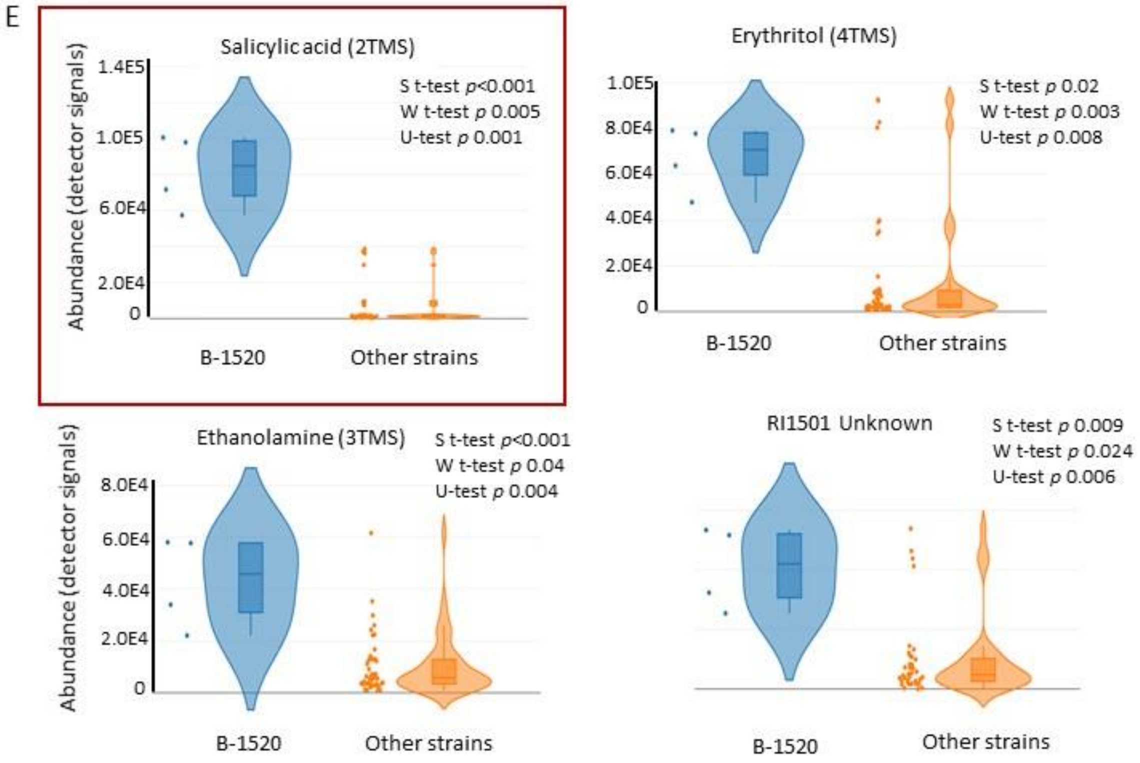

Figure S2-2 Comprehensive statistical analysis of polar metabolite profiles of the extremophilic cyanobacteria strain *Nostoc commune* B-1520 (desiccation-tolerant). This analysis was performed by comparing the metabolite profiles of this strain with the metabolomes of all other eleven cyanobacterial strains studied. PCA (A) and PLS-DA (B) score plots built for the first two principal components, (C) – VIP scores plot based on PLS-DA shows 15 metabolites contributing most to the difference between the compared groups, (D) – Volcano plot illustrates (colored dots) metabolites with statistically significant (Student's (S) t-test, *p*-value ≤0.05, FDR-corrected) difference in relative content between the compared groups (FC ≥2), (E) – Box plots built for the strain-specific metabolites validated by Welch's t-test (W test *p*-value ≤0.05) and Mann-Whitney U-test (U test *p*-value ≤0.01). The red frame indicates a metabolite displaying the most significant difference (S t-test *p*-value (FDR adjusted) <0.01, Welch t-test *p*-value <0.01, U-test *p*-value<0.001) in the content between B-1520 and other strains.

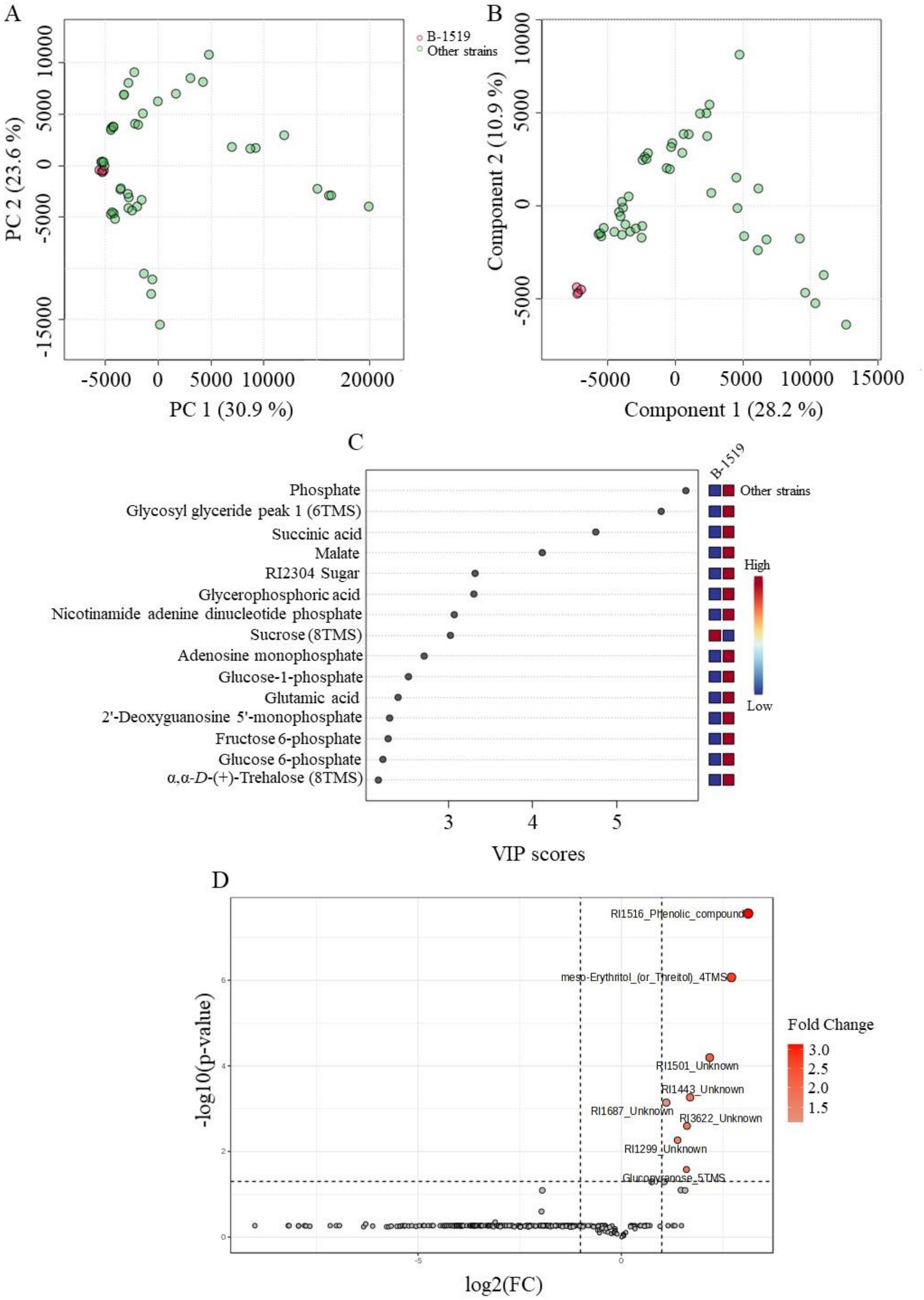
A
B-1519
Other strains
PC 2 (23.6 %)
PC 1 (30.9 %)
B
Component 2 (10.9 %)
Component 1 (28.2 %)
C
B-1519
Other strains
Phosphate
Glycosyl glyceride peak 1 (6TMS)
Succinic acid
Malate
RI2304 Sugar
Glycerophosphoric acid
Nicotinamide adenine dinucleotide phosphate
Sucrose (8TMS)
Adenosine monophosphate
Glucose-1-phosphate
Glutamic acid
2'-Deoxyguanosine 5'-monophosphate
Fructose 6-phosphate
Glucose 6-phosphate
α,α-D-(+)-Trehalose (8TMS)
High
Low
VIP scores
D
RI1516_Phenolic_compound
meso-Erythritol_(or_Threitol)_4TMS
RI1501_Unknown
RI1443_Unknown
RI1687_Unknown
RI3622_Unknown
RI1299_Unknown
Glucopyranose_5TMS
Fold Change
-log10(p-value)
log2(FC)

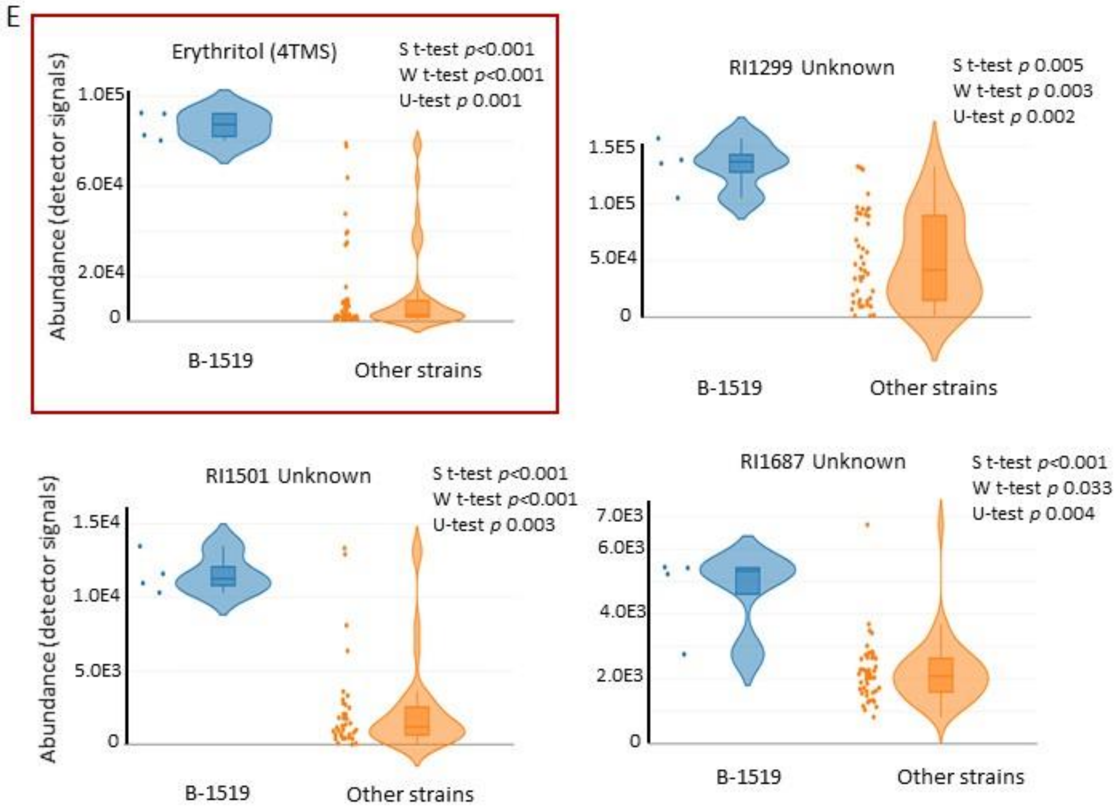

Figure S2-3 Comprehensive statistical analysis of polar metabolite profiles of the extremophilic cyanobacteria strain *Nostoc commune* B-1519 (desiccation-tolerant). This analysis was performed by comparing the metabolite profiles of this strain with the metabolomes of all other cyanobacterial strains studied. PCA (A) and PLS-DA (B) score plots built for the first two principal components, (C) – VIP scores plot based on PLS-DA shows 15 metabolites contributing most to the difference between the compared groups, (D) – Volcano plot illustrates (colored dots) metabolites with statistically significant (t-test, p-value ≤ 0.05, FDR-corrected) difference in relative content between the compared groups (FC ≥ 2), (E) – Box plots built for the strain-specific metabolites validated additionally by Welch's t-test (p-value ≤ 0.05) and Mann-Whitney U-test (p-value ≤ 0.01). The red frame indicates a metabolite displaying the most significant difference (T-test p-value (FDR adjusted) <0.01, Welch t-test p-value <0.01, U-test p-value<0.001) in the content between B-1519 and other strains.

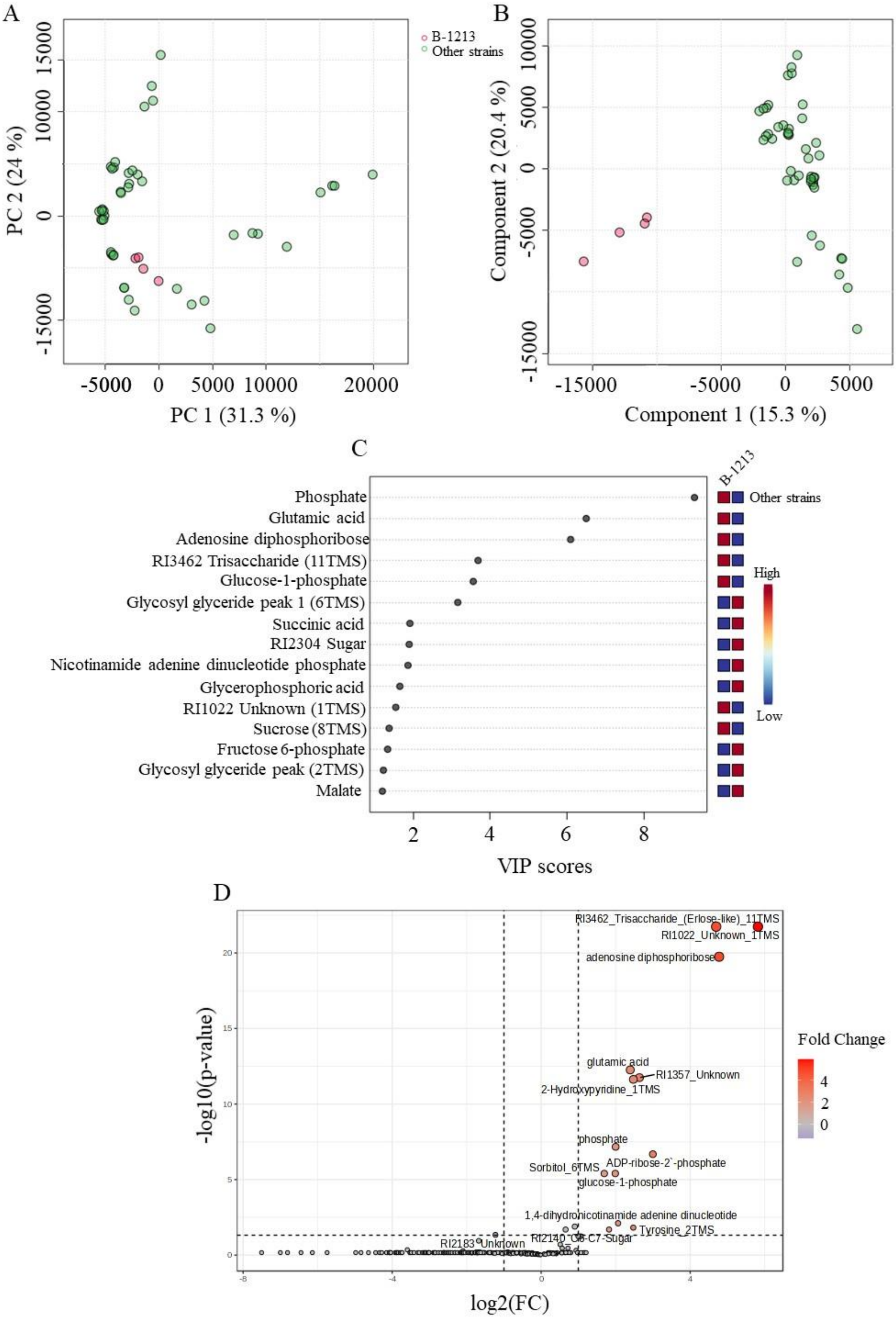

A
B-1213
Other strains
PC 2 (24 %)
PC 1 (31.3 %)
B
Component 2 (20.4 %)
Component 1 (15.3 %)
C
B-1213
Other strains
Phosphate
Glutamic acid
Adenosine diphosphoribose
RI3462 Trisaccharide (11TMS)
Glucose-1-phosphate
Glycosyl glyceride peak 1 (6TMS)
Succinic acid
RI2304 Sugar
Nicotinamide adenine dinucleotide phosphate
Glycerophosphoric acid
RI1022 Unknown (1TMS)
Sucrose (8TMS)
Fructose 6-phosphate
Glycosyl glyceride peak (2TMS)
Malate
High
Low
VIP scores
D
RI3462_Trisaccharide_(Erlose-like)_11TMS
RI1022_Unknown_1TMS
adenosine diphosphoribose
glutamic acid
RI1357_Unknown
2-Hydroxypyridine_1TMS
phosphate
ADP-ribose-2`-phosphate
Sorbitol_6TMS
glucose-1-phosphate
1,4-dihydronicotinamide adenine dinucleotide
Tyrosine_2TMS
RI2140_C6-C7-Sugar
RI2183_Unknown
-log10(p-value)
log2(FC)
Fold Change

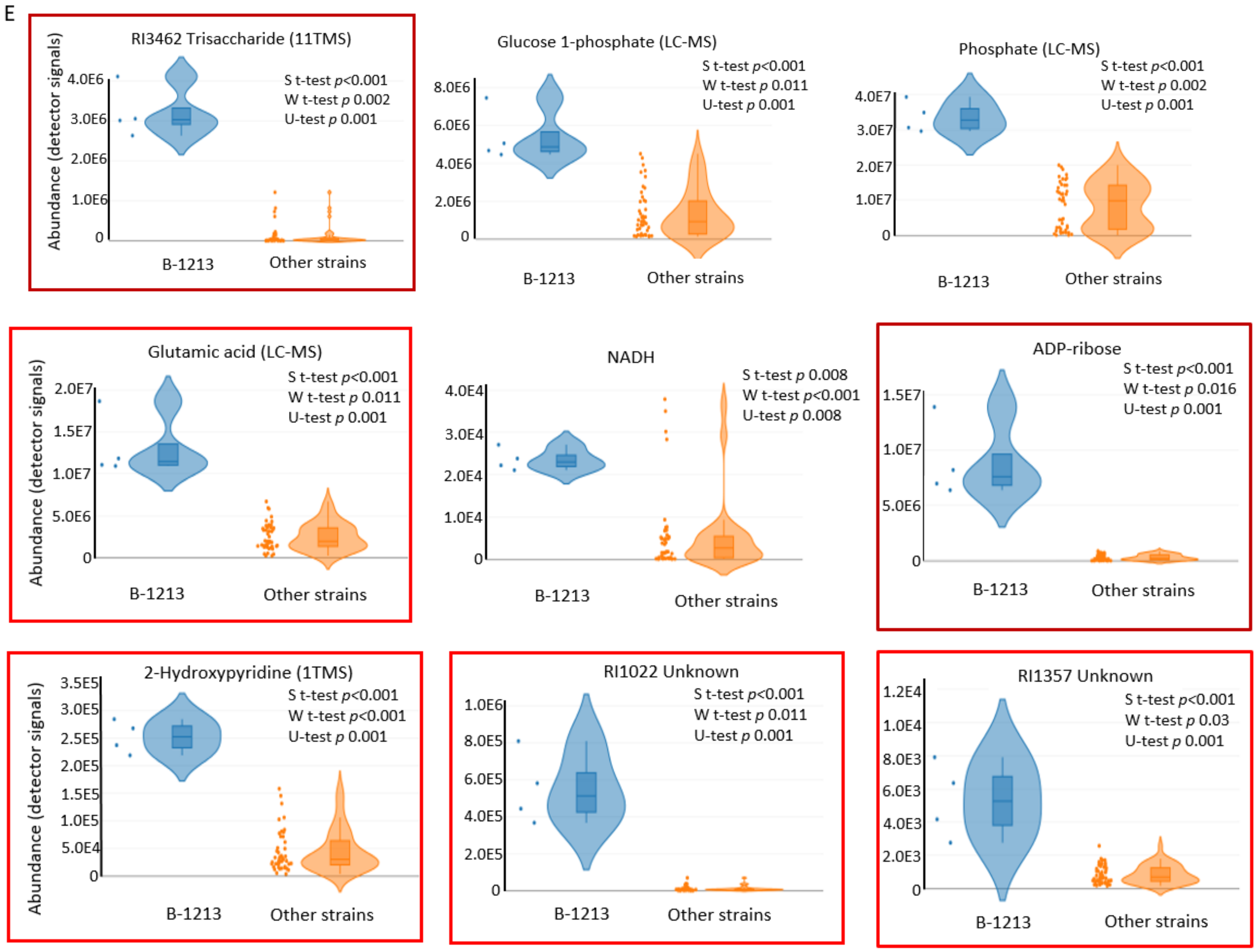

Figure S2-4 Comprehensive statistical analysis of polar metabolite profiles of the extremophilic cyanobacteria strain *Dolichospermum* sp. B-1213 (high temperature tolerant). This analysis was performed by comparing the metabolite profiles of this strain with the metabolomes of all other cyanobacterial strains studied. PCA (A) and PLS-DA (B) score plots built for the first two principal components, (C) – VIP scores plot based on PLS-DA shows 15 metabolites contributing most to the difference between the compared groups, (D) – Volcano plot illustrates (colored dots) metabolites with statistically significant (t-test, *p*-value ≤0.05, FDR-corrected) difference in relative content between the compared groups (FC ≥2), (E) – Box plots built for the strain-specific metabolites validated additionally by Welch's t-test (W test *p*-value ≤0.05) and Mann-Whitney U-test (U test *p*-value ≤0.01). The red frame indicates metabolites displaying the most significant difference (S t-test *p*-value (FDR adjusted) <0.01, Welch t-test *p*-value <0.01, U-test *p*-value<0.001) in the content between B-1213 and other strains.

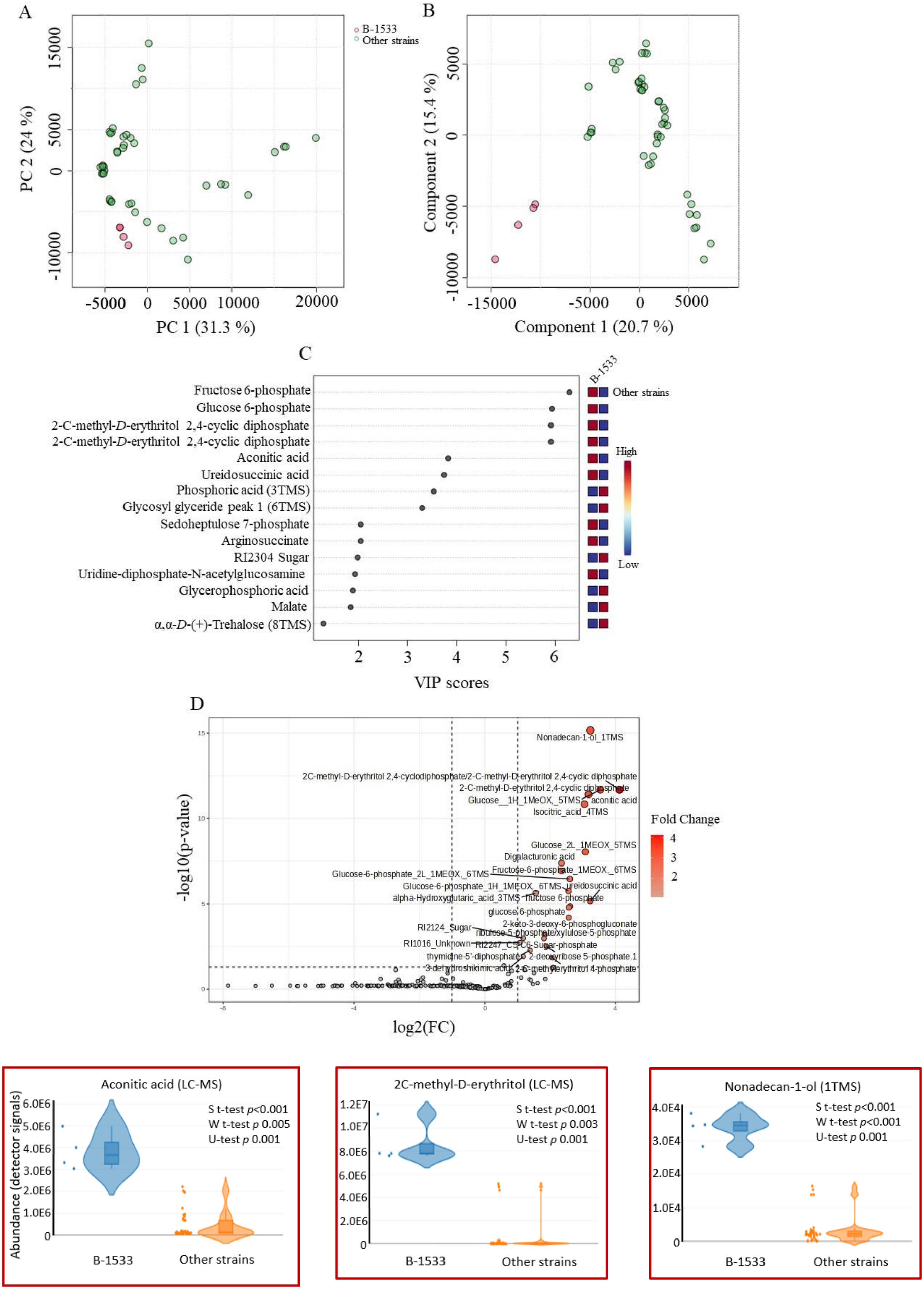

A
B-1533
Other strains
PC 2 (24 %)
PC 1 (31.3 %)
-5000 0 5000 10000 20000
-10000 0 5000 15000
B
Component 2 (15.4 %)
Component 1 (20.7 %)
-15000 -5000 0 5000
-10000 -5000 0 5000
C
B-1533
Other strains
High
Low
Fructose 6-phosphate
Glucose 6-phosphate
2-C-methyl-D-erythritol 2,4-cyclic diphosphate
2-C-methyl-D-erythritol 2,4-cyclic diphosphate
Aconitic acid
Ureidosuccinic acid
Phosphoric acid (3TMS)
Glycosyl glyceride peak 1 (6TMS)
Sedoheptulose 7-phosphate
Arginosuccinate
RI2304 Sugar
Uridine-diphosphate-N-acetylglucosamine
Glycerophosphoric acid
Malate
α,α-D-(+)-Trehalose (8TMS)
2 3 4 5 6
VIP scores
D
-log10(p-value)
log2(FC)
Fold Change
4
3
2
Nonadecan-1-ol_1TMS
2C-methyl-D-erythritol 2,4-cyclodiphosphate/2-C-methyl-D-erythritol 2,4-cyclic diphosphate
2-C-methyl-D-erythritol 2,4-cyclic diphosphate
Glucose__1H_1MeOX,_5TMS
aconitic acid
Isocitric_acid_4TMS
Glucose_2L_1MEOX_5TMS
Digalacturonic acid
Glucose-6-phosphate_2L_1MEOX,_6TMS
Fructose-6-phosphate_1MEOX,_6TMS
Glucose-6-phosphate_1H_1MEOX,_6TMS
ureidosuccinic acid
alpha-Hydroxyglutaric_acid_3TMS
fructose 6-phosphate
glucose 6-phosphate
2-keto-3-deoxy-6-phosphogluconate
RI2124_Sugar
ribulose-5-phosphate/xylulose-5-phosphate
RI1016_Unknown
RI2247_C5-C6-Sugar-phosphate
thymidine-5'-diphosphate
2-deoxyribose 5-phosphate.1
3-dehydroshikimic-acid
2-C-methylerythritol 4-phosphate
E
Aconitic acid (LC-MS)
Abundance (detector signals)
6.0E6 5.0E6 4.0E6 3.0E6 2.0E6 1.0E6 0
S t-test p<0.001
W t-test p 0.005
U-test p 0.001
B-1533
Other strains
2C-methyl-D-erythritol (LC-MS)
1.2E7 1.0E7 8.0E6 6.0E6 4.0E6 2.0E6 0
S t-test p<0.001
W t-test p 0.003
U-test p 0.001
B-1533
Other strains
Nonadecan-1-ol (1TMS)
4.0E4 3.0E4 2.0E4 1.0E4 0
S t-test p<0.001
W t-test p<0.001
U-test p 0.001
B-1533
Other strains

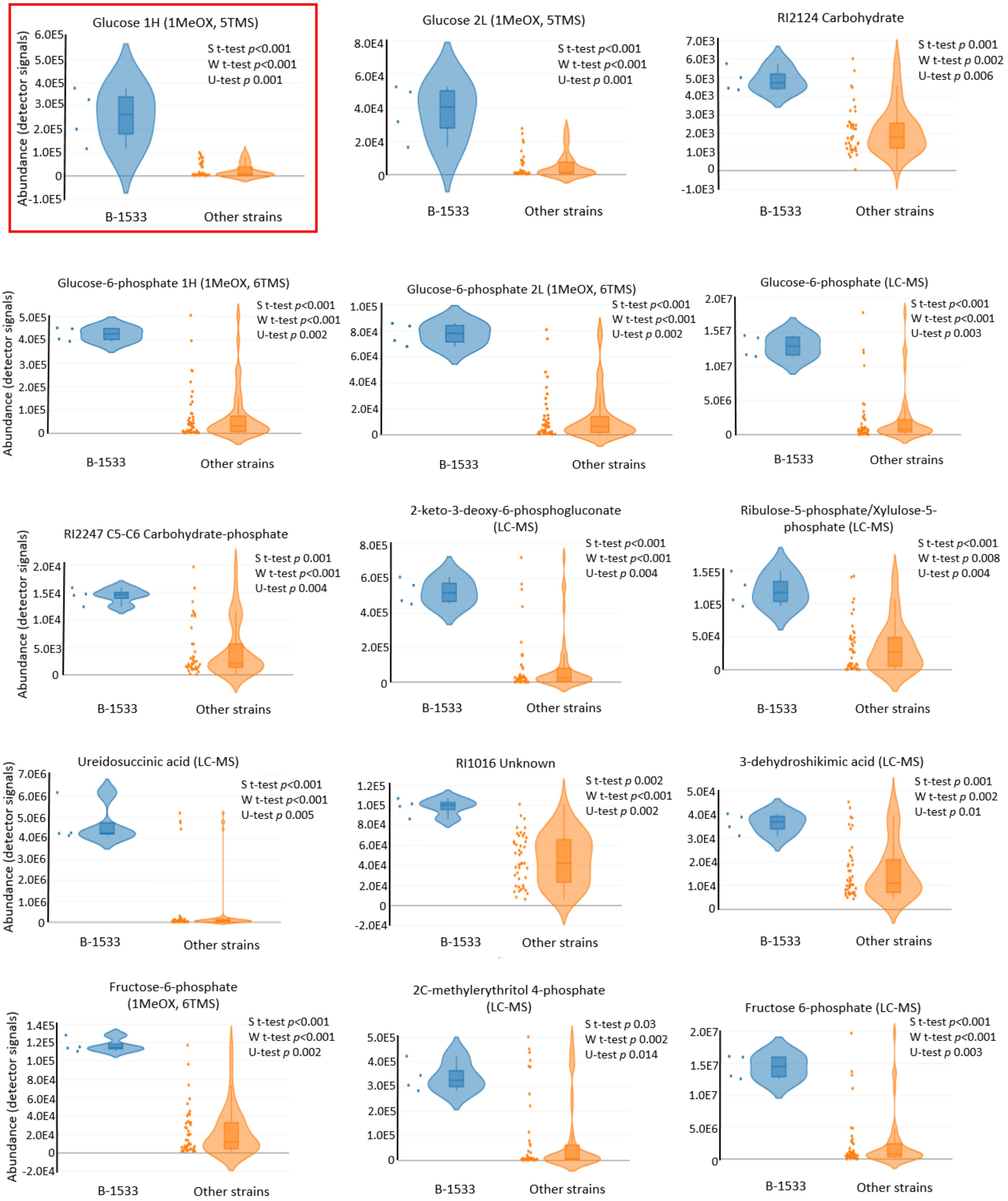

Glucose 1H (1MeOX, 5TMS)
S t-test p<0.001
W t-test p<0.001
U-test p 0.001
Abundance (detector signals)
6.0E5
5.0E5
4.0E5
3.0E5
2.0E5
1.0E5
0
-1.0E5
B-1533
Other strains
Glucose 2L (1MeOX, 5TMS)
S t-test p<0.001
W t-test p<0.001
U-test p 0.001
8.0E4
6.0E4
4.0E4
2.0E4
0
B-1533
Other strains
RI2124 Carbohydrate
S t-test p 0.001
W t-test p 0.002
U-test p 0.006
7.0E3
6.0E3
5.0E3
4.0E3
3.0E3
2.0E3
1.0E3
0
-1.0E3
B-1533
Other strains
Glucose-6-phosphate 1H (1MeOX, 6TMS)
S t-test p<0.001
W t-test p<0.001
U-test p 0.002
Abundance (detector signals)
5.0E5
4.0E5
3.0E5
2.0E5
1.0E5
0
B-1533
Other strains
Glucose-6-phosphate 2L (1MeOX, 6TMS)
S t-test p<0.001
W t-test p<0.001
U-test p 0.002
1.0E5
8.0E4
6.0E4
4.0E4
2.0E4
0
B-1533
Other strains
Glucose-6-phosphate (LC-MS)
S t-test p<0.001
W t-test p<0.001
U-test p 0.003
2.0E7
1.5E7
1.0E7
5.0E6
0
B-1533
Other strains
RI2247 C5-C6 Carbohydrate-phosphate
S t-test p 0.001
W t-test p<0.001
U-test p 0.004
Abundance (detector signals)
2.0E4
1.5E4
1.0E4
5.0E3
0
B-1533
Other strains
2-keto-3-deoxy-6-phosphogluconate (LC-MS)
S t-test p<0.001
W t-test p<0.001
U-test p 0.004
8.0E5
6.0E5
4.0E5
2.0E5
0
B-1533
Other strains
Ribulose-5-phosphate/Xylulose-5-phosphate (LC-MS)
S t-test p<0.001
W t-test p 0.008
U-test p 0.004
1.5E5
1.0E5
5.0E4
0
B-1533
Other strains
Ureidosuccinic acid (LC-MS)
S t-test p<0.001
W t-test p<0.001
U-test p 0.005
Abundance (detector signals)
7.0E6
6.0E6
5.0E6
4.0E6
3.0E6
2.0E6
1.0E6
0
B-1533
Other strains
RI1016 Unknown
S t-test p 0.002
W t-test p<0.001
U-test p 0.002
1.2E5
1.0E5
8.0E4
6.0E4
4.0E4
2.0E4
0
-2.0E4
B-1533
Other strains
3-dehydroshikimic acid (LC-MS)
S t-test p 0.001
W t-test p 0.002
U-test p 0.01
5.0E4
4.0E4
3.0E4
2.0E4
1.0E4
0
B-1533
Other strains
Fructose-6-phosphate (1MeOX, 6TMS)
S t-test p<0.001
W t-test p<0.001
U-test p 0.002
Abundance (detector signals)
1.4E5
1.2E5
1.0E5
8.0E4
6.0E4
4.0E4
2.0E4
0
-2.0E4
B-1533
Other strains
2C-methylerythritol 4-phosphate (LC-MS)
S t-test p 0.03
W t-test p 0.002
U-test p 0.014
5.0E5
4.0E5
3.0E5
2.0E5
1.0E5
0
B-1533
Other strains
Fructose 6-phosphate (LC-MS)
S t-test p<0.001
W t-test p<0.001
U-test p 0.003
2.0E7
1.5E7
1.0E7
5.0E6
0
B-1533
Other strains

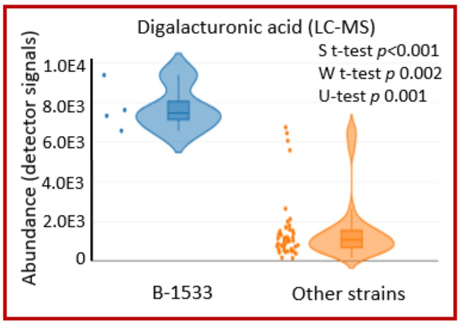

Figure S2-5 Comprehensive statistical analysis of polar metabolite profiles of the extremophilic cyanobacteria strain *Anabaena* cf. pirinica B-1533 (low temperature tolerant). This analysis was performed by comparing the metabolite profiles of this strain with the metabolomes of all other cyanobacterial strains studied. PCA (A) and PLS-DA (B) score plots built for the first two principal components, (C) – VIP scores plot based on PLS-DA shows 15 metabolites contributing most to the difference between the compared groups, (D) – Volcano plot illustrates (colored dots) metabolites with statistically significant (t-test, *p*-value ≤0.05, FDR-corrected) difference in relative content between the compared groups (FC ≥2), (E) – Box plots built for the the strain-specific metabolites validated additionaly by Welch's t-test (W test *p*-value ≤0.05) and Mann-Whitney U-test (U test *p*-value ≤0.01). The red frame indicates metabolites displaying the most significant difference (S t-test *p*-value (FDR adjusted) <0.01, Welch t-test *p*-value <0.01, U-test *p*-value<0.001) in the content between B-1533 and other strains.

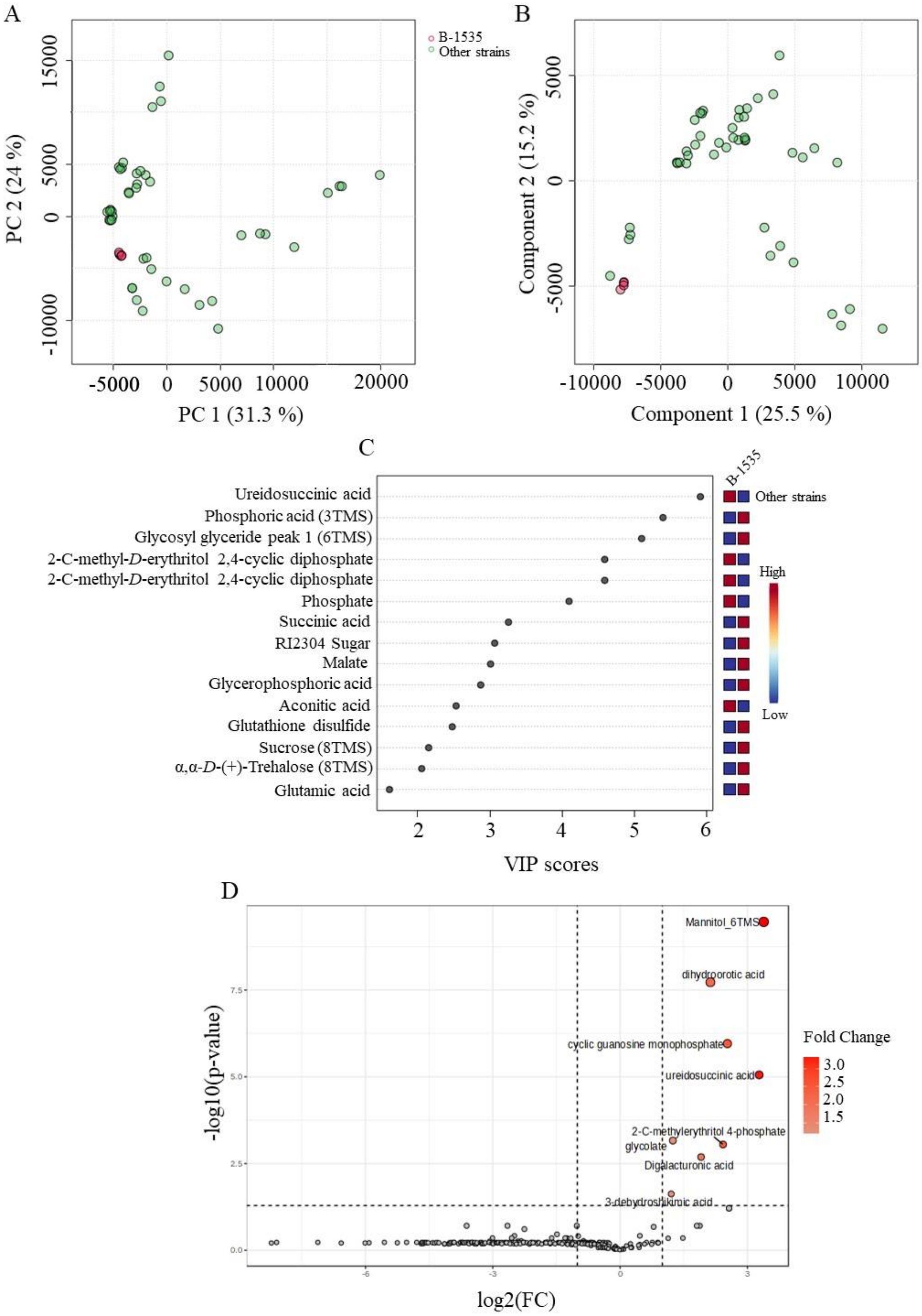

A
B-1535
Other strains
PC 2 (24 %)
PC 1 (31.3 %)
B
Component 2 (15.2 %)
Component 1 (25.5 %)
C
B-1535
Other strains
Ureidosuccinic acid
Phosphoric acid (3TMS)
Glycosyl glyceride peak 1 (6TMS)
2-C-methyl-D-erythritol 2,4-cyclic diphosphate
2-C-methyl-D-erythritol 2,4-cyclic diphosphate
Phosphate
Succinic acid
RI2304 Sugar
Malate
Glycerophosphoric acid
Aconitic acid
Glutathione disulfide
Sucrose (8TMS)
α,α-D-(+)-Trehalose (8TMS)
Glutamic acid
High
Low
VIP scores
D
Mannitol_6TMS
dihydroorotic acid
cyclic guanosine monophosphate
ureidosuccinic acid
2-C-methylerythritol 4-phosphate
glycolate
Digalacturonic acid
3-dehydroshikimic acid
Fold Change
-log10(p-value)
log2(FC)

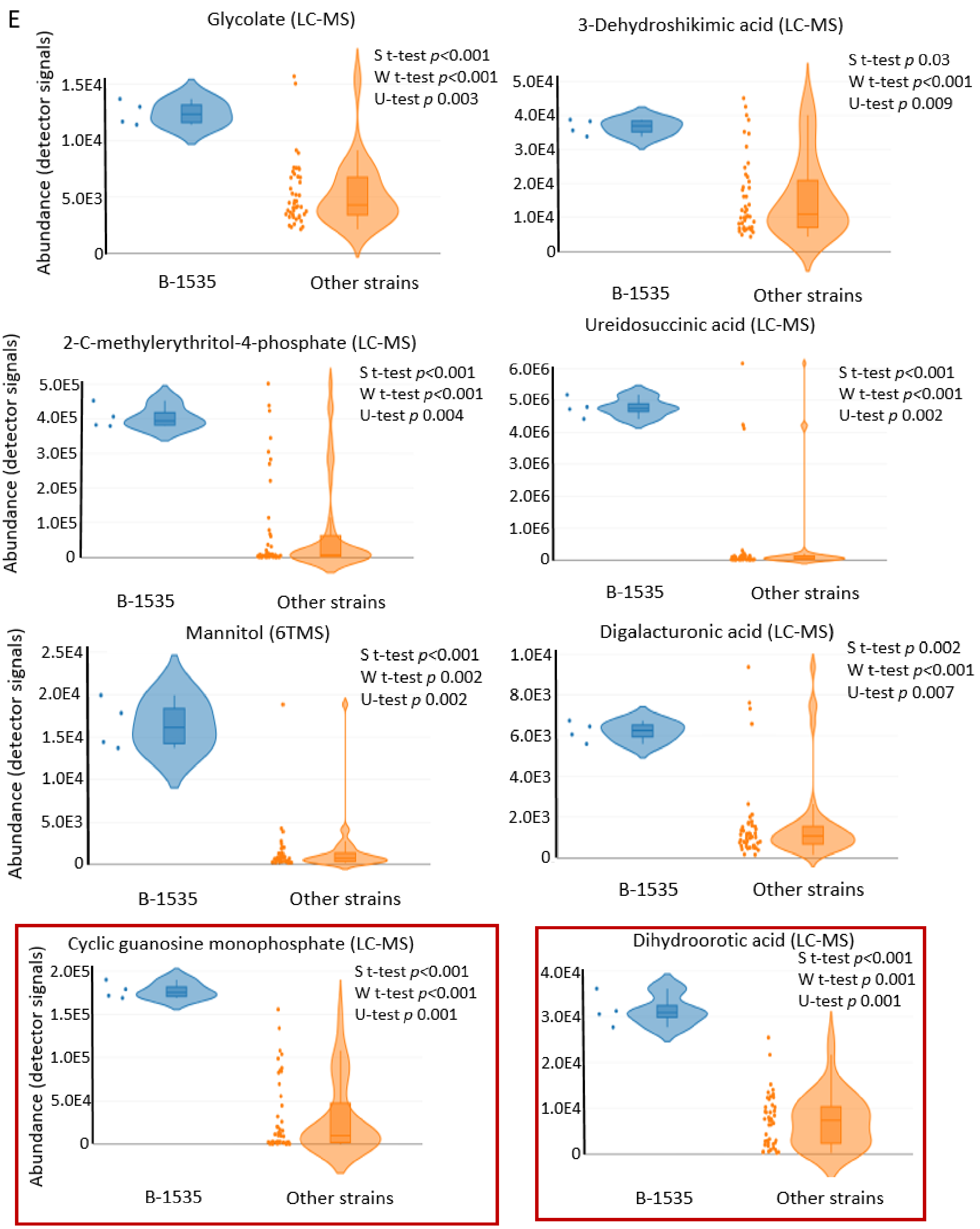

Figure S2-6 Comprehensive statistical analysis of polar metabolite profiles of the extremophilic cyanobacteria strain *Anabaena* sp. B-1535 (low temperature tolerant). This analysis was performed by comparing the metabolite profiles of this strain with the metabolomes of all other cyanobacterial strains studied. PCA (A) and PLS-DA (B) score plots built for the first two principal components, (C) – VIP scores plot based on PLS-DA shows 15 metabolites contributing most to the difference between the compared groups, (D) – Volcano plot illustrates (colored dots) metabolites with statistically significant (t-test, *p*-value ≤0.05, FDR-corrected) difference in relative content between the compared groups (FC ≥2), (E) – Box plots built for the the strain-specific metabolites validated additionally by Welch's t-test (W test *p*-value ≤0.05) and Mann-Whitney U-test (U test *p*-value ≤0.01). The red frame indicates two metabolites displaying the most significant difference (S t-test *p*-value (FDR adjusted) <0.01, Welch t-test *p*-value <0.01, U-test *p*-value<0.001) in the content between B-1535 and other strains.

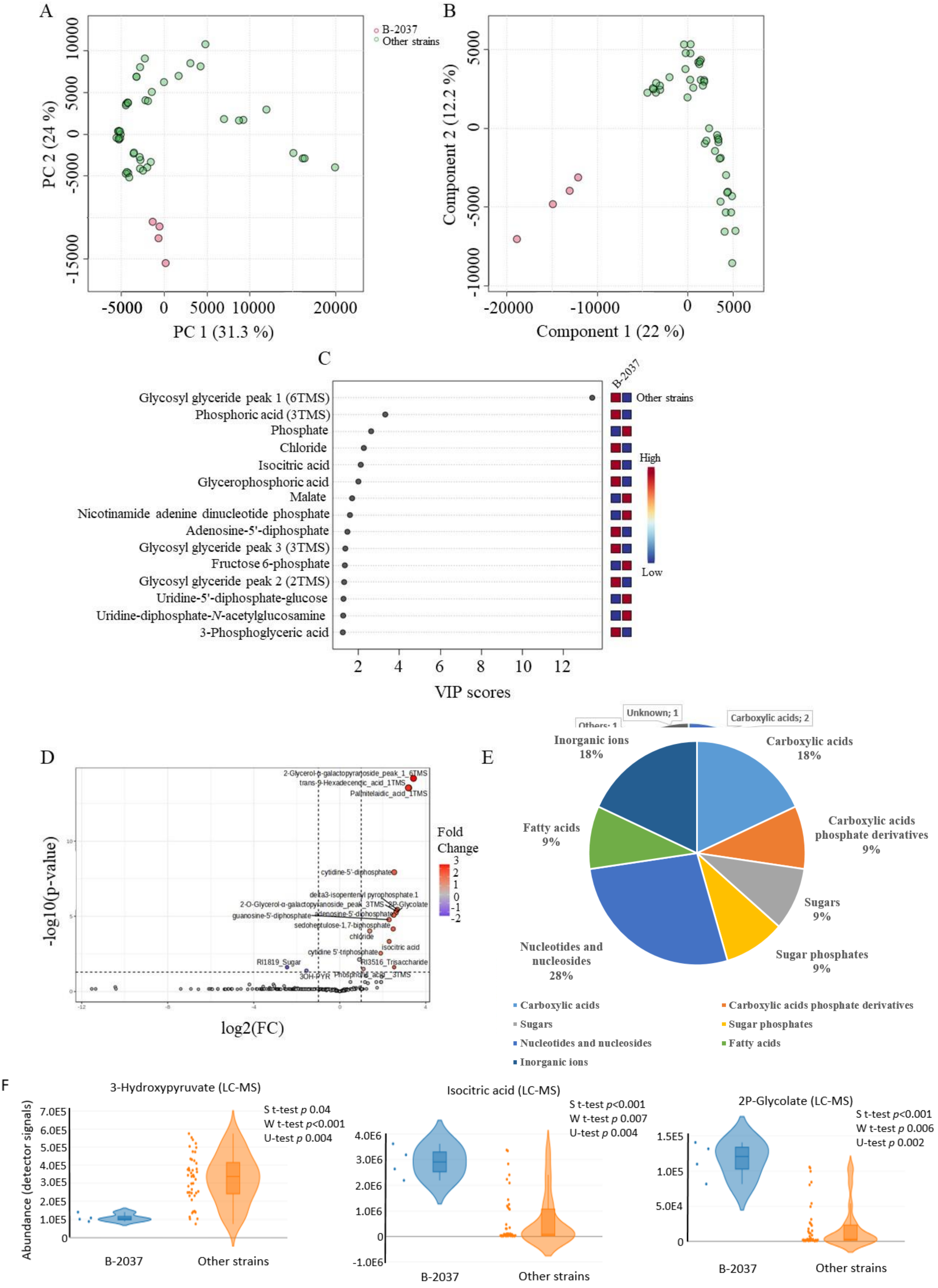
A
B-2037
Other strains
PC 2 (24 %)
PC 1 (31.3 %)
B
Component 2 (12.2 %)
Component 1 (22 %)
C
Glycosyl glyceride peak 1 (6TMS)
Phosphoric acid (3TMS)
Phosphate
Chloride
Isocitric acid
Glycerophosphoric acid
Malate
Nicotinamide adenine dinucleotide phosphate
Adenosine-5'-diphosphate
Glycosyl glyceride peak 3 (3TMS)
Fructose 6-phosphate
Glycosyl glyceride peak 2 (2TMS)
Uridine-5'-diphosphate-glucose
Uridine-diphosphate-N-acetylglucosamine
3-Phosphoglyceric acid
B-2037
Other strains
High
Low
VIP scores
D
-log10(p-value)
log2(FC)
Fold Change
2-Glycerol-α-galactopyranoside_peak_1_6TMS
trans-9-Hexadecenoic_acid_1TMS
Palmitelaidic_acid_1TMS
cytidine-5'-diphosphate
delta3-isopentenyl pyrophosphate.1
2-O-Glycerol-α-galactopyranoside_peak_3TMS
2P-Glycolate
guanosine-5'-diphosphate
adenosine-5'-diphosphate
sedoheptulose-1,7-biphosphate
chloride
isocitric acid
cytidine 5'-triphosphate
RI1819_Sugar
RI3516_Trisaccharide
3OH-PYR
E
Unknown; 1
Others; 1
Carboxylic acids; 2
Inorganic ions 18%
Carboxylic acids 18%
Fatty acids 9%
Carboxylic acids phosphate derivatives 9%
Sugars 9%
Sugar phosphates 9%
Nucleotides and nucleosides 28%
Carboxylic acids
Carboxylic acids phosphate derivatives
Sugars
Sugar phosphates
Nucleotides and nucleosides
Fatty acids
Inorganic ions
F
3-Hydroxypyruvate (LC-MS)
S t-test p 0.04
W t-test p<0.001
U-test p 0.004
Abundance (detector signals)
B-2037
Other strains
Isocitric acid (LC-MS)
S t-test p<0.001
W t-test p 0.007
U-test p 0.004
2P-Glycolate (LC-MS)
S t-test p<0.001
W t-test p 0.006
U-test p 0.002

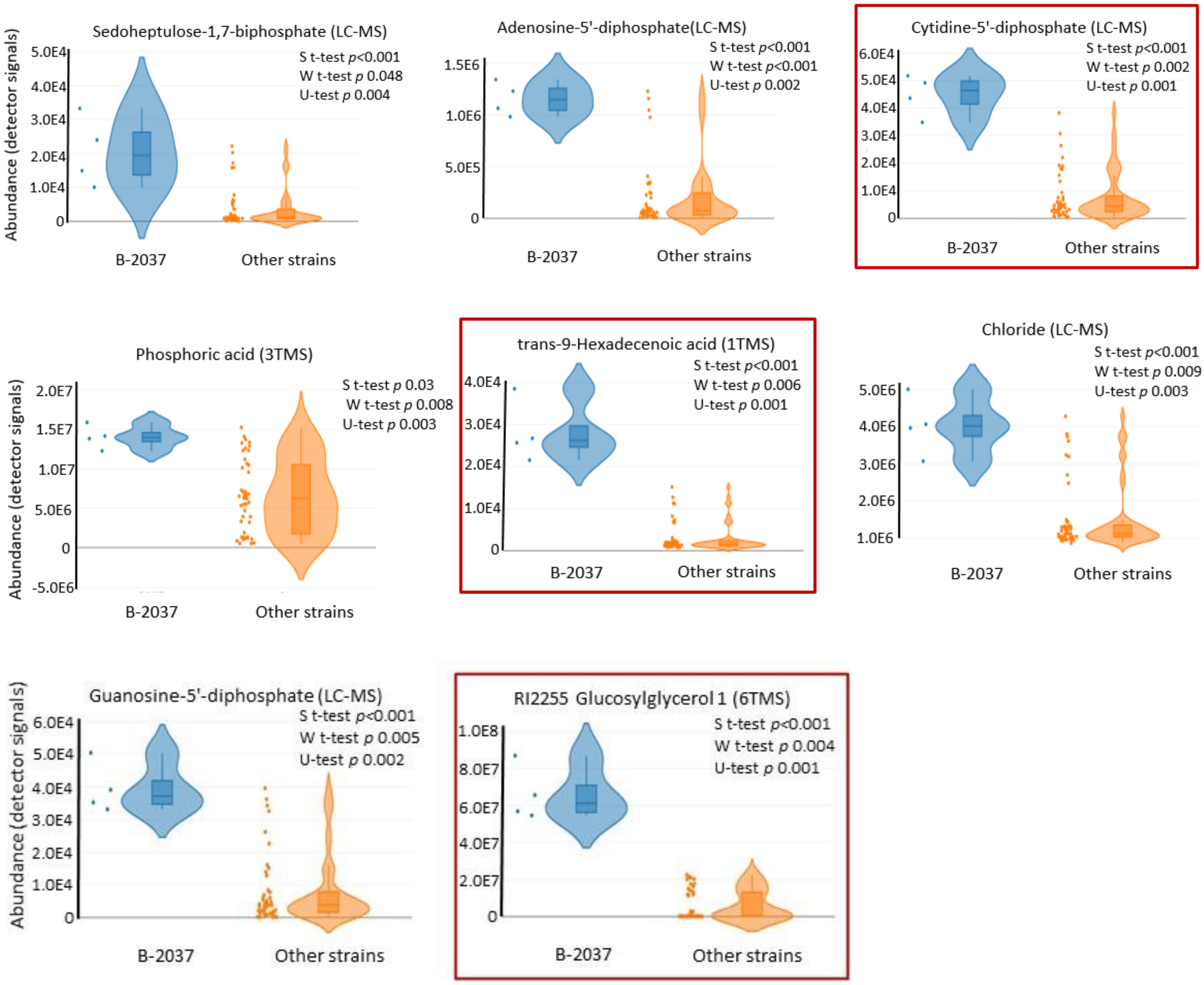

Figure S2-7 Comprehensive statistical analysis of polar metabolite profiles of the extremophilic cyanobacteria strain *Sodalinema orleanskyi* B-2037 (haloalkaliphilic and natronophilic). This analysis was performed by comparing the metabolite profiles of this strain with the metabolomes of all other cyanobacterial strains studied. PCA (A) and PLS-DA (B) score plots built for the first two principal components, (C) – VIP scores plot based on PLS-DA shows 15 metabolites contributing most to the difference between the compared groups, (D) – Volcano plot illustrates (colored dots) metabolites with statistically significant (t-test, *p*-value ≤0.05, FDR-corrected) difference in relative content between the compared groups (FC ≥2), (E) – Vend diagram presenting the distribution of the strain-specific metabolites, according to their belonging to specific chemical classes, (F) – Box plots built for the the strain-specific metabolites validated additionally by Welch's t-test (W test *p*-value ≤0.05) and Mann-Whitney U-test (U test *p*-value ≤0.01). The red frame indicates three metabolites displaying the most significant difference (S t-test *p*-value (FDR adjusted) <0.01, Welch t-test *p*-value <0.01, U-test *p*-value<0.001) in the content between B-2037 and other strains.

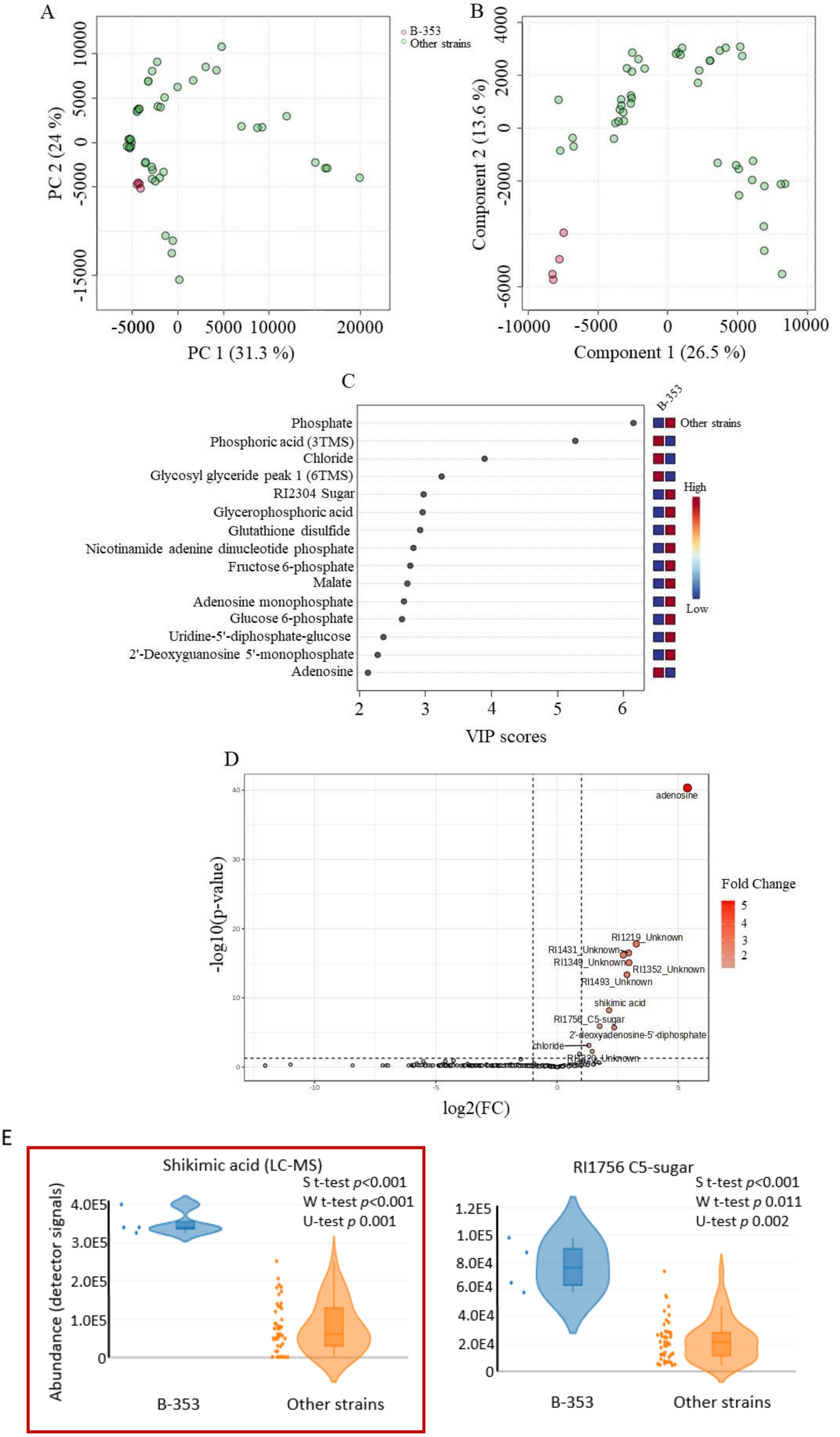

A
B-353
Other strains
PC 1 (31.3 %)
PC 2 (24 %)
B
Component 1 (26.5 %)
Component 2 (13.6 %)
C
B-353
Other strains
Phosphate
Phosphoric acid (3TMS)
Chloride
Glycosyl glyceride peak 1 (6TMS)
RI2304 Sugar
Glycerophosphoric acid
Glutathione disulfide
Nicotinamide adenine dinucleotide phosphate
Fructose 6-phosphate
Malate
Adenosine monophosphate
Glucose 6-phosphate
Uridine-5'-diphosphate-glucose
2'-Deoxyguanosine 5'-monophosphate
Adenosine
High
Low
VIP scores
D
adenosine
RI1219_Unknown
RI1431_Unknown
RI1349_Unknown
RI1352_Unknown
RI1493_Unknown
shikimic acid
RI1756_C5-sugar
2'-deoxyadenosine-5'-diphosphate
chloride
Fold Change
-log10(p-value)
log2(FC)
E
Shikimic acid (LC-MS)
S t-test p<0.001
W t-test p<0.001
U-test p 0.001
Abundance (detector signals)
B-353
Other strains
RI1756 C5-sugar
S t-test p<0.001
W t-test p 0.011
U-test p 0.002
B-353
Other strains

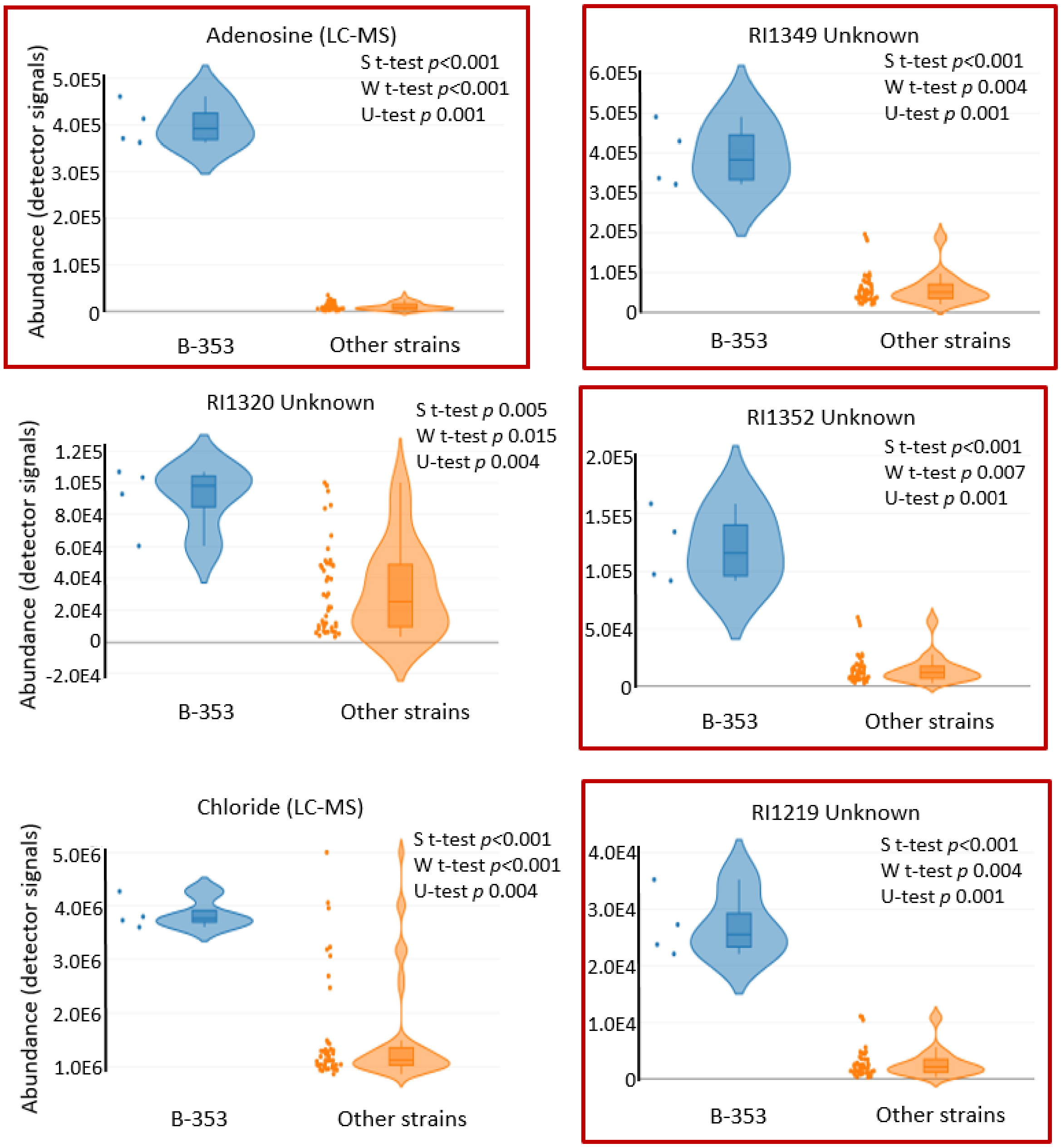

Figure S2-8 Comprehensive statistical analysis of polar metabolite profiles of the extremophilic cyanobacteria strain *Sodalinema gerasimenkoae* B-353 (haloalkaliphilic and natronophilic). This analysis was performed by comparing the metabolite profiles of this strain with the metabolomes of all other cyanobacterial strains studied. PCA (A) and PLS-DA (B) score plots built for the first two principal components, (C) – VIP scores plot based on PLS-DA shows 15 metabolites contributing most to the difference between the compared groups, (D) – Volcano plot illustrates (colored dots) metabolites with statistically significant (t-test, *p*-value ≤0.05, FDR-corrected) difference in relative content between the compared groups (FC ≥2), (E) – Box plots built for the strain-specific metabolites validated additionally by Welch's t-test (W test *p*-value ≤0.05) and Mann-Whitney U-test (U test *p*-value ≤0.01). The red frame indicates five metabolites displaying the most significant difference (S t-test *p*-value (FDR adjusted) <0.01, Welch t-test *p*-value <0.01, U-test *p*-value<0.001) in the content between B-353 and other strains.

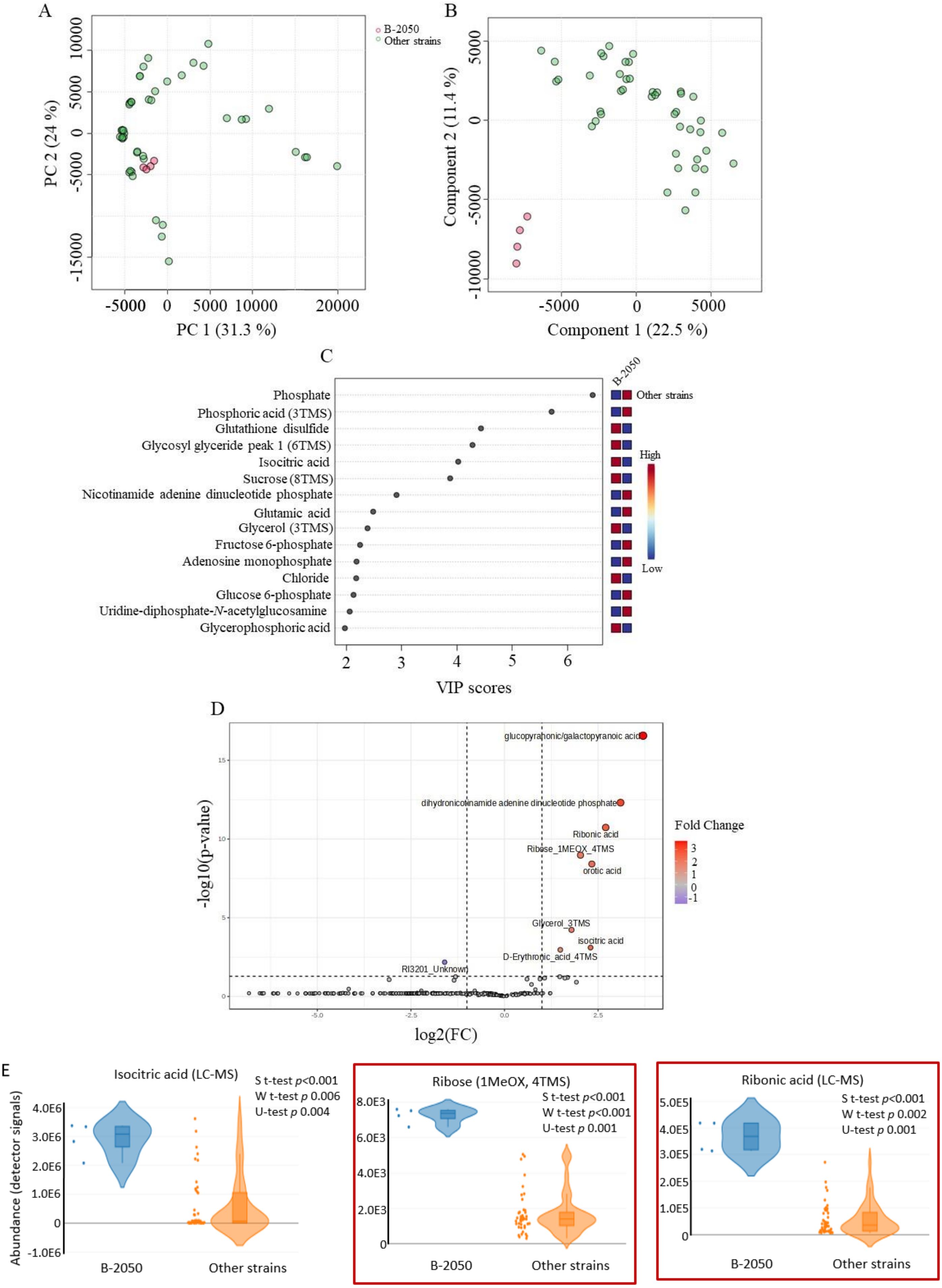
A
B-2050
Other strains
PC 1 (31.3 %)
PC 2 (24 %)
B
Component 1 (22.5 %)
Component 2 (11.4 %)
C
B-2050
Other strains
Phosphate
Phosphoric acid (3TMS)
Glutathione disulfide
Glycosyl glyceride peak 1 (6TMS)
Isocitric acid
Sucrose (8TMS)
Nicotinamide adenine dinucleotide phosphate
Glutamic acid
Glycerol (3TMS)
Fructose 6-phosphate
Adenosine monophosphate
Chloride
Glucose 6-phosphate
Uridine-diphosphate-N-acetylglucosamine
Glycerophosphoric acid
High
Low
VIP scores
D
glucopyranonic/galactopyranoic acid
dihydronicotinamide adenine dinucleotide phosphate
Ribonic acid
Ribose_1MEOX_4TMS
orotic acid
Glycerol_3TMS
isocitric acid
D-Erythronic_acid_4TMS
RI3201_Unknown
Fold Change
-log10(p-value)
log2(FC)
E
Isocitric acid (LC-MS)
S t-test p<0.001
W t-test p 0.006
U-test p 0.004
Abundance (detector signals)
B-2050
Other strains
Ribose (1MeOX, 4TMS)
S t-test p<0.001
W t-test p<0.001
U-test p 0.001
Ribonic acid (LC-MS)
S t-test p<0.001
W t-test p 0.002
U-test p 0.001

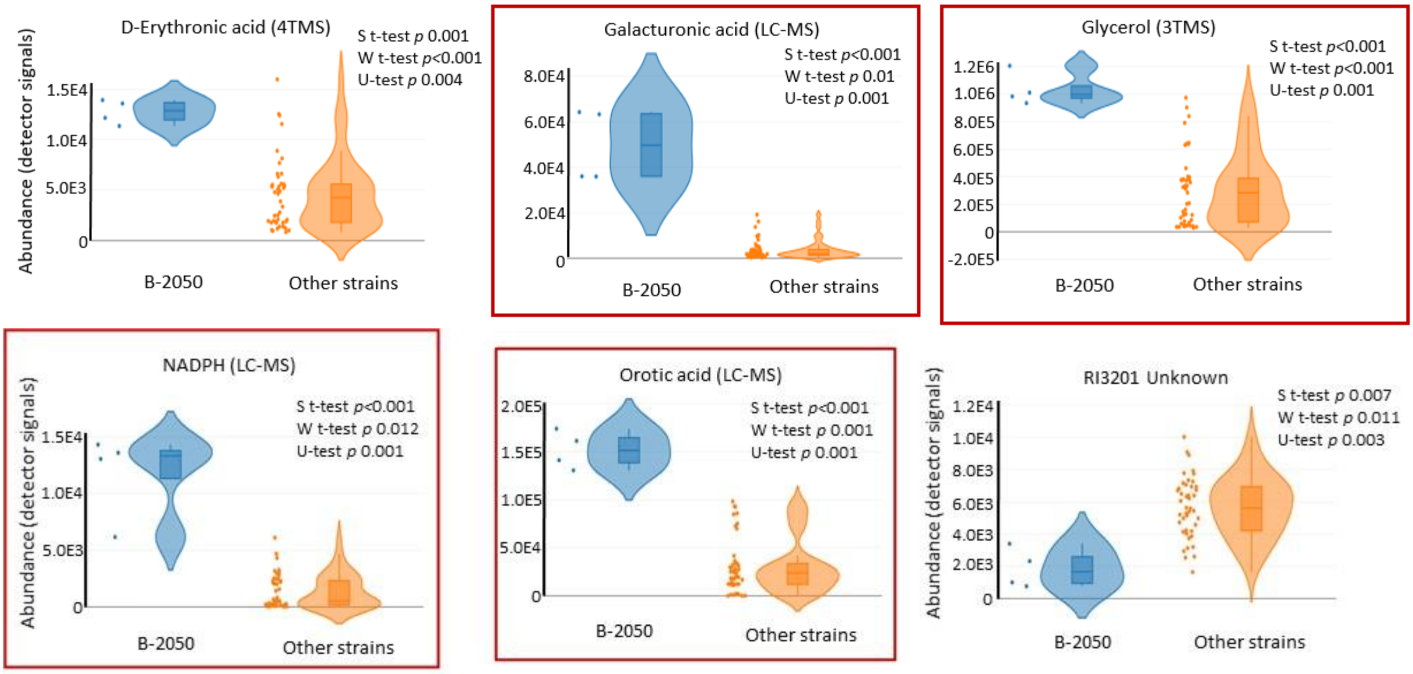

Figure S2-9 Comprehensive statistical analysis of polar metabolite profiles of the extremophilic cyanobacteria strain *Sodalinema stalii* B-2050 (halophilic). This analysis was performed by comparing the metabolite profiles of this strain with the metabolomes of all other cyanobacterial strains studied. PCA (A) and PLS-DA (B) score plots built for the first two principal components, (C) – VIP scores plot based on PLS-DA shows 15 metabolites contributing most to the difference between the compared groups, (D) – Volcano plot illustrates (colored dots) metabolites with statistically significant (t-test, *p*-value ≤0.05, FDR-corrected) difference in relative content between the compared groups (FC ≥2), (E) – Box plots built for the strain-specific metabolites validated additionally by Welch's t-test (W test *p*-value ≤0.05) and Mann-Whitney U-test (U test *p*-value ≤0.01). The red frame indicates six metabolites displaying the most significant difference (S t-test *p*-value (FDR adjusted) <0.01, Welch t-test *p*-value <0.01, U-test *p*-value <0.001) in the content between B-2050 and other strains.

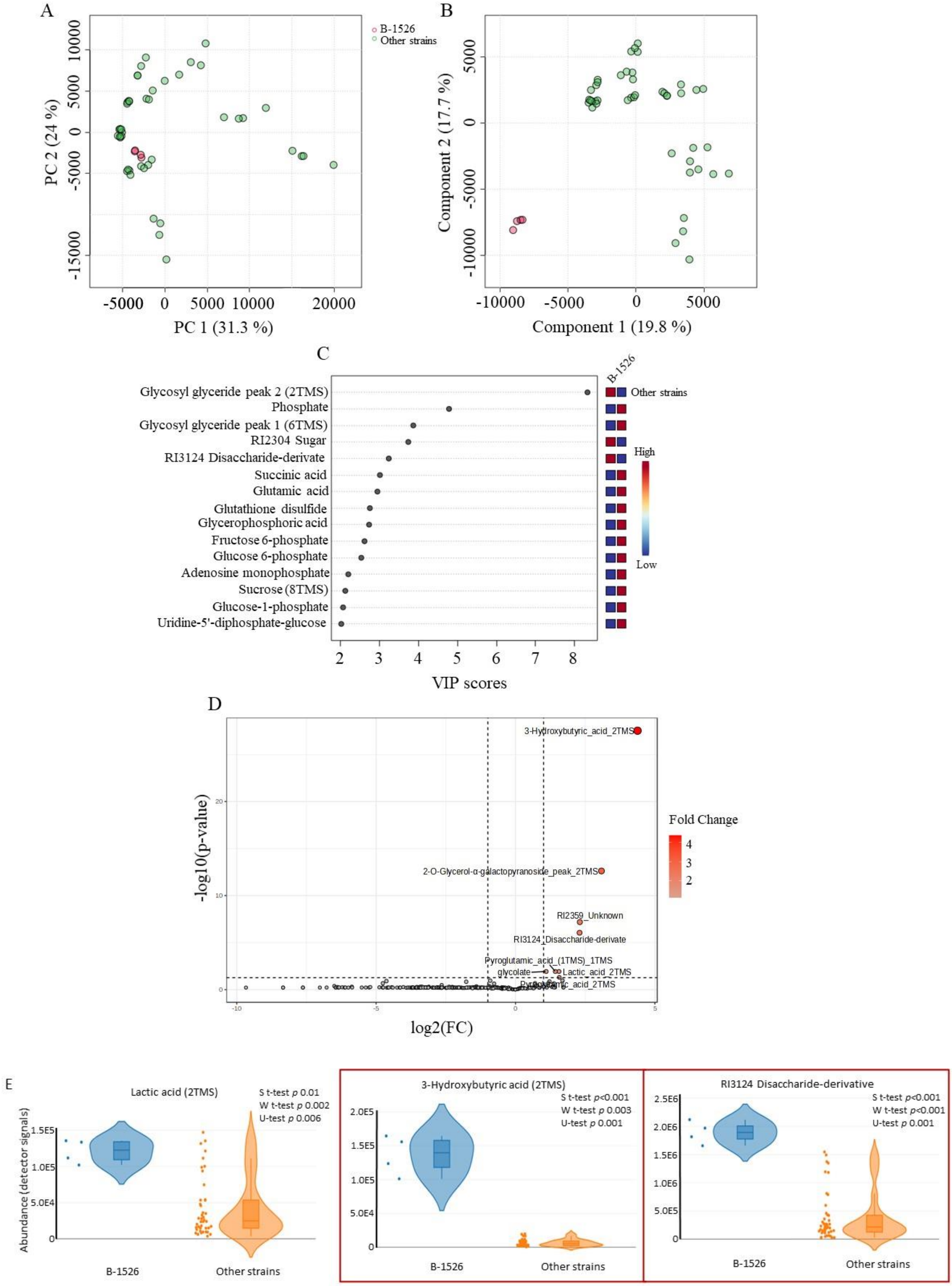
A
B-1526
Other strains
PC 2 (24 %)
PC 1 (31.3 %)
B
Component 2 (17.7 %)
Component 1 (19.8 %)
C
Glycosyl glyceride peak 2 (2TMS)
Phosphate
Glycosyl glyceride peak 1 (6TMS)
RI2304 Sugar
RI3124 Disaccharide-derivate
Succinic acid
Glutamic acid
Glutathione disulfide
Glycerophosphoric acid
Fructose 6-phosphate
Glucose 6-phosphate
Adenosine monophosphate
Sucrose (8TMS)
Glucose-1-phosphate
Uridine-5'-diphosphate-glucose
VIP scores
B-1526
Other strains
High
Low
D
3-Hydroxybutyric_acid_2TMS
2-O-Glycerol-α-galactopyranoside_peak_2TMS
RI2359_Unknown
RI3124_Disaccharide-derivate
Pyroglutamic_acid_(1TMS)_1TMS
glycolate
Lactic_acid_2TMS
-log10(p-value)
log2(FC)
Fold Change
E
Lactic acid (2TMS)
S t-test p 0.01
W t-test p 0.002
U-test p 0.006
Abundance (detector signals)
B-1526
Other strains
3-Hydroxybutyric acid (2TMS)
S t-test p<0.001
W t-test p 0.003
U-test p 0.001
B-1526
Other strains
RI3124 Disaccharide-derivative
S t-test p<0.001
W t-test p<0.001
U-test p 0.001
B-1526
Other strains

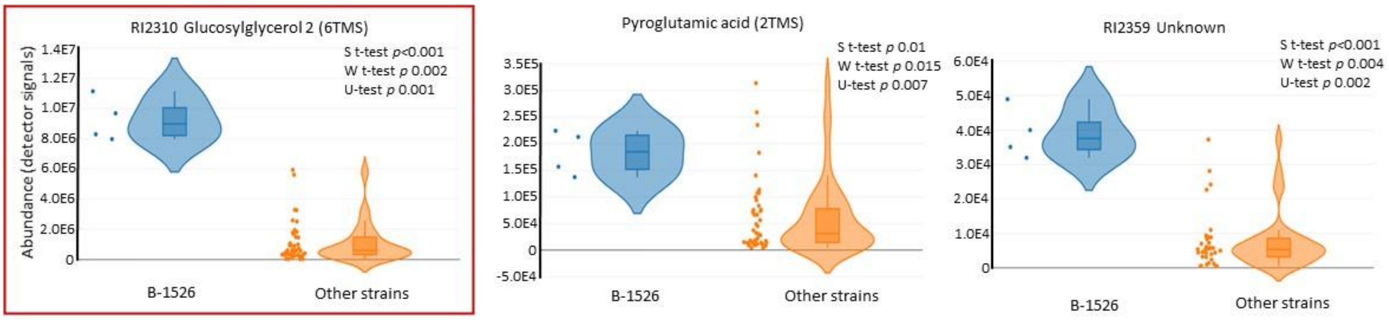

Figure S2-10 Comprehensive statistical analysis of polar metabolite profiles of the extremophilic cyanobacteria strain *Limnospira* sp. B-1526 (haloalkaliphilic and natronophilic). This analysis was performed by comparing the metabolite profiles of this strain with the metabolomes of all other cyanobacterial strains studied. PCA (A) and PLS-DA (B) score plots built for the first two principal components, (C) – VIP scores plot based on PLS-DA shows 15 metabolites contributing most to the difference between the compared groups, (D) – Volcano plot illustrates (colored dots) metabolites with statistically significant (t-test, *p*-value ≤0.05, FDR-corrected) difference in relative content between the compared groups (FC ≥2), (E) – Box plots built for the strain-specific metabolites validated additionally by Welch's t-test (W test *p*-value ≤0.05) and Mann-Whitney U-test (U test *p*-value ≤0.01). The red frame indicates three metabolites displaying the most significant difference (S t-test *p*-value (FDR adjusted) <0.01, Welch t-test *p*-value <0.01, U-test *p*-value<0.001) in the content between B-1526 and other strains.

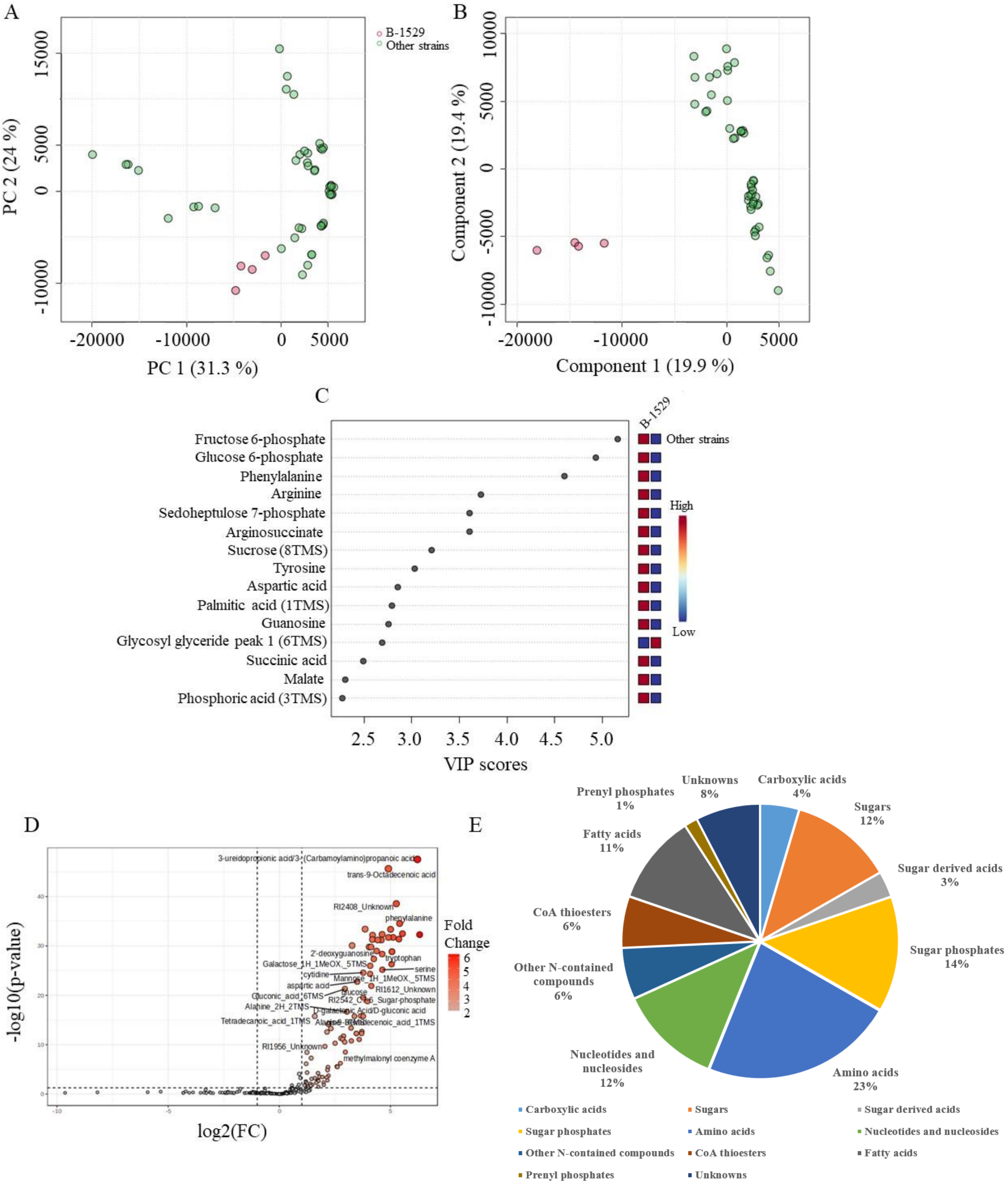
A
B-1529
Other strains
PC 2 (24 %)
PC 1 (31.3 %)
B
Component 2 (19.4 %)
Component 1 (19.9 %)
C
B-1529
Other strains
Fructose 6-phosphate
Glucose 6-phosphate
Phenylalanine
Arginine
Sedoheptulose 7-phosphate
Arginosuccinate
Sucrose (8TMS)
Tyrosine
Aspartic acid
Palmitic acid (1TMS)
Guanosine
Glycosyl glyceride peak 1 (6TMS)
Succinic acid
Malate
Phosphoric acid (3TMS)
High
Low
VIP scores
D
3-ureidopropionic acid/3-(Carbamoylamino)propanoic acid
trans-9-Octadecenoic acid
RI2408_Unknown
phenylalanine
2'-deoxyguanosine
tryptophan
Galactose_1H_1MeOX._5TMS
serine
cytidine
Mannose_1H_1MeOX._5TMS
aspartic acid
RI1612_Unknown
glucose
Gluconic_acid_6TMS
RI2542_C6_Sugar-phosphate
Alanine_2H_2TMS
D-galactonic Acid/D-gluconic acid
Tetradecanoic_acid_1TMS
RI1956_Unknown
methylmalonyl coenzyme A
Fold Change
-log10(p-value)
log2(FC)
E
Unknowns 8%
Carboxylic acids 4%
Prenyl phosphates 1%
Sugars 12%
Fatty acids 11%
Sugar derived acids 3%
CoA thioesters 6%
Sugar phosphates 14%
Other N-contained compounds 6%
Nucleotides and nucleosides 12%
Amino acids 23%
Carboxylic acids
Sugars
Sugar derived acids
Sugar phosphates
Amino acids
Nucleotides and nucleosides
Other N-contained compounds
CoA thioesters
Fatty acids
Prenyl phosphates
Unknowns

F

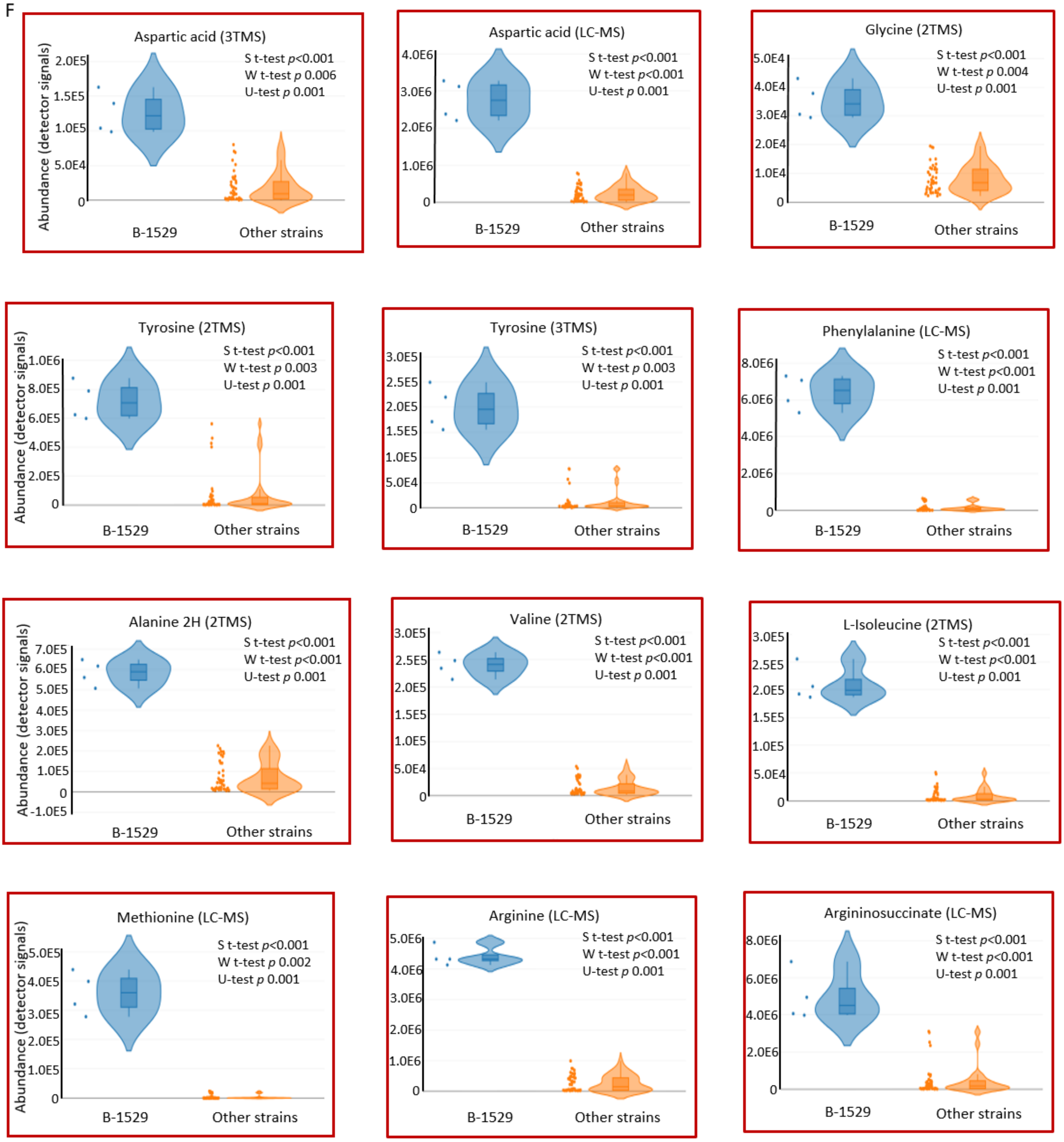
Aspartic acid (3TMS)
Abundance (detector signals)
2.0E5
1.5E5
1.0E5
5.0E4
0
S t-test p<0.001
W t-test p 0.006
U-test p 0.001
B-1529
Other strains
Aspartic acid (LC-MS)
4.0E6
3.0E6
2.0E6
1.0E6
0
S t-test p<0.001
W t-test p<0.001
U-test p 0.001
B-1529
Other strains
Glycine (2TMS)
5.0E4
4.0E4
3.0E4
2.0E4
1.0E4
0
S t-test p<0.001
W t-test p 0.004
U-test p 0.001
B-1529
Other strains
Tyrosine (2TMS)
Abundance (detector signals)
1.0E6
8.0E5
6.0E5
4.0E5
2.0E5
0
S t-test p<0.001
W t-test p 0.003
U-test p 0.001
B-1529
Other strains
Tyrosine (3TMS)
3.0E5
2.5E5
2.0E5
1.5E5
1.0E5
5.0E4
0
S t-test p<0.001
W t-test p 0.003
U-test p 0.001
B-1529
Other strains
Phenylalanine (LC-MS)
8.0E6
6.0E6
4.0E6
2.0E6
0
S t-test p<0.001
W t-test p<0.001
U-test p 0.001
B-1529
Other strains
Alanine 2H (2TMS)
Abundance (detector signals)
7.0E5
6.0E5
5.0E5
4.0E5
3.0E5
2.0E5
1.0E5
0
-1.0E5
S t-test p<0.001
W t-test p<0.001
U-test p 0.001
B-1529
Other strains
Valine (2TMS)
3.0E5
2.5E5
2.0E5
1.5E5
1.0E5
5.0E4
0
S t-test p<0.001
W t-test p<0.001
U-test p 0.001
B-1529
Other strains
L-Isoleucine (2TMS)
3.0E5
2.5E5
2.0E5
1.5E5
1.0E5
5.0E4
0
S t-test p<0.001
W t-test p<0.001
U-test p 0.001
B-1529
Other strains
Methionine (LC-MS)
Abundance (detector signals)
5.0E5
4.0E5
3.0E5
2.0E5
1.0E5
0
S t-test p<0.001
W t-test p 0.002
U-test p 0.001
B-1529
Other strains
Arginine (LC-MS)
5.0E6
4.0E6
3.0E6
2.0E6
1.0E6
0
S t-test p<0.001
W t-test p<0.001
U-test p 0.001
B-1529
Other strains
Argininosuccinate (LC-MS)
8.0E6
6.0E6
4.0E6
2.0E6
0
S t-test p<0.001
W t-test p<0.001
U-test p 0.001
B-1529
Other strains

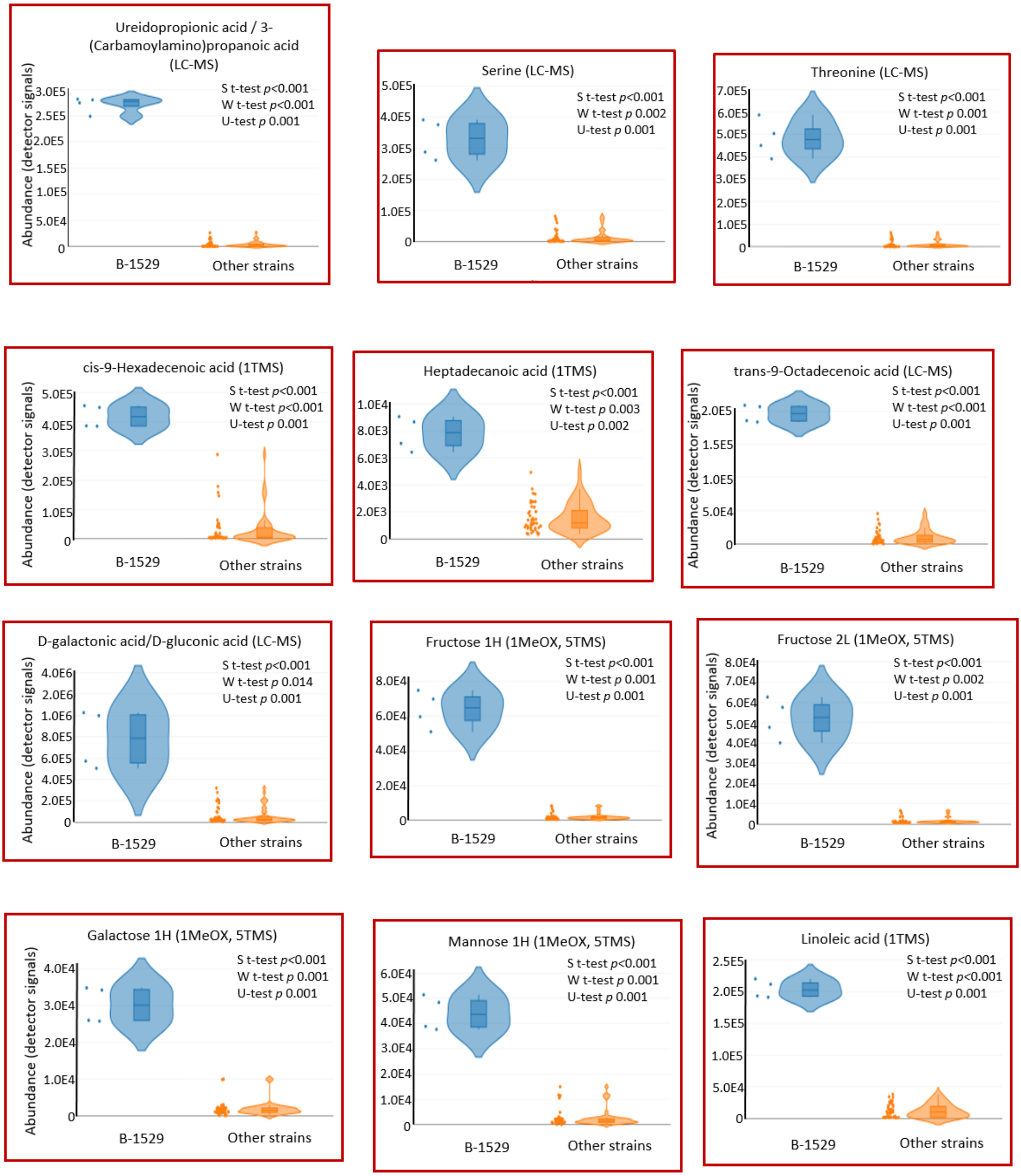

Ureidopropionic acid / 3-(Carbamoylamino)propanoic acid (LC-MS)
Abundance (detector signals)
3.0E5
2.5E5
2.0E5
1.5E5
1.0E5
5.0E4
0
S t-test p<0.001
W t-test p<0.001
U-test p 0.001
B-1529
Other strains
Serine (LC-MS)
5.0E5
4.0E5
3.0E5
2.0E5
1.0E5
0
S t-test p<0.001
W t-test p 0.002
U-test p 0.001
B-1529
Other strains
Threonine (LC-MS)
7.0E5
6.0E5
5.0E5
4.0E5
3.0E5
2.0E5
1.0E5
0
S t-test p<0.001
W t-test p 0.001
U-test p 0.001
B-1529
Other strains
cis-9-Hexadecenoic acid (1TMS)
Abundance (detector signals)
5.0E5
4.0E5
3.0E5
2.0E5
1.0E5
0
S t-test p<0.001
W t-test p<0.001
U-test p 0.001
B-1529
Other strains
Heptadecanoic acid (1TMS)
1.0E4
8.0E3
6.0E3
4.0E3
2.0E3
0
S t-test p<0.001
W t-test p 0.003
U-test p 0.002
B-1529
Other strains
trans-9-Octadecenoic acid (LC-MS)
Abundance (detector signals)
2.0E5
1.5E5
1.0E5
5.0E4
0
S t-test p<0.001
W t-test p<0.001
U-test p 0.001
B-1529
Other strains
D-galactonic acid/D-gluconic acid (LC-MS)
Abundance (detector signals)
4.0E6
2.0E6
1.0E6
8.0E5
6.0E5
4.0E5
2.0E5
0
S t-test p<0.001
W t-test p 0.014
U-test p 0.001
B-1529
Other strains
Fructose 1H (1MeOX, 5TMS)
8.0E4
6.0E4
4.0E4
2.0E4
0
S t-test p<0.001
W t-test p 0.001
U-test p 0.001
B-1529
Other strains
Fructose 2L (1MeOX, 5TMS)
Abundance (detector signals)
8.0E4
7.0E4
6.0E4
5.0E4
4.0E4
3.0E4
2.0E4
1.0E4
0
S t-test p<0.001
W t-test p 0.002
U-test p 0.001
B-1529
Other strains
Galactose 1H (1MeOX, 5TMS)
Abundance (detector signals)
4.0E4
3.0E4
2.0E4
1.0E4
0
S t-test p<0.001
W t-test p 0.001
U-test p 0.001
B-1529
Other strains
Mannose 1H (1MeOX, 5TMS)
6.0E4
5.0E4
4.0E4
3.0E4
2.0E4
1.0E4
0
S t-test p<0.001
W t-test p 0.001
U-test p 0.001
B-1529
Other strains
Linoleic acid (1TMS)
2.5E5
2.0E5
1.5E5
1.0E5
5.0E4
0
S t-test p<0.001
W t-test p<0.001
U-test p 0.001
B-1529
Other strains

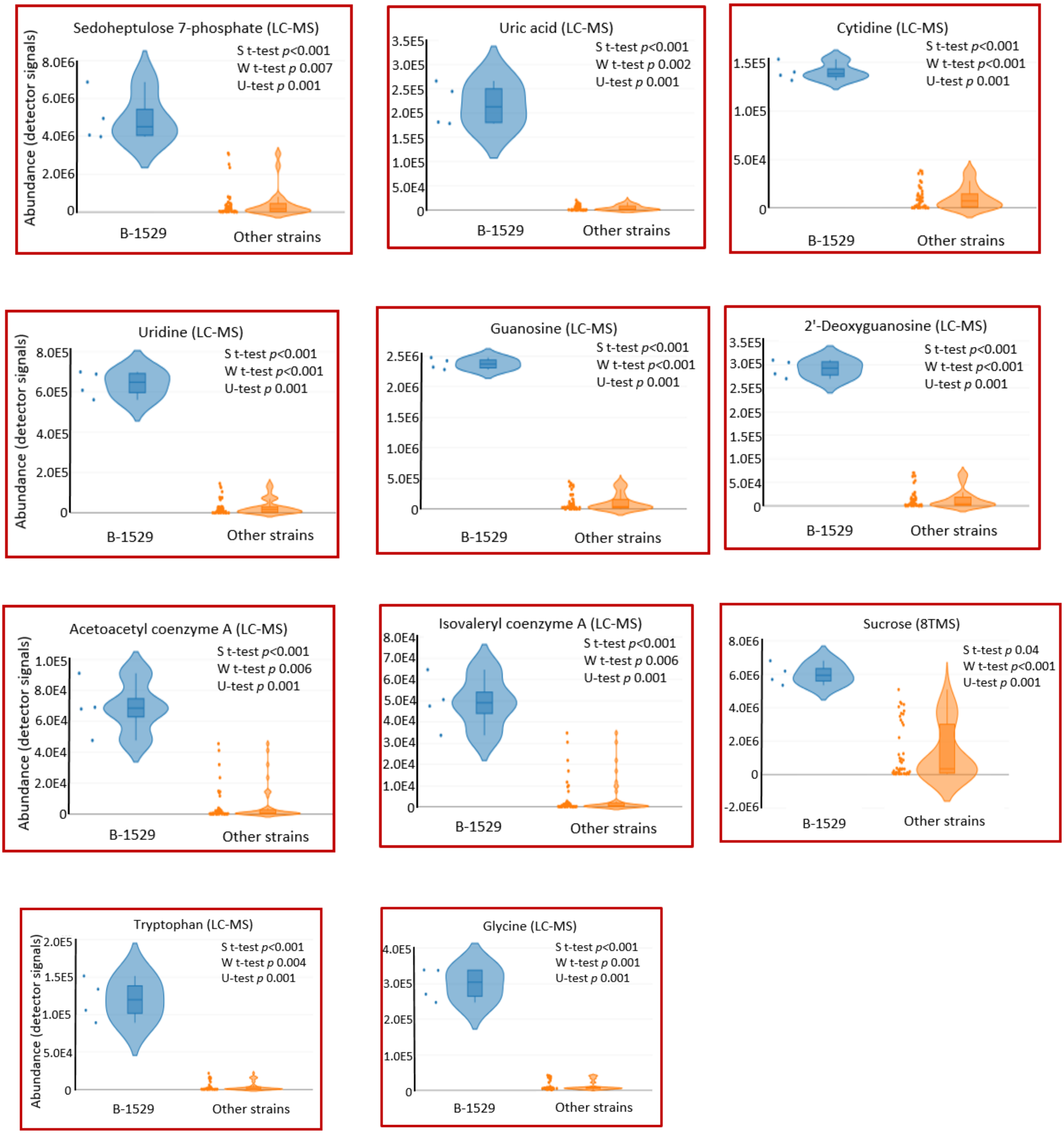

Figure S2-11 Comprehensive statistical analysis of polar metabolite profiles of the extremophilic cyanobacteria strain *Nodularia* sp. B-1529 (haloalkaliphilic and natronophilic). This analysis was performed by comparing the metabolite profiles of this strain with the metabolomes of all other cyanobacterial strains studied. PCA (A) and PLS-DA (B) score plots built for the first two principal components, (C) – VIP scores plot based on PLS-DA shows 15 metabolites contributing most to the difference between the compared groups, (D) – Volcano plot illustrates (colored dots) metabolites with statistically significant (t-test, *p*-value ≤0.05, FDR-corrected) difference in relative content between the compared groups (FC ≥2). (E) – Vend diagram presenting the distribution of metabolites identified by the Volcano plot analysis, according to their belonging to specific chemical classes, (F) – Box plots built for the most strain-specific metabolites validated additionally by Welch's t-test and Mann-Whitney U-test (S t-test *p*-value (FDR adjusted) <0.01, Welch t-test *p*-value <0.01, U-test *p*-value<0.001) and displaying >10-fold difference in the relative abundances between B-1529 and other strains.

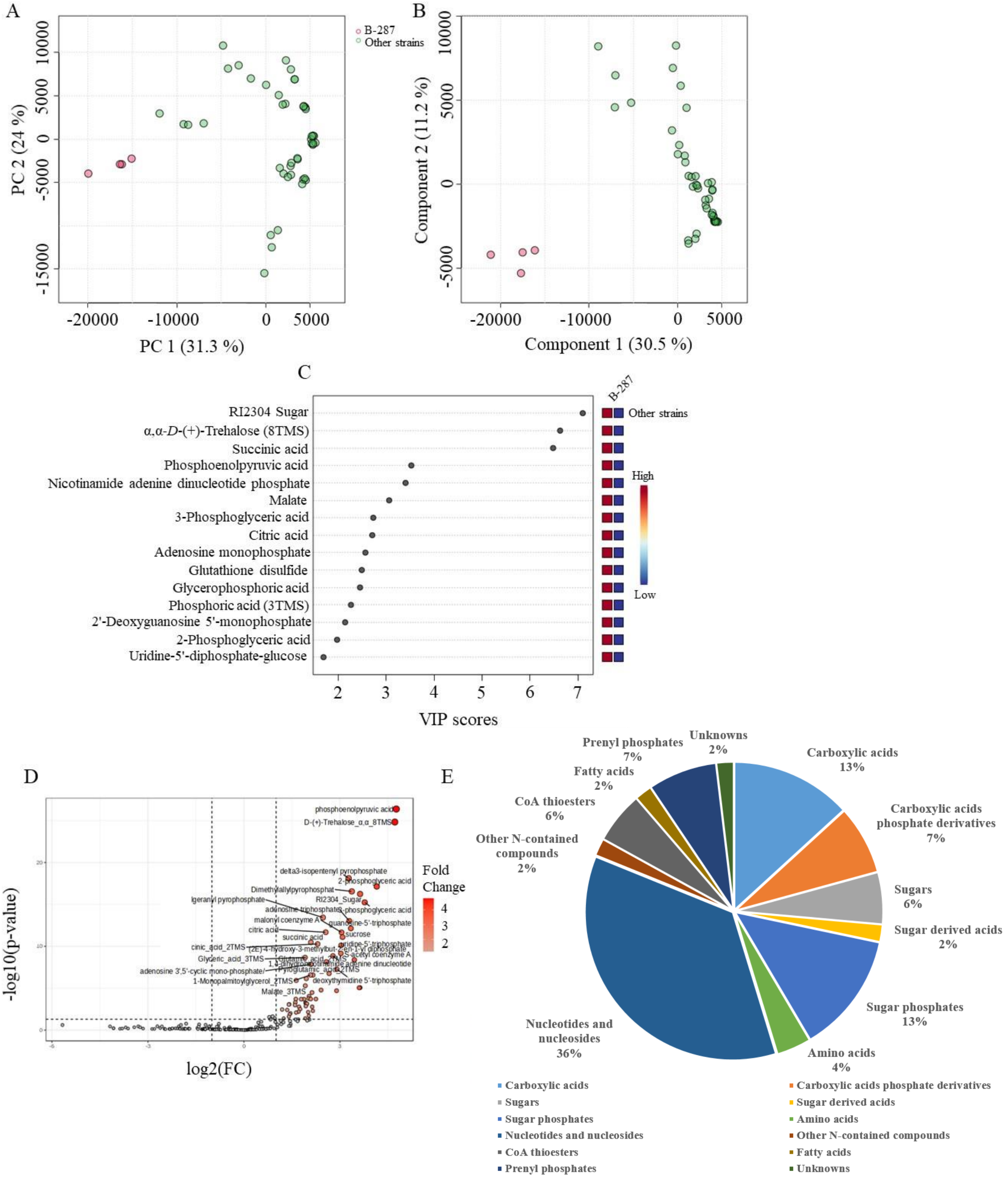

A
B-287
Other strains
PC 2 (24 %)
PC 1 (31.3 %)
B
Component 2 (11.2 %)
Component 1 (30.5 %)
C
B-287
Other strains
RI2304 Sugar
α,α-D-(+)-Trehalose (8TMS)
Succinic acid
Phosphoenolpyruvic acid
Nicotinamide adenine dinucleotide phosphate
Malate
3-Phosphoglyceric acid
Citric acid
Adenosine monophosphate
Glutathione disulfide
Glycerophosphoric acid
Phosphoric acid (3TMS)
2'-Deoxyguanosine 5'-monophosphate
2-Phosphoglyceric acid
Uridine-5'-diphosphate-glucose
High
Low
VIP scores
D
phosphoenolpyruvic acid
D-(+)-Trehalose_α,α_8TMS
Fold Change
-log10(p-value)
log2(FC)
E
Carboxylic acids 13%
Carboxylic acids phosphate derivatives 7%
Sugars 6%
Sugar derived acids 2%
Sugar phosphates 13%
Amino acids 4%
Nucleotides and nucleosides 36%
Other N-contained compounds 2%
CoA thioesters 6%
Fatty acids 2%
Prenyl phosphates 7%
Unknowns 2%
Carboxylic acids
Carboxylic acids phosphate derivatives
Sugars
Sugar derived acids
Sugar phosphates
Amino acids
Nucleotides and nucleosides
Other N-contained compounds
CoA thioesters
Fatty acids
Prenyl phosphates
Unknowns

F

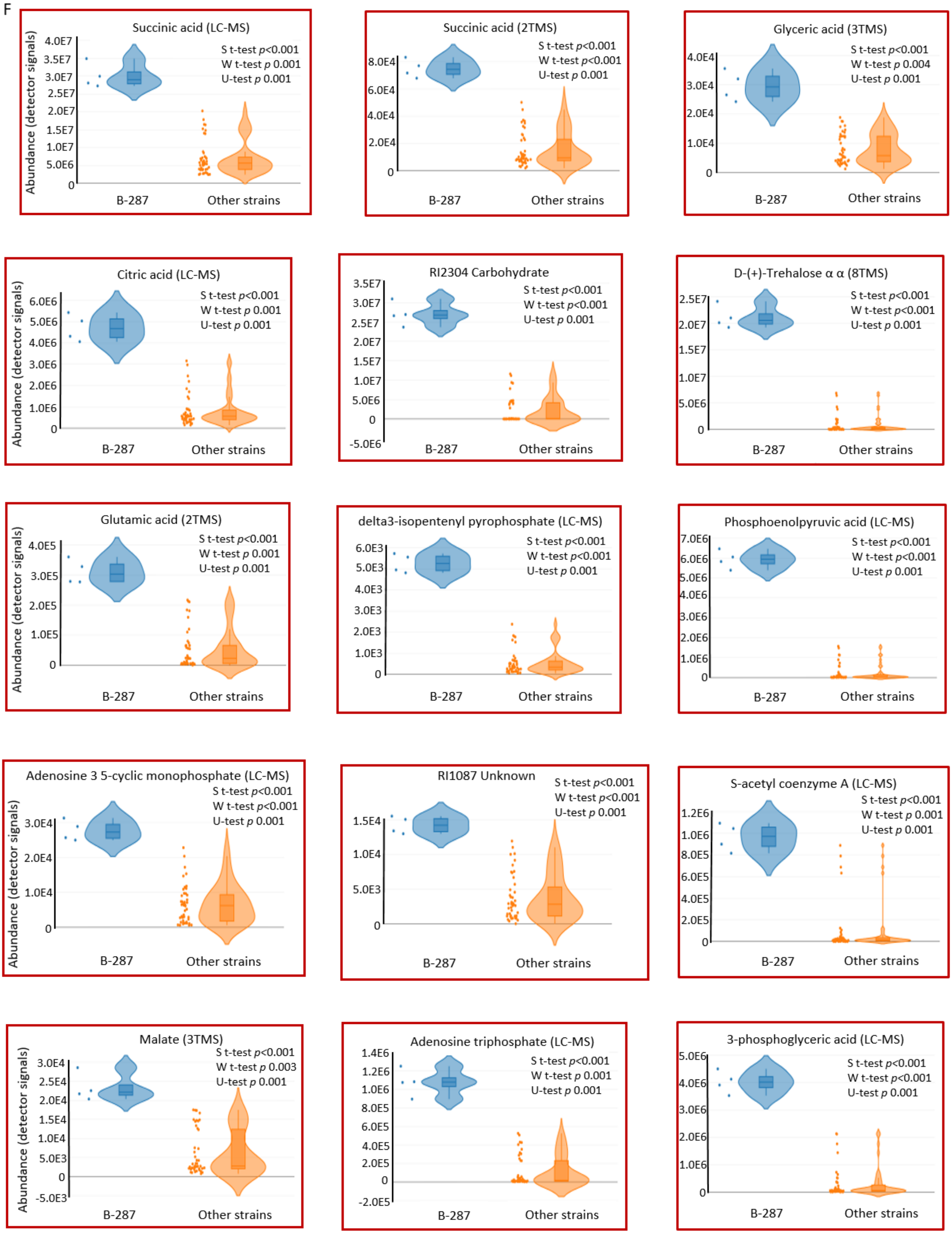

Abundance (detector signals)
Succinic acid (LC-MS)
S t-test p<0.001
W t-test p 0.001
U-test p 0.001
B-287
Other strains
Succinic acid (2TMS)
S t-test p<0.001
W t-test p<0.001
U-test p 0.001
Glyceric acid (3TMS)
S t-test p<0.001
W t-test p 0.004
U-test p 0.001
Citric acid (LC-MS)
S t-test p<0.001
W t-test p 0.001
U-test p 0.001
RI2304 Carbohydrate
S t-test p<0.001
W t-test p<0.001
U-test p 0.001
D-(+)-Trehalose α α (8TMS)
S t-test p<0.001
W t-test p<0.001
U-test p 0.001
Glutamic acid (2TMS)
S t-test p<0.001
W t-test p 0.001
U-test p 0.001
delta3-isopentenyl pyrophosphate (LC-MS)
S t-test p<0.001
W t-test p<0.001
U-test p 0.001
Phosphoenolpyruvic acid (LC-MS)
S t-test p<0.001
W t-test p<0.001
U-test p 0.001
Adenosine 3 5-cyclic monophosphate (LC-MS)
S t-test p<0.001
W t-test p<0.001
U-test p 0.001
RI1087 Unknown
S t-test p<0.001
W t-test p<0.001
U-test p 0.001
S-acetyl coenzyme A (LC-MS)
S t-test p<0.001
W t-test p 0.001
U-test p 0.001
Malate (3TMS)
S t-test p<0.001
W t-test p 0.003
U-test p 0.001
Adenosine triphosphate (LC-MS)
S t-test p<0.001
W t-test p 0.001
U-test p 0.001
3-phosphoglyceric acid (LC-MS)
S t-test p<0.001
W t-test p<0.001
U-test p 0.001

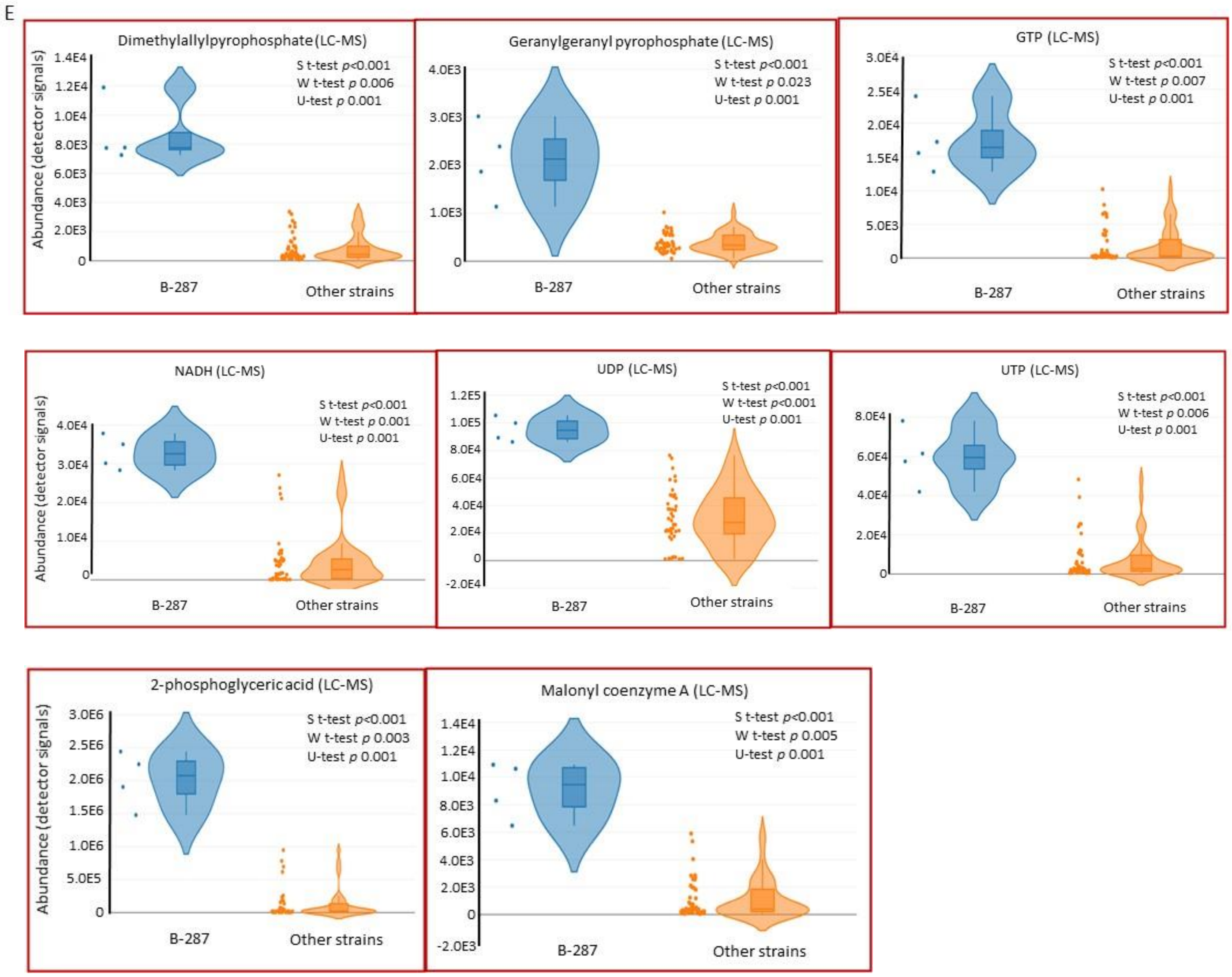

Figure S2-12 Comprehensive statistical analysis of polar metabolite profiles of the extremophilic cyanobacteria strain *Limnospira* sp. B-287 (haloalkaliphilic and natronophilic). This analysis was performed by comparing the metabolite profiles of this strain with the metabolomes of all other cyanobacterial strains studied. PCA (A) and PLS-DA (B) score plots built for the first two principal components, (C) – VIP scores plot based on PLS-DA shows 15 metabolites contributing most to the difference between the compared groups, (D) – Volcano plot illustrates (colored dots) metabolites with statistically significant (t-test, *p*-value ≤0.05, FDR-corrected) difference in relative content between the compared groups (FC ≥2). (E) – Vend diagram presenting the distribution of metabolites identified by the Volcano plot analysis, according to their belonging to specific chemical classes, (F) – Box plots built for the 23 most strain-specific metabolites displaying the most significant difference (S t-test *p*-value (FDR adjusted) <0.01, Welch t-test *p*-value <0.01, U-test *p*-value <0.001) in the content between B-287 and other strains.

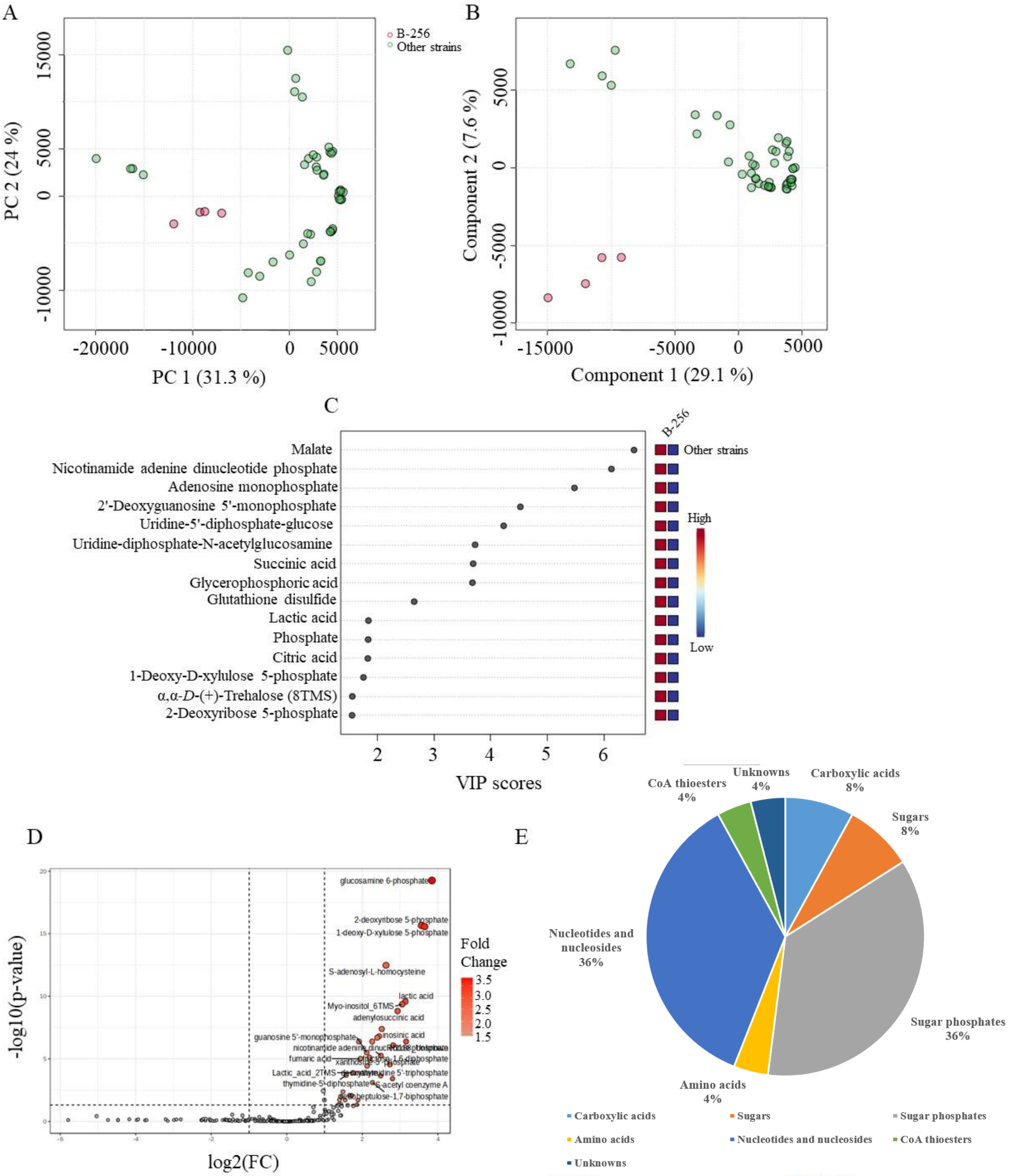

A
B-256
Other strains
PC 2 (24 %)
PC 1 (31.3 %)
-20000
-10000
0
5000
15000
-10000
B
Component 2 (7.6 %)
Component 1 (29.1 %)
-15000
-5000
5000
-10000
C
B-256
Other strains
Malate
Nicotinamide adenine dinucleotide phosphate
Adenosine monophosphate
2'-Deoxyguanosine 5'-monophosphate
Uridine-5'-diphosphate-glucose
Uridine-diphosphate-N-acetylglucosamine
Succinic acid
Glycerophosphoric acid
Glutathione disulfide
Lactic acid
Phosphate
Citric acid
1-Deoxy-D-xylulose 5-phosphate
α,α-D-(+)-Trehalose (8TMS)
2-Deoxyribose 5-phosphate
High
Low
VIP scores
D
-log10(p-value)
log2(FC)
glucosamine 6-phosphate
2-deoxyribose 5-phosphate
1-deoxy-D-xylulose 5-phosphate
S-adenosyl-L-homocysteine
lactic acid
Myo-inositol_6TMS
adenylosuccinic acid
inosinic acid
guanosine 5'-monophosphate
fumaric acid
Lactic_acid_2TMS
thymidine-5'-diphosphate
acetyl coenzyme A
Fold Change
3.5
3.0
2.5
2.0
1.5
E
Unknowns 4%
Carboxylic acids 8%
CoA thioesters 4%
Sugars 8%
Nucleotides and nucleosides 36%
Sugar phosphates 36%
Amino acids 4%
Carboxylic acids
Sugars
Sugar phosphates
Amino acids
Nucleotides and nucleosides
CoA thioesters
Unknowns

F

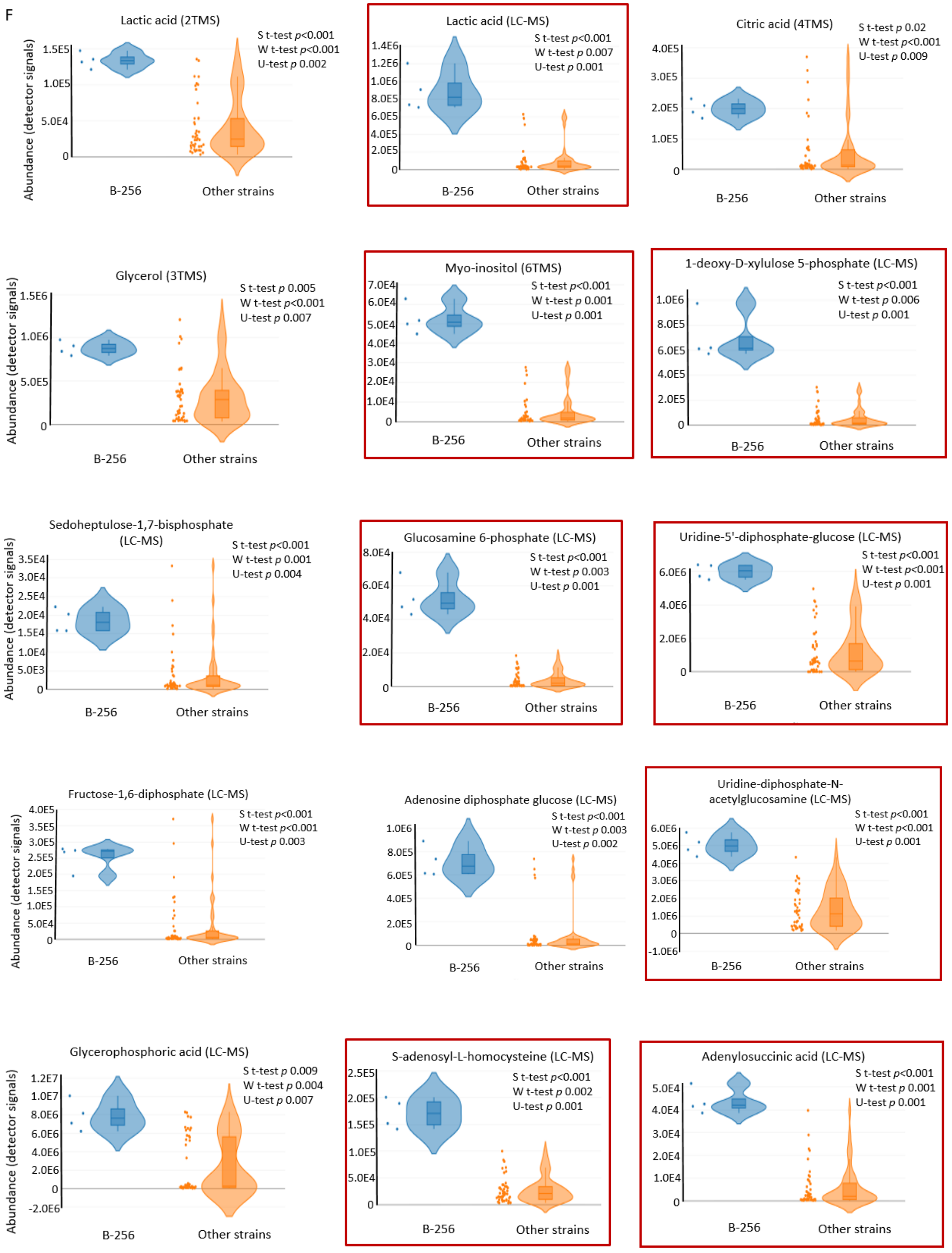

Abundance (detector signals)
Lactic acid (2TMS)
S t-test p<0.001
W t-test p<0.001
U-test p 0.002
B-256
Other strains
Lactic acid (LC-MS)
S t-test p<0.001
W t-test p 0.007
U-test p 0.001
Citric acid (4TMS)
S t-test p 0.02
W t-test p<0.001
U-test p 0.009
Glycerol (3TMS)
S t-test p 0.005
W t-test p<0.001
U-test p 0.007
Myo-inositol (6TMS)
S t-test p<0.001
W t-test p 0.001
U-test p 0.001
1-deoxy-D-xylulose 5-phosphate (LC-MS)
S t-test p<0.001
W t-test p 0.006
U-test p 0.001
Sedoheptulose-1,7-bisphosphate (LC-MS)
S t-test p<0.001
W t-test p 0.001
U-test p 0.004
Glucosamine 6-phosphate (LC-MS)
S t-test p<0.001
W t-test p 0.003
U-test p 0.001
Uridine-5'-diphosphate-glucose (LC-MS)
S t-test p<0.001
W t-test p<0.001
U-test p 0.001
Fructose-1,6-diphosphate (LC-MS)
S t-test p<0.001
W t-test p<0.001
U-test p 0.003
Adenosine diphosphate glucose (LC-MS)
S t-test p<0.001
W t-test p 0.003
U-test p 0.002
Uridine-diphosphate-N-acetylglucosamine (LC-MS)
S t-test p<0.001
W t-test p<0.001
U-test p 0.001
Glycerophosphoric acid (LC-MS)
S t-test p 0.009
W t-test p 0.004
U-test p 0.007
S-adenosyl-L-homocysteine (LC-MS)
S t-test p<0.001
W t-test p 0.002
U-test p 0.001
Adenylosuccinic acid (LC-MS)
S t-test p<0.001
W t-test p 0.001
U-test p 0.001

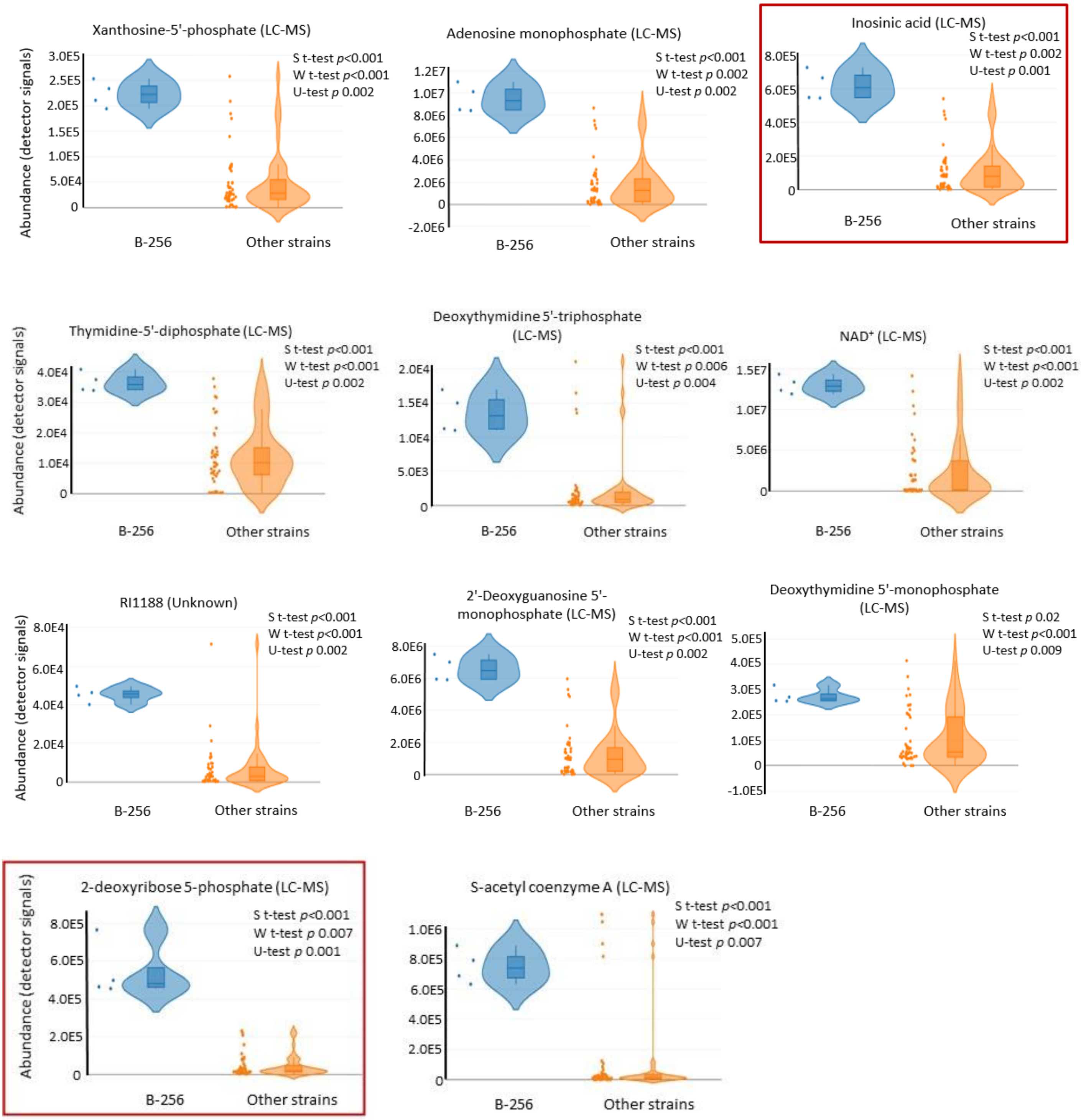

Figure S2-13 Statistical analysis of polar metabolite profiles of the haloalkaliphilic and natronophilic cyanobacteria strain *Limnospira* sp. B-256. This analysis was performed by comparing the metabolite profiles of this strain with the metabolomes of all other cyanobacterial strains studied. PCA (A) and PLS-DA (B) score plots built for the first two principal components, (C) – VIP scores plot based on PLS-DA shows 15 metabolites contributing most to the difference between the compared groups, (D) – Volcano plot illustrates (colored dots) metabolites with statistically significant (t-test, *p*-value ≤0.05, FDR-corrected) difference in relative content between the compared groups (FC ≥2). (E) – Vend diagram presenting the distribution of metabolites identified by the Volcano plot analysis, according to their belonging to specific chemical classes, (F) – Box plots built for the strain-specific metabolites validated additionally by Welch's t-test (W test *p*-value ≤0.05) and Mann-Whitney U-test (U test *p*-value ≤0.01). The red frame indicates ten metabolites displaying the most significant difference (S t-test *p*-value (FDR adjusted) <0.01, Welch t-test *p*-value <0.01, U-test *p*-value <0.001) in the content between B-256 and other strains.

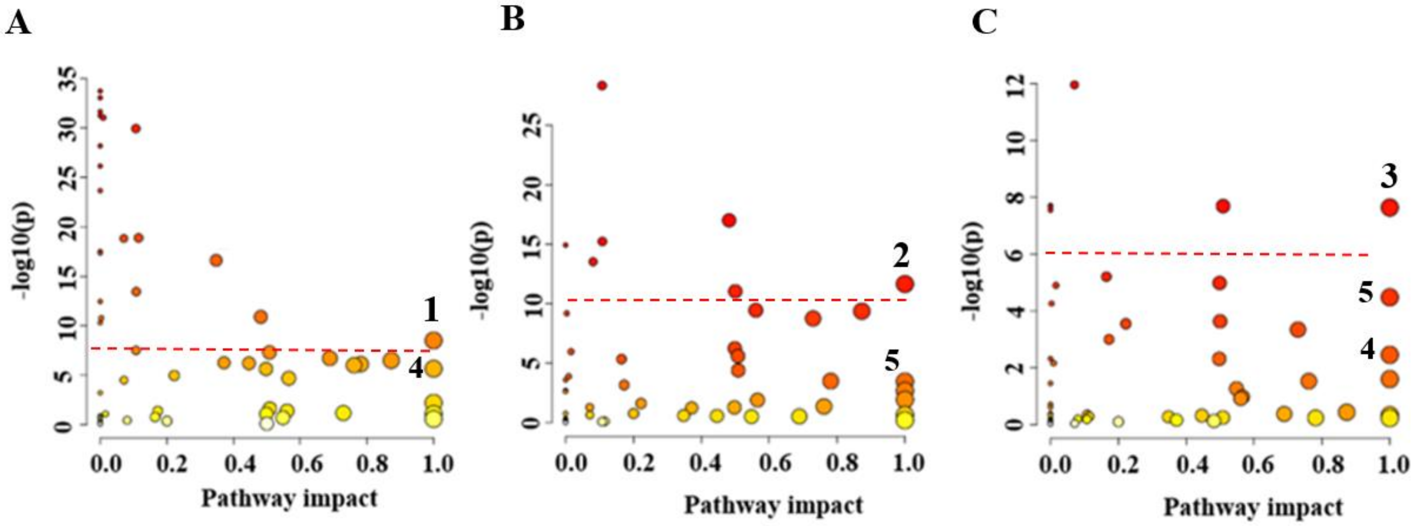

Figure S2-14 Results of the pathway analysis accomplished for haloalkali- and natronophilic cyanobacteria strains B-1529 (A), B-287 (B) and B-256 (C). The most significant metabolic pathways were identified by highest pathway impact (PI) value = 1 and -log10(*p*) value exceeding 5 (indicated by the dotted line): 1 –Alanine, aspartate and glutamate metabolism, 2 – Butanoate metabolism, 3 – Pentose and glucuronate interconversions. Other numerical representations delineate the other preeminent metabolic pathways (PI=1, *p*-value<0.05) which predominantly underlie the tolerance exhibited by strains towards extreme environmental conditions: 4 – Galactose metabolism, 5 – Inositol phosphate metabolism. For the list of significant pathways and their corresponding key metabolites, see Table S2–8.

Literature

1. Harvey, D. J.; Vouros, P., Mass spectrometric fragmentation of trimethylsilyl and related alkylsilyl derivatives. *Mass spectrometry reviews* **2020,** 39, (1-2), 105-211.
2. Benjamini, Y.; Hochberg, Y., Controlling the false discovery rate: a practical and powerful approach to multiple testing. *Journal of the Royal statistical society: series B (Methodological)* **1995,** 57, (1), 289-300.
